# Efficacy and safety of subcutaneous semaglutide in adults with overweight or obese: a subgroup meta-analysis of randomized controlled trials

**DOI:** 10.3389/fendo.2023.1132004

**Published:** 2023-06-26

**Authors:** Rui Zhang, Qin-chuan Hou, Bing-hong Li, Ling Deng, Yu-mei Yang, Ting-xin Li, Xiao-qin Yao, Liang-liang Yang, Xi-long Lin, Yi-qian Liao, Lin Wang, Yu-ping Liu, Jing Tan, Zheng-wei Wan, Ping Shuai

**Affiliations:** ^1^ Department of Health Management & Physical Examination, Sichuan Provincial People’s Hospital, University of Electronic Science and Technology of China, Chengdu, China; ^2^ School of Public Health, Southwest Medical University, Luzhou, China; ^3^ School of Medicine, University of Electronic Science and Technology of China, Chengdu, China; ^4^ Chinese Evidence-based Medicine Center and National Clinical Research Center for Geriatrics, West China Hospital, Sichuan University, Chengdu, Sichuan, China

**Keywords:** semaglutide, overweight, obese, meta-analysis, subgroup

## Abstract

**Introduction:**

Semaglutide shows significant performance on weight reduction in several clinical trials. However, it is not clear what kind of administration frequency or dosage will achieve better effects. This study aims to explore the different therapeutic effect of semaglutide on weight control under the diverse administration circumstances.

**Methods:**

The PubMed, Embase, Web of Science, Cochrane Library, and the Clinical Trials.gov were searched from inception until 6 June, 2022 to include randomized controlled trials evaluating the Efficacy and safety of subcutaneous semaglutide in overweight or obese adults. Random effects or fixed effects model was conducted based on the heterogeneity among trials. Subgroup analysis was performed to identify the detailed effects under different intervention situations.

**Results and discussion:**

Our study included 13 RCTs involving 5,838 participants with 3,794 ones in semaglutide group and 2,044 in placebo group. Semaglutide was associated with a significant reduction on weight loss related outcomes, including the absolute value of weight loss (WMD -8·97, 95% CI -10·73 to -7·21), percentage of weight loss (WMD -10·00, 95% CI -11·99 to -8·00), body mass index (WMD-3·19, 95% CI -4·02 to -2·37) and waist circumference (WMD -7·21,95% CI -8·87 to -5·56). Subgroup analyses illustrated participants with high weekly dosage, long-term treatment duration and severe baseline BMI (Class II obesity) had a more remarkably decreasing on the main outcomes of weight loss (P for interaction<0·05). Total adverse reactions occurred more frequently in the daily administration group than that in the weekly group (P for interaction =0·01). During the treatment, the incidence rate of hypoglycemia was higher in the group without lifestyle intervention compared with that with lifestyle intervention (P for interaction =0·04). Interpretation Subcutaneous semaglutide had significant benefits on weight loss with reasonable safety in overweight or obese adults. Moreover, additional benefits on cardiometabolic profiles were also seen. We recommended semaglutide treatment to be coupled with lifestyle interventions, and target dose of 2·0 mg or more subcutaneously once weekly. Clinicians can choose suitable treatment schemes based on diverse individual situations.

**Systematic review registration:**

https://www.crd.york.ac.uk/PROSPERO/display_record.php?RecordID=337099, identifier PROSPERO (CRD42022337099).

## Graphical Abstract

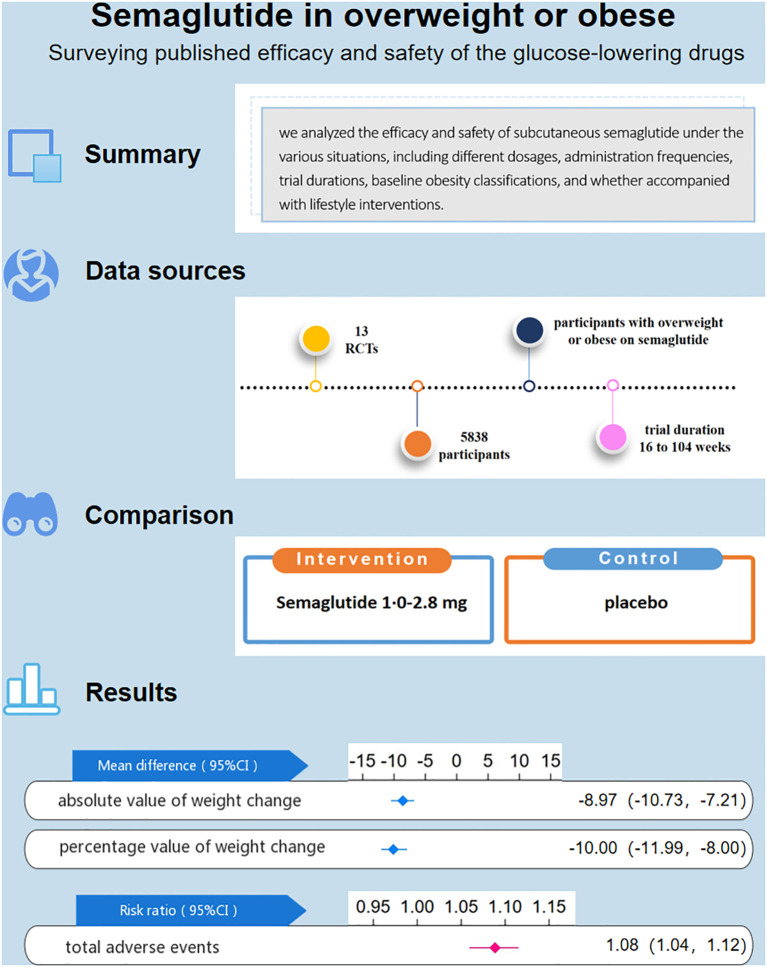

## Summary

This systematic review and meta-analysis aims to explore the different therapeutic effect of semaglutide on weight control under the diverse administration circumstances. Semaglutide was associated with a significant reduction on weight loss related outcomes, including the absolute value of weight loss (WMD -8·97, 95% CI -10·73 to -7·21), percentage of weight loss (WMD -10·00, 95% CI -11·99 to -8·00), body mass index (WMD -3·19, 95% CI -4·02 to -2·37) and waist circumference (WMD -7·21,95% CI -8·87 to -5·56). Subgroup analyses illustrated participants with high weekly dosage, long-term treatment duration and severe baseline BMI (Class II obesity) had a more remarkably decreasing on the main outcomes of weight loss (P for interaction <0·05). Total adverse reactions occurred more frequently in the daily administration group than that in the weekly group (P for interaction =0·01). During the treatment, the incidence rate of hypoglycemia was higher in the group without lifestyle intervention compared with that with lifestyle intervention (P for interaction =0·04). Subcutaneous semaglutide had significant benefits on weight loss with reasonable safety in adults with overweight or obese. Moreover, additional benefits on cardiometabolic profiles were also seen. We recommended semaglutide treatment to be coupled with lifestyle interventions, and target dose of 2·0 mg or more subcutaneously once weekly. Clinicians can choose suitable treatment schemes based on diverse individual situations.

## 1 Introduction

Obesity, as an identified chronic disease, has become a severe public health challenge worldwide. There are more than 1·9 billion overweight adults and 650 million obese ones globally, according to the World Health Organization (1). Multiple studies have shown that more than 200 co-existing diseases are associated with obesity. Consequently, obesity will lead to a higher risk of early mortality and increasing of all-cause mortality, which gives heavy health burden and financial cost to the population of the whole world. Keeping the weight loss to 5% to 15% can improve situations of the majority of diseases associated with overweight or obesity (2–4).

Maintaining long-term weight control is challenging due to the metabolism variation (5) and non-adherence to lifestyle modification (6, 7). Traditional diet restriction and exercise interventions for weight reduction are sometimes difficult to adhere to, and likely to rebound (8–10). Therefore, it comes increasingly urgent to seek a more safe, effective, and long-term way of weight control. Currently, many guidelines and recommendations encourage the use of anti-obese medications to achieve this goal (11, 12).

Several anti-obesity drugs, such as orlistat, naltrexone-bupropion, phentermine-topiramate and liraglutide, have provided sufficient evidence on weight loss for adults (13). A comprehensive network meta-analysis of pharmacotherapy compared with lifestyle modification only, demonstrated phentermine-topiramate and GLP-1 RAs achieved the best effects (14). GLP-1 is an incretin hormone secreted mainly by intestinal L cells, which can increase the insulin release and inhibit the glucagon release in a manner of glucose-dependent way (15). The mechanisms are mainly focused on appetite restriction and food intake reduction by turning up the satiation signals and turning down the hunger signals in the arcuate nucleus of the hypothalamus (16, 17).

Semaglutide, a novel, long-acting GLP-1 RAs, shows significant performance on weight reduction in several clinical trials (18–20). Besides the good performance on weight loss and hypoglycemic effect, it also demonstrates cardio-protective potential (21, 22), greatly broadening its applications on type 2 diabetes treatment only. Compared with exenatide, dulaglutide and liraglutide, semaglutide shows a superior capability of weight-lowering with similar low risk of adverse events (23–25). Therefore, FDA has officially approved it used for adults with overweight or obese in June 2021 (26). In view of the good performance on weight management and maintenance, many trials with different administration ways (oral or subcutaneous, high or low dosages, long or short treatment durations) has been implemented across the North America, Europe and Asian (27–36). Up to now, it is known that the effect of subcutaneous injection of semaglutide is far better than that of oral medication. However, there is no clear conclusion on the therapeutic effect of semaglutide under the diverse application circumstances (dosages, administration frequencies, trial durations, etc.).

Here, in this study, we conducted a comprehensive systematic review and meta-analysis to provide an up-to-date evaluation of semaglutide on efficacy and safety of weight loss, especially under the different administration situations, such as the weekly dosages, administration frequencies, trial durations, baseline obesity classifications and whether accompanied with lifestyle interventions.

## 2 Methods

### 2.1 Protocol registration

This study was implemented according to a priori-written protocol registered in PROSPERO (CRD42022337099, registered 13 June 2022) and the reporting was followed by the PRISMA (**Table S1**). GRADE criteria for meta-analysis were used to assess the quality of evidence.

### 2.2 Eligibility criteria and relevant outcomes

Studies which answered the most important question (regarding the efficacy and safety of subcutaneous semaglutide treatment on weight loss) and report the experiences of adults (age ≥18 years) with overweight or obese (BMI ≥25kg/m^2^) who were prescribed semaglutide treatment for weight management in randomized controlled trials were included.

Outcomes for the first key question were the weight-related indicators [the absolute change and percentage change of body weight, change in BMI and waist circumference, weight loss targets (5%, 10% and 15%) from the baseline to the end of intervention]. Outcomes for the second key question were cardiometabolic parameters, such as change in SBP and DBP, HbA1c, FPG, TC, TG, CRP, and patient-reported outcomes of quality of life, using physical functioning scores by the SF-36v2 and physical function scores by the IWQOL-Lite-CT. Outcomes for the third key question were safety outcome measures mainly referred to adverse events occurring with treatment, including constipation, nausea, vomiting, diarrhea, decreased appetite, hypoglycaemia, and severe side-effects.

To capture the broad field of literature, participants with obesity-related complications were included in this study. For instance, we included overweight or obese people with type 2 diabetes, non-alcoholic fatty liver disease, polycystic ovary syndrome, et al. Studies were excluded if they included patients using semaglutide dosage less than 1.0 mg once weekly, or less than 12 weeks. Non-randomized studies, animal trials, protocols, conference abstracts, case reports and *post-hoc* analyses were excluded.

### 2.3 Search strategy

The literature was systematically searched for English articles published up to June 6, 2022 from the following databases which were PubMed, Embase, Web of Science and Cochrane Library. The international or national clinical trial registries, including the ClinicalTrials.gov, European Union Clinical Trials Register, World Health Organization-International Clinical Trials Registry Platform and 9 national clinical trial registries were also searched. The search strategy involved using the medical subject heading terms and the terms of “semaglutide”, “wegovy”, “ozempic”, “overweight”, “obesity” and “randomized controlled trial”. The reference lists from published articles and trials were manually retrieved and screened to identify relevant additional studies. Detailed online search strategies were seen in **Table S2** and **Text S1**.

### 2.4 Study selection and data extraction

Two authors (RZ, QH) independently reviewed and extracted the data and information through a pre-planned standardized form in line with the Cochrane handbook for systematic reviews of intervention. Disagreement was resolved by group discussion or by consulting with the other two authors (XL, YL) until consensus was reached. The following information was derived from each of the trials, including the publication details (clinical trial number and trial phase, source of funding, author, year of publication, country), participant profiles (age and BMI at baseline, percentage of female, ethnicity, comorbidity), treatment characteristics (sample size, placebo controlled, trial duration, dosage, with or without lifestyle intervention), and outcome measures. Treatments of all the included trials were based on the full-analysis set and ITT principle. If both exist, ITT was preferred.

### 2.5 Risk of bias assessment

The risk of bias was assessed by two reviewers (LY, YY) respectively using the Cochrane Collaboration risk of bias tool for RCTs. It involved six aspects, which were the bias generated by the randomization process, the bias generated by the randomization sequence, whether the blind method was implemented between participants and researchers, the bias of result evaluation, the bias caused by incomplete result data, and the bias of selective reporting results. We classified the risk of bias into three levels which was high, low and unclear. If one aspect of the study was identified as low risk, we would judge the overall risk of bias as low. The discordance between two reviewers was resolved through discussion with another reviewer (ZW) until all the researchers reached a consensus.

### 2.6 Data synthesis and statistical analysis

The results of the systematic evaluation were summarized from quantitative aspects. For the continuous variables, the mean difference and standard deviation were extracted from the intervention and placebo group respectively, using the WMD to estimate the treatment effect of the randomized controlled trials. For the dichotomous variables, we counted the number of cases, using the average RR to estimate the risk ratio.The WMD and RR with 95% CI were calculated for the continuous and dichotomous outcomes respectively, to represent the pooled effects and safety conditions of semaglutide on outcomes of interest. The heterogeneity among individual studies was estimated by the Cochran Q test and quantified by I2 statistic. We used a random effect model based on the DerSimonian-Laird method for those variables with significant heterogeneity (P value <0·1 by Q-test or I2 value ≥50%). Otherwise, a fixed effect model based on the inverse-variance method was applied for trials with no heterogeneity.

Subgroup analysis was performed to identify the detailed effects under different intervention situations [(Class I obesity: 30 kg/m^2^ ≤ BMI <35 kg/m^2^, Class II obesity: 35 kg/m^2^ ≤ BMI <40 kg/m^2^), administration frequency (weekly or daily), weekly cumulative dose (low dose: 1·0-2·1 mg, high dose: 2·4-2·8 mg), trial duration (short-term: <52 weeks, long-term: ≥52 weeks) and lifestyle interventions (with or without)]. Meta-regression was carried out to explore the possible sources of heterogeneity, such as the baseline degree of obesity involved above (37). Potential publication bias of the included trials was tested by the Egger’s test and inspection of funnel plots. Sensitivity analysis was carried out by excluding studies with high-risk bias each time to explore whether the results were significantly deviated by a specific study.

Statistical analyses were performed using Stata version 16·0 (Stata Corp, College Station, TX, USA) and R software (version 4·1·3). The threshold for significance was set as P <0·05 with a two-sided test.

### 2.7 Grading of evidence GRADE

The GRADE framework was used to estimate the certainty of evidence on relevant outcomes of interest. Three reviewers (RZ, QH, PS) graded the strength of evidence as high, moderate, low and very low degree, following five assessment criteria which were risk of bias, inconsistency, indirectness, publication bias, and imprecision. Each reviewer was required to describe in detail the reasons that downgrading the classification of evidence based on the principle of evaluation. GRADE pro 3·6 software was used to do the record and rank for the quality of evidence on all outcome measures. Missing data information was requested by email from the corresponding authors. If no response was obtained, we derived the data followed by the rules of the Cochrane handbook of systematic reviews of intervention.

## 3 Results

### 3.1 Identification and study selection

**Figure 1:**
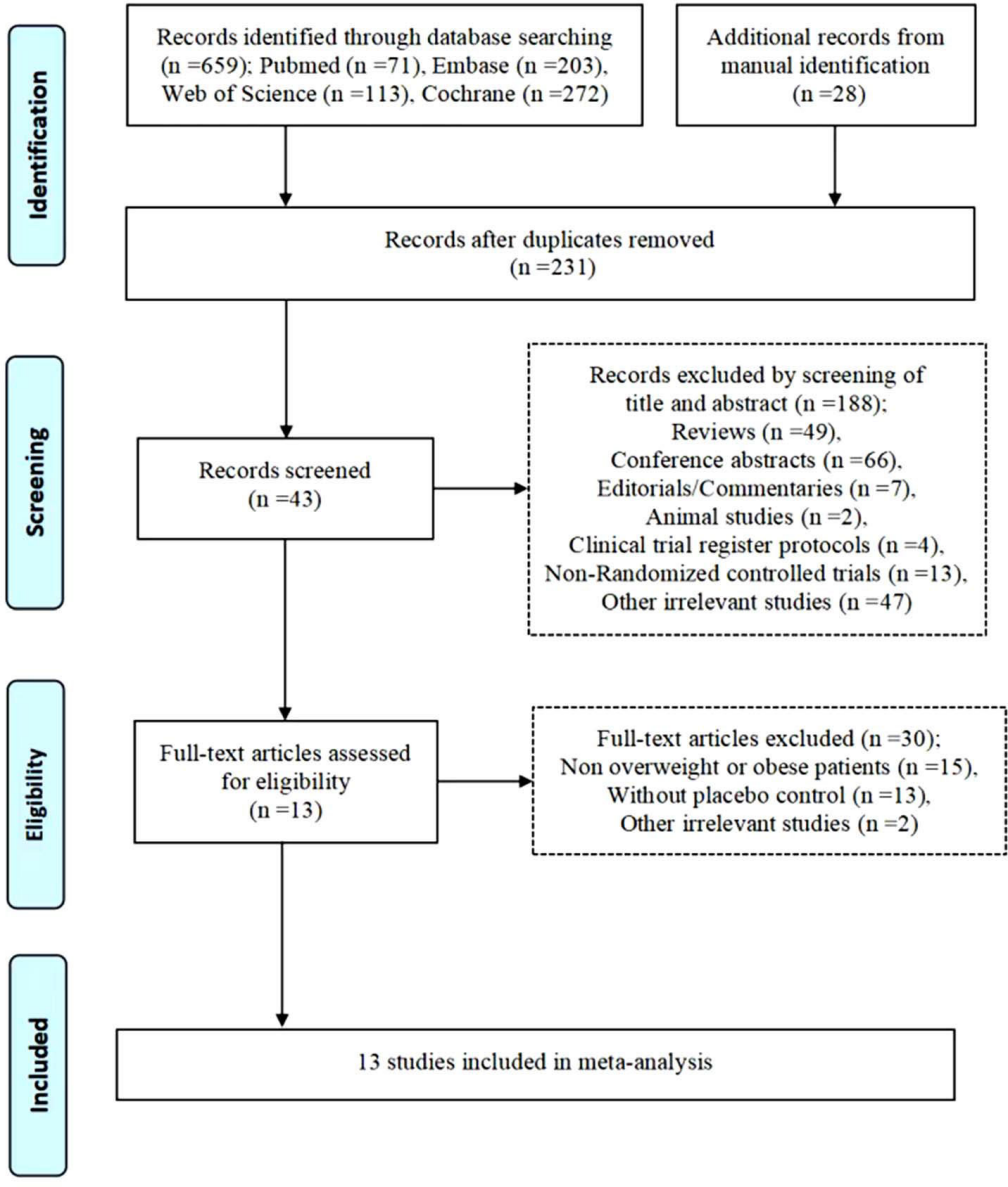
Flow diagram of study selection process.

Of 659 potential eligible records were identified according to the search strategy. Among them, 231 duplicate studies and 188 articles which did not meet the inclusion criteria were excluded. Finally, a total of 13 randomized controlled trials involving 5838 participants were included in the final meta-analysis (**Figure 1**) (18–20, 27–36). All the articles were randomized controlled trials focused on adults with overweight or obese with or without comorbidity that were published from September 2017 to March 2022.

The baseline obesity classification fluctuated greatly from 31·9 to 39·3 kg/m2. The comorbidity of 8 studies was not clear, 5 studies had definite specifications (1 with polycystic ovary syndrome, 2 with diabetes, and 2 with nonalcoholic fatty liver disease). Among the 13 studies, 10 were multi-center studies and 3 were single-center ones, 10 were multi-ethnic studies and 3 were single ethnic ones (Slovenians, Caucasians and Asians). In general, most of the studies were implemented in the U.S and the majority of participants were Caucasians. The sample size of the 13 studies varied from a minimum number of 30 to a maximum of 1,961. The trial duration of the trials was from 16 to 104 weeks. Twelve of them were funded by a pharmaceutical company entirely with only one was partly funded by a non-profit organization. The characteristics of the 13 studied were described in **Table 1**.

**Table 1:** Individual participant characteristics of included studies.

Trial name	Clinical Trial No	Blinding	Study duration	Trial Phase	Study arms	Sample Size, n	Baseline age (years)	Baseline BMI(kg/m^2^)	Female, n (%)
Blundell 2017	NCT02079870	Double-blind	12 weeks	Phase 1	Semaglutide 1·0 mg	30	42	33·8	10 (33·3)
					Placebo	28			
Lingvay 2018	NCT02461589	Double-blind	26 weeks	Phase 2	Semaglutide 1·4 mg	65	58·4 ± 9·6	32·8 ± 4·5	22 (33·9)
					Semaglutide 2·1 mg	63	54·8 ± 9·7	33·1 ± 4·7	31 (49·2)
					Placebo	129	57·1 ± 9·2	32·8 ± 4·2	57 (44·2)
ONeil 2018	NCT02453711	Double-blind	52 weeks	Phase 2	Semaglutide 1·4 mg	103	44 ± 11	40·1 ± 6·9	66 (64·1)
					Semaglutide 2·1 mg	103	47 ± 12	39·6 ± 7·1	66 (64·1)
					Semaglutide 2·8 mg	102	48 ± 13	39·9 ± 8·8	66 (64·7)
					Placebo	136	46 ± 13	40·1 ± 7·2	88 (64·7)
Newsome 2020	NCT02970942	Double-blind	72 weeks	Phase 2	Semaglutide 1·4 mg	78	58·1 ± 9·9	35·6 ± 6·1	52 (67.0)
					Semaglutide 2·8 mg	82	54·3 ± 10·2	35·2 ± 6·6	47 (57.0)
					Placebo	80	52·4 ± 10·8	36·1 ± 6·6	44 (55.0)
Jensterle 2021	NCT04263415	Single-blind	16 weeks	Phase 4	Semaglutide 1·0 mg	15	33·7 ± 5·3	36·8 ± 3·9	13 (100·0)
					Placebo	15		35·4 ± 3·8	12 (100·0)
Flint 2021	NCT03357380	Double-blind	72 weeks	Phase 2	Semaglutide 2·8 mg	34	59·5 ± 10·1	NA	11 (32·4)
					Placebo	33	60·5 ± 8·5	NA	9 (27·3)
Friedrichsen 2021	NCT03842202	Double-blind	20 weeks	Phase 1	Semaglutide 2·4 mg	36	40·7 ± 12·2	34·2 ± 3·0	12 (33·3)
					Placebo	36	45·0 ± 9·5	34·6 ± 3·1	16 (44·4)
Wadden 2021	NCT03611582	Double-blind	68 weeks	Phase 3	Semaglutide 2·4 mg	407	46 ± 13	38·1 ± 6·7	315 (77·4)
					Placebo	204	46 ± 13	37·8 ± 6·9	180 (88·2)
Wilding 2021	NCT03548935	Double-blind	68 weeks	Phase 2	Semaglutide 2·4 mg	1306	46 ± 13	37·8 ± 6·7	955 (73·1)
					Placebo	655	47 ± 12	38·0 ± 6·5	498 (76·0)
Davies 2021	NCT03552757	Double-blind	68 weeks	Phase 3	Semaglutide 1·0 mg	403	56 ± 10	35·3 ± 5·9	203 (50·4)
					Semaglutide 2·4 mg	404	55 ± 11	35·9 ± 6·4	223 (55·2)
					Placebo	403	55 ± 11	35·9 ± 6·5	190 (47·1)
Kadowaki 2022	NCT03811574	Double-blind	68 weeks	Phase 1	Semaglutide 1·7 mg	101	51 ± 10	31·6 ± 3·7	37 (37.0)
					Semaglutide 2·4 mg	199	52 ± 12	32·0 ± 4·6	85 (43.0)
					Placebo	101	50 ± 9	31·9 ± 4·2	26 (26.0)
Rubino 2022	NCT04074161	Double-blind	68 weeks	Phase 3	Semaglutide 2·4 mg	126	48 ± 14	37·0 ± 7·4	102 (81·0)
					Placebo	85	51 ± 12	38·8 ± 6·5	66 (77·6)
NCT03693430 2022	NCT03693430	Double-blind	104 weeks	Phase 3	Semaglutide 2·4 mg	152	47 ± 12	NA	123 (80·9)
					Placebo	152	47 ± 10	NA	113 (74·3)

NCT, national clinical trial; NA, not available.

### 3.2 Weight loss efficacy outcomes

All the trials displayed a significant effect on body weight loss compared with placebo (**Table 2**). There was a significant correlation between semaglutide treatment and a reduction in absolute weight change (WMD -8·97, 95% CI -10·73 to -7·21; P <0·001, I^2^= 99·15%), percentage of weight change (WMD -10·00, 95% CI -11·99 to -8·00; P <0·001, I^2^= 96·92%), change in BMI from baseline to the end (WMD -3·19, 95% CI -4·02 to -2·37; P <0·001, I^2^= 94·89%), as well as waist circumference (WMD -3·19, 95% CI -4·02 to -2·37; P <0·001, I^2^ = 94·89%). Compared with placebo, semaglutide treatment obtained a higher achievement of the goal of 5% (RR 3·00, 95% CI 2·53 to 3·56; P <0·001, I^2^= 83·29%), 10% (RR 5·04, 95% CI 3·99 to 6·36; P <0·001, I^2^= 70·76%) and 15% (RR 6·95, 95% CI 5·39 to 8·96; P =0·07, I^2^= 42·28%). Details were seen in **Figures S1–7**.

**Table 2:** Effect of Weight loss, cardiovascular metabolic and quality of life in semaglutide compared with placebo control.

	No of trials	n	WMD	95% CI		I2 (%)	P_heterogeneity_	Tau-squared
body weight (kg)	18	5989	-8·97	-10·73	-7·21	99·19	<0·001	13·71
body weight (%)	11	5365	-10·00	-11·99	-8·00	96·92	<0·001	10·72
body mass index (kg/m2)	9	3024	-3·19	-4·02	-2·37	94·89	<0·001	1·43
waist circumference (cm)	13	5400	-7·21	-8·87	-5·56	96·03	<0·001	8·19
weight loss goal (5%)	13	6152	3·00	2·53	3·56	83·29	<0·001	0·07
weight loss goal (10%)	11	5766	5·04	3·99	6·36	70·76	<0·001	0·10
weight loss goal (15%)	11	5766	6·95	5·39	8·96	42·28	0·07	0·07
HbA1c (%)	15	3947	-0·82	-1·03	-0·60	99·06	<0·001	0·18
FPG (mmol/L)	12	3580	-1·18	-1·67	-0·69	98·67	<0·001	0·73
TC (mmol/L)	5	952	-0·30	-0·52	-0·08	0	0·85	–
TG (mmol/L)	5	952	-0·63	-1·02	-0·25	8·05	0·36	–
DBP (mmHg)	12	5373	-2·09	-2·83	-1·34	69·94	<0·001	0·92
SBP(mmHg)	12	5373	-4·82	-5·87	-3·77	62·43	<0·001	1·79
CRP (mg/L)	4	927	-1·37	-1·82	-0·92	0	0·88	–
SF-36v2	6	4300	1·22	0·69	1·74	63·85	0·02	0·24
IWQOL-Lite-CT	5	3756	4·81	1·28	8·34	89·13	<0·001	13·84

**Figure 2:**
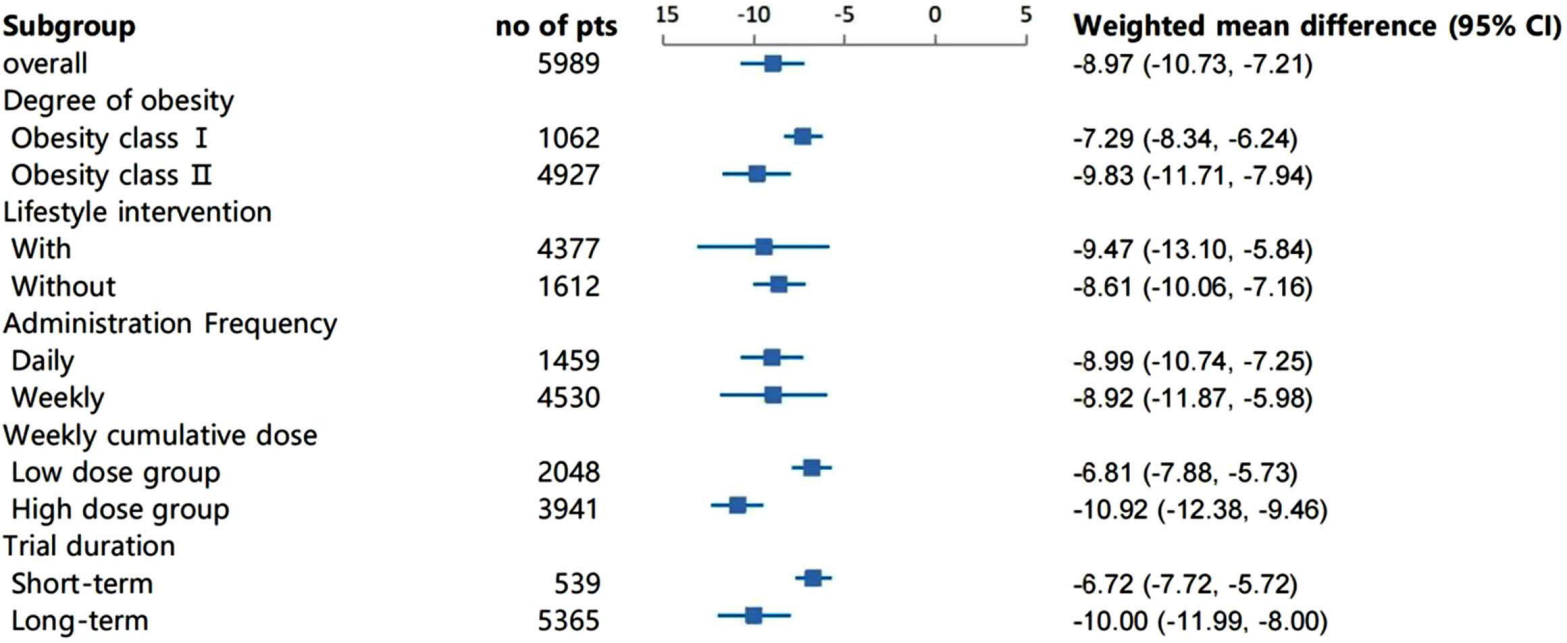
Subgroup analysis of the effect of absolute value of body weight change(kg).

**Figure 3:**
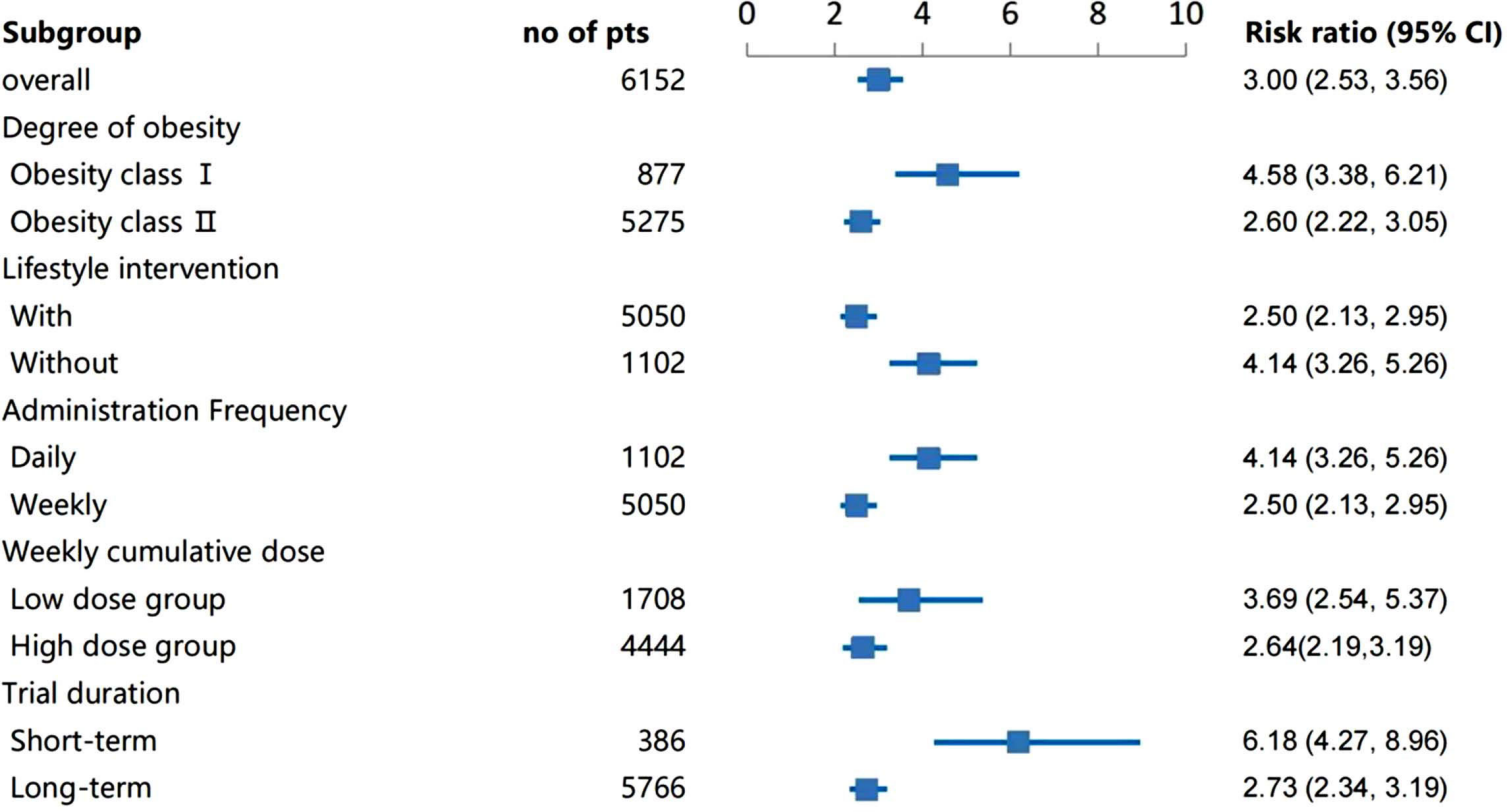
Subgroup analysis of the effect of weight loss goal of 5%.

For absolute value of weight change, subgroup analyses revealed the absolute value of weight change decreased more significantly in the Class II obesity group than that in the Class I obesity group (P for interaction =0·02). Compared with the low cumulative weekly dose group, high weekly dose group had a greater improvement on the weight change (P for interaction <0·01). In addition, the absolute value of weight change decreased more obviously in the long-term treatment group than that in the short-term group (P for interaction <0·01), see **Figure 2**. For waist circumference, subgroup analyses showed that the high weekly semaglutide dosage group had a greater reduction on waist circumference compared with the low dose group (P for interaction =0·01). Participants in ClassIobesity group, whose baseline BMI were 30 to 34·99 kg/m2, achieved a greater proportion of weight loss goal of 5% and goal of 10% than those in Class II obesity group (P for interaction <0·01). For weight loss goals of 5%, subgroup analysis demonstrated that higher achievement rate of weight loss goal of 5% was found in group of short-term treatment with duration less than 52 weeks, group of once daily administration and that of without lifestyle intervention (P for interaction <0·01), see **Figure 3**. No obvious difference on percentage of weight change, BMI reduction,weight loss goals of 10% and 15% was found under the different subgroup. Details were seen in **Tables S3–9**.

### 3.3 Cardiovascular metabolic outcomes

Nine studies had sufficient data of HbA1c. Random effects meta-analyses were conducted and revealed semaglutide treatment had a significant effect on lowering HbA1c level compared with placebo (WMD -0·81, 95% CI -1·05 to -0·57; P < 0·001, I^2^= 98·87%). For 7 studies with FPG data, semaglutide also showed obvious hypoglycemic effects in contrast with placebo (WMD -1·19, 95% CI -1·64 to -0·74, P <0·001, I^2^ = 98·36%). Among 7 studies, obvious reduction was seen on blood pressures both with the DBP (WMD -2·09, 95% CI -2·83 to -1·34; P <0·001, I^2^= 69·94% and SBP (WMD -4·82, 95% CI -5·87 to -3·77; P <0·001, I^2^ = 62·43%). For the serum lipid, although only 5 trials provided sufficient data, the improvements in semaglutide group were still observed both on total cholesterol (WMD -0·30, 95% CI -0·52 to -0·08; P =0·85, I^2^= 0%) and triglyceride (WMD -0·63, 95% CI -1·02 to -0·25; P =0·36, I^2^= 8·05%. 4 trials illustrated that CRP had a distinct reduction after treatment by semaglutide (WMD -1·37, 95% CI -1·82 to -0·92; P =0·88, I^2^ = 0%), see **Table 2** (Details were seen in **Figure S8-14**).

Subgroup analysis proved that participants grouped in Class I obesity at baseline had a greater reduction of HbA1c than those in Class II obesity (P for interaction =0·01) (**Table S10**). Adults with diet control and exercise enhancement had a greater decrease on triglyceride level than those without lifestyle intervention(P for interaction =0·04) (**Table S11**). But for other cardiovascular metabolic indicators, like FPG, TC, blood pressure and CRP, no absolute effect was detected under different subgroups. Details were seen in **Tables S10–16**.

### 3.4 Quality of life outcomes

Four studies reported the SF-36v2, and 3 reported the IWQOL-Lite-CT. The quality of life improved more from the scores of SF-36v2 in the semaglutide group than placebo (WMD 1·22, 95% CI 0·69 to 1·74; P =0·02, I^2^ = 63·85%). Moreover, greater progress in IWQOL-Lite-CT (WMD 4·81, 95% CI 1·28 to 8·34; P <0·001, I^2^= 89·13%) was seen in the treatment group (**Figures S15–16**). No direct association was found between quality of life and medication dosage, intervention duration, treatment frequency and whether accompanied with lifestyle intervention based on the meta-regression results, see **Table 2** (**Tables S17, 18**).

### 3.5 Safety outcomes

11 studies reported the total adverse events and 12 studies reported severe adverse events. The safety evaluation demonstrated that semaglutide treatment had a slightly higher incidence rate of total adverse events compared with placebo (RR 1·08, 95% CI 1·04 to 1·12; P <0·001, I^2^= 69·23%), as well as the rate of discontinuation due to adverse events (RR 1·64, 95% CI 1·30 to 2·06; P =0·41, I^2^ = 3·89%). However, for the severe adverse reactions, no significant difference was noted between two groups (RR 1·16, 95% CI 0·97 to 1·38; P =0·35, I^2^ = 8·38%) (**Figures S17–19** and **Table 3**).

**Table 3:** Risk ratio in safety indicators between the semaglutide and placebo control.

	No of trials	n	RR	95% CI		I^2^ (%)	P_heterogeneity_	Tau-squared
total adverse reactions	17	6756	1·08	1·04	1·12	69·23	<0·001	0
serious adverse reactions	18	6814	1·16	0·97	1·38	8·38	0·35	–
adverse events leading to discontinuation	18	6814	1·64	1·30	2·06	3·89	0·41	–
hypoglycemia	11	5667	212	1·50	2·98	0	0·69	–
vomiting	17	6756	3·79	2·77	5·19	57·01	<0·001	0·20
nausea	17	6756	2·82	2·56	3·11	0	0·68	–
constipation	16	6684	2·66	2·11	3·36	55·60	<0·001	0·10
loss of appetite	9	1688	2·25	1·46	3·46	43·18	0·08	0·18
diarrhea	17	6756	1·90	1·65	2·19	33	0·09	0·03
cardiovascular disorders	7	4893	0·82	0·66	1·02	0	0·80	–
psychic disorders	9	5212	1·06	0·77	1·46	44·28	0·07	0·09
allergic reactions	10	5609	0·97	0·80	1·18	0	0·73	–
injection-site reactions	10	5609	0·81	0·63	1·04	0	0·99	–

The most common adverse events were hypoglycemia and gastrointestinal adverse events. Indeed, the increased risk on the occurrence of hypoglycemia was seen in the semaglutide group (RR 2·13, 95% CI 1·51 to 3·01; P =0·61, I^2^ = 0%) (**Figure S20**). In the meanwhile, the incidence rates of gastrointestinal disorders, such as vomiting, nausea, constipation, loss of appetite and diarrhea were comparatively higher than those in the placebo, with the risk ratios of 3.79 (95% CI 2·77 to 5·19), 2·82 (2·56 to 3·11), 2·66 (2·11 to 3·36), 2·25 (1·46 to 3·46), and 1·90 (1·65 to 2·19), respectively (**Figures S21–25**). No significant difference was found in the followings when compared with placebo group, which were cardiovascular disorders, psychic disorders, allergic reactions, injection-site reactions (**Figures S26–29**).

**Figure 4:**
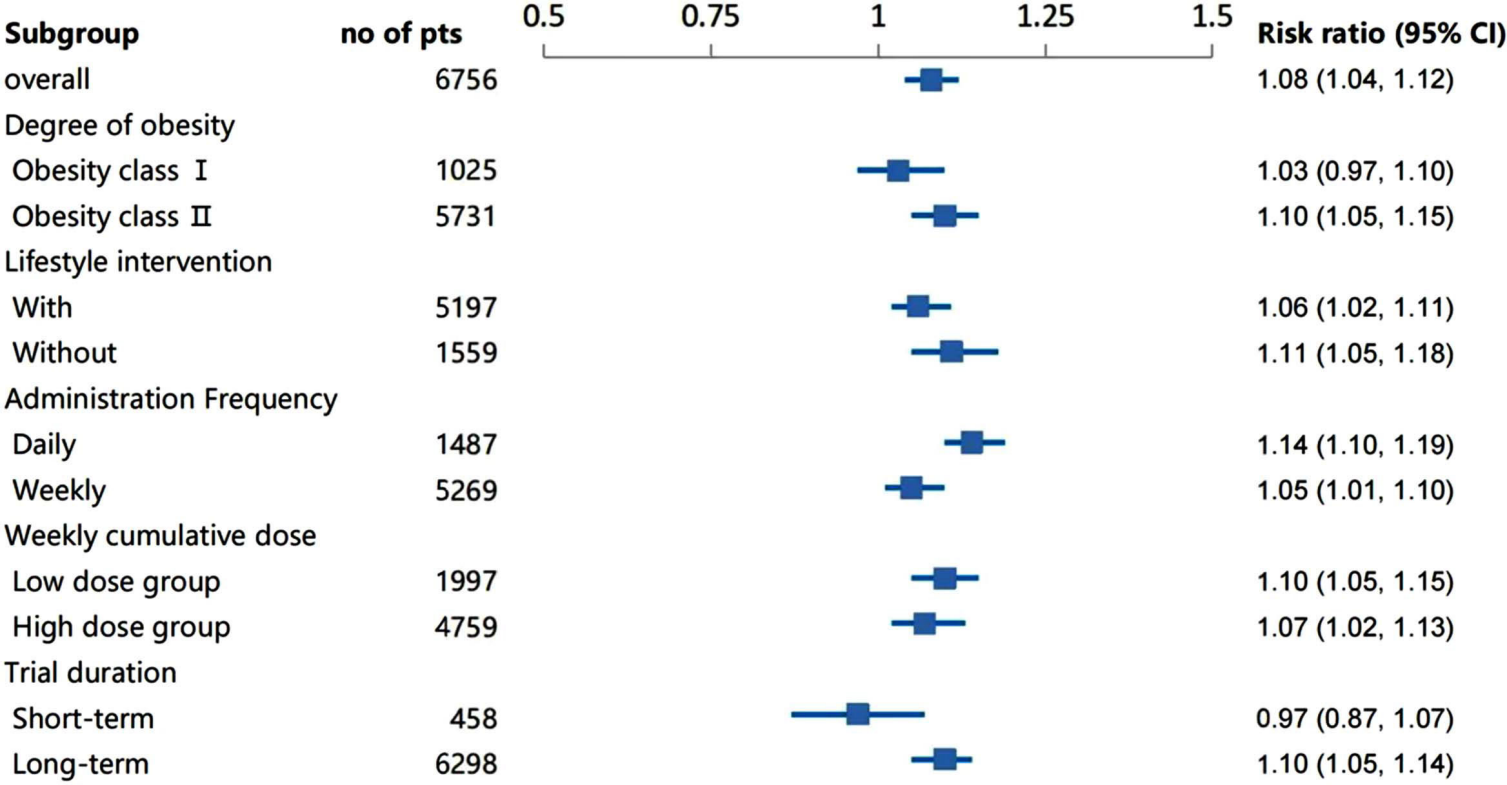
Subgroup analysis of total adverse reactions under different situations.

Subgroup analysis was done for further exploration. Results showed the incidence rate of hypoglycemia was higher in the group without lifestyle intervention, compared with that with lifestyle intervention (P for interaction =0·04). Hypoglycemia (RR 3·53, 95% CI 1·99 to 6·27) and total adverse reactions (RR 1·14, 95% CI 1·10 to 1·19) occurred more frequently in the daily group than that in the weekly group (**Table S19**). Contrasted with the short-term treatment duration group, the long-term treatment group had an increased risk of total adverse reactions (RR 1·10, 95% CI 1·05 to 1·14), vomiting (RR 4·30, 95% CI 3·45 to 5·37) and discontinuation (RR 1·82, 95% CI 1·42 to 2·33), see **Figure 4** (**Tables S20–22**). Generally, heterogeneities were seen for most of the adverse events, except the severe adverse event, nausea and hypoglycemia (**Tables S23–31**).

### 3.6 Grade of evidence

The GRADE evidence grading system showed all of our primary outcome indicators were rated as moderate to high. Of these, BMI, waist circumference and weight loss of at least 15% were considered to have high quality levels (**Tables S32, 33**). Due to the inconsistency and publication bias, the quality of evidence on absolute and percentage of weight loss, weight loss targets of at least 5% and 10% were degraded to moderate level.

### 3.7 Sensitivity analysis

A sensitivity analysis was conducted for the weight related indicators (e.g., the absolute and percentage change of body weight, weight loss target of at least 5%, BMI and waist circumference). High risk studies were sequentially removed. The cumulative statistics for all comparisons of all subjects illustrated the summary results did not change dramatically, suggesting our analysis was generally robust (**Figures S30–34**).

### 3.8 Risk and bias assessment

**Figure 5:**
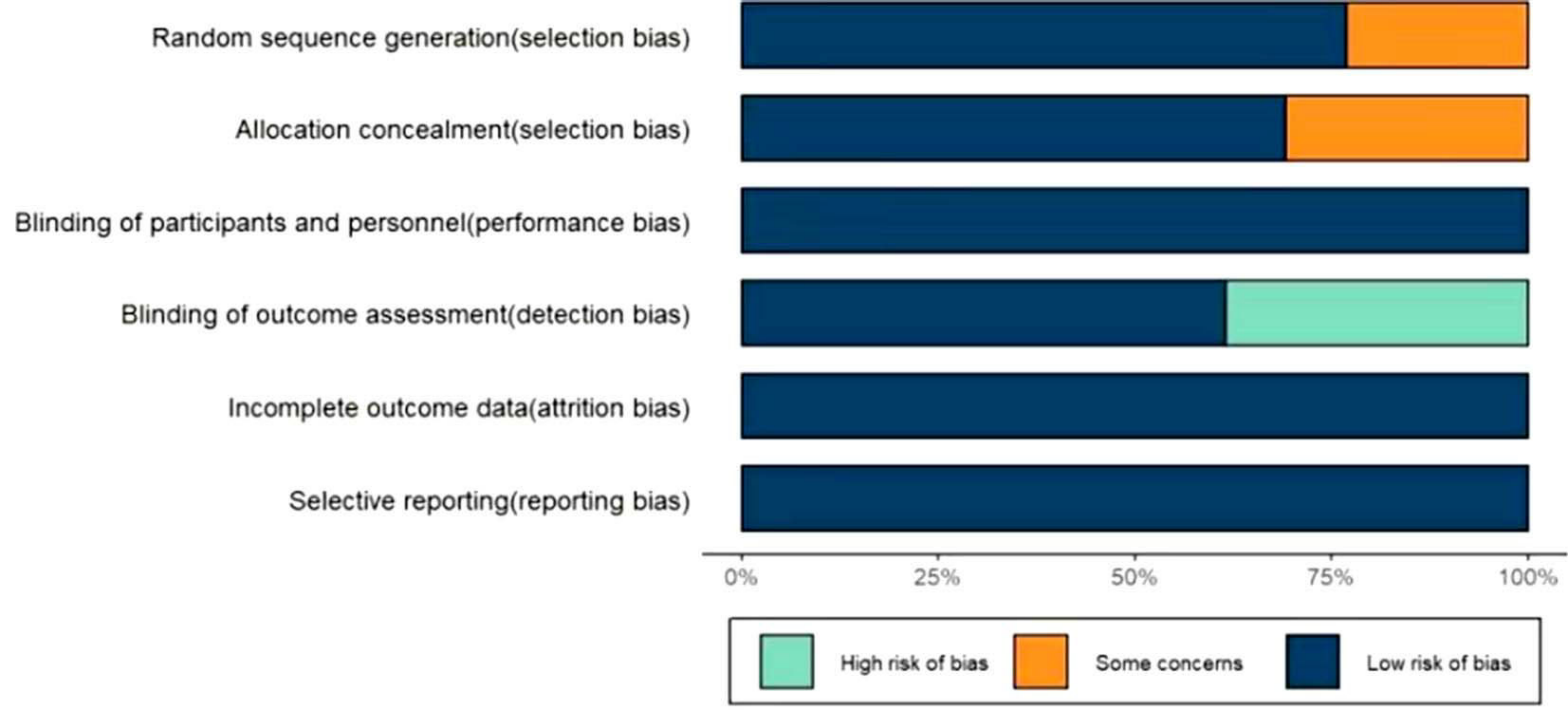
Risk of bias assessment of included studies.

Based on the Cochrane risk assessment tool, 3 (23%) studies did not mention the random sequence generation method and 4 (31%) did not mention the allocation concealment. All the studies were blinded to both participants and personnel. But 5 (38%) of them had a high risk of detection bias for lack of blinding on outcome assessment. The attrition bias and reporting bias were both low as no study was selectively reporting or with incomplete outcome data. Overall, the risks of bias were relatively low for the whole included trials (See **Figure 5**). No evidence of the funnel plot asymmetry was found (**Figures S35–41**). The Egger’s tests demonstrated no significant publication bias was revealed for the primary outcomes and most of the secondary outcomes (P values were all over 0·05).

## 4 Discussion

Several subcutaneous semaglutide randomized controlled trials have shown a great reduction on weight loss, blood glucose and blood pressure, as well as a low rate of adverse events, under different medication treatments. However, which treatment scheme is to be superior, safer, and more durable, especially under diverse and complex personal situations, rare research is reported. Two previous meta-analysis, including 4 and 7 studies respectively, evaluated the efficacy and safety of semaglutide focused on once-weekly treatment only (38, 39). None of them evaluated the quality of evidence for outcomes by the GRADE assessment. Therefore, in this meta-analysis, we explored the efficacy and safety of subcutaneous semaglutide systematically under the comprehensive different treatment conditions, to provide suggestions on medication interventions for adults with overweight or obese.

There were four main findings in our analysis as followed. Firstly, subcutaneous semaglutide showed significant weight loss and cardiometabolic benefits, as well as the improvements in the quality of life in the adults with overweight or obese. Secondly, subcutaneous semaglutide was relatively safe with the consideration of overall adverse events. Thirdly, consistent with the occurrence of side effects in the GLP-1 RAs, gastrointestinal disorders and hypoglycemia were the most common adverse events. Finally, the efficacy and safety of subcutaneous semaglutide varied depending on treatment dosage, administration frequency, trial duration, baseline obesity level and whether accompanied with lifestyle intervention.

Some sources of heterogeneity in this study remained unable to be detected. The likely causes might due to the followings. First of all, the pathogenesis of obesity was multi-factorial and not completely understood, like the genetic factors, different diet and exercise habits, induced by other diseases such as polycystic, et al. (40, 41) Since the mechanism of semaglutide in weight control was primarily appetite restriction and reduction of food intake, there might be some variations on its effectiveness among people with different causes of obesity (42, 43). On the other hand, the medications were administered in a variety of ways, such as the different kinds of starting dosage and methods of dose-escalation. Besides, some of our included trials involved participants with different comorbidities, like diabetes and non-alcoholic fatty liver disease, which might have an impact on treatment effect. Finally, the different sample sizes also had influence on the results.

One pharmacodynamic study reported that semaglutide demonstrated a dose-dependent effect on weight loss (44). In our study, subgroup analysis showed that the absolute weight change and waist circumference values decreased dramatically in the high-dose group than those in the low-dose group. However, the improvements in cardiovascular metabolic indexes, quality of life, and incidence rates of adverse events were identical in the two dosage groups.

Analysis with different treatment frequencies showed no obvious advantages on the reduction of most weight-related outcomes, except that patients in the once-daily group were more likely to achieve the goal of at least 5% weight loss. But in the meanwhile, the incidence rate of overall adverse reactions and hypoglycemia were higher in the once-daily group compared with once-weekly group. Research suggested the weekly administration frequency could enhance patients’ adherence. Indeed, for clinical convenience, frequency of once-weekly was supported by the pharmacokinetic models and drug tolerability trials (28, 30). For safety consideration, as well as for convenience and compliance in the practice, we recommend semaglutide treatment once a week for weight control.

For the analysis of trial duration, the absolute value of weight change was significantly higher in the long-term group than that the short-term group. But participants in the short-term group were more likely to achieve the goal of weight loss at least 5%. Notably, the incidence of total adverse events, withdrawal and vomiting were higher in the long-term group. The reasons to withdraw might be a quick and desirable achievement of weight loss goal after a short period of medication administration. On the other hand, the effect of weight loss in late stage might be not as satisfied as that in the early stage. Besides, adverse reactions were developed intolerable gradually in the late period of treatment and eventually, people ended up the intervention ahead of time. Under these circumstances, it is suggested that patients choose the appropriate length of treatment based on their weight loss results and tolerance of adverse effects.

Lifestyle interventions were the foundation of all obesity treatments according to most of the recommendations and guidelines (45). Although those participants without lifestyle intervention achieved more on weight loss goal of 5%, they had a higher incidence of hypoglycemia actually, compared with those with lifestyle modification. In terms of cardiovascular benefit, greater improvement in TG was seen in the group along with lifestyle intervention. So, we believe that diet and exercise modification can be helpful for stabilization of the blood glucose and serum lipids, especially accompanied with anti-obesity drug usage. Maintaining healthy lifestyle was not only beneficial for weight management, but also good for reducing hypoglycemia, optimizing lipid metabolism when applied with obesity treatment simultaneously. Therefore, we recommend lifestyle interventions in parallel with semaglutide administration.

Our review provided a systematic assessment of efficacy and safety of subcutaneous semaglutide in the treatment of adults with overweight or obese, especially compared the effects under various situations, like different baseline BMI, administration dosages, delivery frequencies, trial duration, with or without lifestyle interventions, hoping to give useful information to clinical practice on pharmacological purpose. The results showed that subcutaneous semaglutide treatment had great beneficial effect on body weight control, with additional improvements on cardiovascular and metabolic profiles. Based on subgroup analysis, we recommend semaglutide to be coupled with lifestyle interventions, and be administered subcutaneously with target dosage of 2·0 mg or more, once weekly. There were some limitations in this study. Firstly, we only compared semaglutide with placebo, no pairwise comparison with other approved anti-obesity drugs. On the other hand, although subgroup analysis and meta-regressions were performed, part of the sources of heterogeneity could not yet be fully identified.

For better medication application on anti-obesity, we suggest in the future, large sample size, more varieties of ethnic, multi-center collaboration with high-quality randomized controlled trials to be conducted. Prolonged follow-up observations after treatment are also recommended to assess the long-term effect and safety on weight loss, as well as the evaluation of cardiometabolic benefits.

## Data availability statement

The original contributions presented in the study are included in the article/**Supplementary Material**. Further inquiries can be directed to the corresponding authors.

## Author contributions

RZ and Q-CH are joint first authors. PS, JT, and Z-WW conceived the study idea and designed the search strategy. RZ, Q-CH, X-LL, and YiL screened studies and collected the data. L-LY, Y-MY, and Z-WW assessed the risk of bias. RZ, Q-CH, and B-HL analyzed the data. RZ, Q-CH, and PS wrote the first drafts of the figures, tables, and appendices. RZ, Q-CH, and PS wrote the first draft of the manuscript and interpreted the data analysis. YuL, LW, T-XL, LD, JT, Z-WW, and X-QY critically revised the manuscript. RZ, Q-CH, and PS are the guarantors. The corresponding author attests that all listed authors meet authorship criteria and that no others meeting the criteria have been omitted. All authors contributed to the article and approved the submitted version.
