# Supplementary material for: Efficacy and safety of subcutaneous semaglutide in adults with overweight or obese: a subgroup meta-analysis of randomized controlled trials
Source: Front Endocrinol (Lausanne). 2023 Jun 26;14:1132004. doi: 10.3389/fendo.2023.1132004 (PMC10338217; doi:10.3389/fendo.2023.1132004)
Supplement: Supplementary file 1 [file DataSheet_1.zip › Data Sheet 1/supplementary/Research in Context.pdf]

Research in context

Evidence before this study

Semaglutide was initially developed for the treatment of type 2 diabetes, and later proved to have a significant effect on weight loss as well as low incidence of adverse events.
Semaglutide, a novel, long-acting glucagon-like peptide-1 receptor agonists, plays a role in weight loss mainly by increasing satiety and delaying gastric emptying to reduce appetite and limit energy intake. Up to now, it is unclear which methods will obtain superior therapeutic effect of semaglutide under the different administration situations.

Added value of this study

The efficacy and safety of subcutaneous semaglutide under the diverse circumstances, such as different dosages, administration frequencies, trial durations, baseline obesity classifications and whether accompanied with lifestyle interventions, were comprehensively evaluated. Semaglutide treatment may display a better effect when to be coupled with lifestyle interventions, and target dose of 2·0 mg or more subcutaneously once weekly. Most of our results were estimated based on moderate and high levels of evidence and the information will be provided helpful information to pharmacological decision making.

Implications of all the available evidence

In clinical practice, comprehensive considerations with the occurrence of adverse events, weight loss efficacy and patient's individual conditions, will be beneficial to healthcare practitioners, patients and policy makers. Our results will provide useful information to clinical practice for pharmacological purpose on treatment of obesity.
