# Supplementary material for: Efficacy and safety of subcutaneous semaglutide in adults with overweight or obese: a subgroup meta-analysis of randomized controlled trials
Source: Front Endocrinol (Lausanne). 2023 Jun 26;14:1132004. doi: 10.3389/fendo.2023.1132004 (PMC10338217; doi:10.3389/fendo.2023.1132004)
Supplement: Supplementary file 1 [file DataSheet_1.zip › Data Sheet 1/supplementary/Supplementary materials.pdf]

Supplementary Text 1 Search strategy for International and National Trial registries search results.

1. - World Health Organization-International Clinical Trials Registry Platform (**http//apps.who.int /trialsearch/**) 6 records completed
2. - ClinicalTrials.gov (**https //www.clinicaltrials.gov/ct2/home**) 12 records completed
3. - European Union(EU) Clinical Trials Register (**https //www.clinicaltrialsregister.eu/**) 7 records completed
4. - Health Canada Clinical Trial Database (**https//www.canada.ca/en/health-canada/services/drugs-health-products/drug-products/health-canada-clinical-trials-database.html**) 0 records
5. - German Clinical Trials Register (**https //www.drks.de/drks_web/**) 0 records
6. - Netherlands Trial Register (Dutch) (**http //www.trialregister.nl/trialreg/index.asp**) 0 results
7. - Swiss National Clinical Trials Portal (**http//www.kofam.ch/en/swiss-clinical-trials-portal.html**) 1 records completed
8. - Australian New Zealand Clinical Trials Registry (**http //www.anzctr.org.au/**) 0 results
9. - Chinese Clinical Trial Register (**http //www.chictr.org.cn/enIndex.aspx**) 0 results
10. - Clinical Trials Registry–India (**http //ctri.nic.in/**) 0 records
11. - Japan Primary Registries Network (**https //jrct.niph.go.jp/search**) 0 records completed
12. - Clinical Research Information Service, Republic of Korea (**https//cris.nih.go.kr/cris/en/use_guide/cris_introduce.jsp**) 0 records

Supplementary Table 1 PRISMA Checklist

Section/topic	#	Checklist item	Reported on page #
**TITLE**			
Title	1	Identify the report as a systematic review, meta-analysis, or both.	1
**ABSTRACT**			
Structured summary	2	Provide a structured summary including, as applicable background; objectives; data sources; study eligibility criteria,participants, and interventions; study appraisal and synthesis methods; results; limitations; conclusions and implications of key findings; systematic review registration number.	1
**INTRODUCTION**			
Rationale	3	Describe the rationale for the review in the context of what is already known.	2, 3
Objectives	4	Provide an explicit statement of questions being addressed with reference to participants, interventions, comparisons, outcomes, and study design (PICOS).	2, 3
**METHODS**			
Protocol and registration	5	Indicate if a review protocol exists, if and where it can be accessed (e.g., Web address), and, if available, provide registration information including registration number.	3
Eligibility criteria	6	Specify study characteristics (e.g., PICOS, length of follow-up) and report characteristics (e.g., years considered, language, publication status) used as criteria for eligibility, giving rationale.	3
Information sources	7	Describe all information sources (e.g., databases with dates of coverage, contact with study authors to identify additional studies) in the search and date last searched.	3
Search	8	Present full electronic search strategy for at least one database, including any limits used, such that it could be repeated.	3
Study selection	9	State the process for selecting studies (i.e., screening, eligibility, included in systematic review, and, if applicable, included in the meta-analysis).	3

Data collection process	10	Describe method of data extraction from reports (e.g., piloted forms, independently, in duplicate) and any processes for obtaining and confirming data from investigators.	3
Data items	11	List and define all variables for which data were sought (e.g., PICOS, funding sources) and any assumptions and simplifications made.	3
Risk of bias in individual studies	12	Describe methods used for assessing risk of bias of individual studies (including specification of whether this was done at the study or outcome level), and how this information is to be used in any data synthesis.	2, 3
Summary measures	13	State the principal summary measures (e.g., risk ratio, difference in means).	4
Synthesis of results	14	Describe the methods of handling data and combining results of studies, if done, including measures of consistency (e.g., I^2) for each meta-analysis.	4
Risk of bias across studies	15	Specify any assessment of risk of bias that may affect the cumulative evidence (e.g., publication bias, selective reporting within studies).	4
Additional analyses	16	Describe methods of additional analyses (e.g., sensitivity or subgroup analyses,meta-regression), if done, indicating which were pre-specified.	4
**Section/topic**	**#**	**Checklist item**	**Reported on page #**
**RESULTS**			
Study selection	17	Give numbers of studies screened, assessed for eligibility, and included in the review, with reasons for exclusions at each stage, ideally with a flow diagram.	4
Study characteristics	18	For each study, present characteristics for which data were extracted (e.g., study size, PICOS, follow-up period) and provide the citations.	5
Risk of bias within studies	19	Present data on risk of bias of each study and, if available, any outcome level assessment (see item 12).	10
Results of individual studies	20	For all outcomes considered (benefits or harms), present, for each study (a) simple summary data for each intervention group (b) effect estimates and confidence intervals, ideally with a forest plot.	6-10
Synthesis of results	21	Present results of each meta-analysis done, including confidence intervals and measures of consistency.	6-10
Risk of bias across studies	22	Present results of any assessment of risk of bias across studies (see Item 15).	6-10
Additional analysis	23	Give results of additional analyses, if done (e.g., sensitivity or subgroup analyses, meta-regression [see Item 16]).	6-10
**DISCUSSION**			
Summary of evidence	24	Summarize the main findings including the strength of evidence for each main outcome; consider their relevance to key groups (e.g., healthcare providers, users, and policy makers).	11
Limitations	25	Discuss limitations at study and outcome level (e.g., risk of bias), and at review-level (e.g., incomplete retrieval of identified research, reporting bias).	12
Conclusions	26	Provide a general interpretation of the results in the context of other evidence, and implications for future research.	12
**FUNDING**			
Funding	27	Describe sources of funding for the systematic review and other support (e.g., supply of data); role of funders for the systematic review.	4

Supplementary Table 2 Search strategy for randomized controlled trials of the efficacy and safety of intravenous semaglutide in the overweight and obese people.

**Pubmed**		
Step	Search	Hits
#1	Obesity[MeSH]	244720
#2	Weight Loss[MeSH]	47282
#3	Overweight[MeSH]	255500
#4	((((((((((((((((obes*[Title/Abstract]) OR (body mass ind*[Title/Abstract])) OR (adiposity[Title/Abstract])) OR (overweight[Title/Abstract])) OR (over weight[Title/Abstract])) OR (overload syndrome*[Title/Abstract]))) OR (overfeed*[Title/Abstract])) OR (over eat*[Title/Abstract])) OR (over feed*[Title/Abstract])) OR (overfed[Title/Abstract])) OR (over fed[Title/Abstract])) OR (weight cycling[Title/Abstract])) OR (skinfold thickness[Title/Abstract])) OR (antiobesity[Title/Abstract])) OR (anti-obesity[Title/Abstract])) OR (obesitas[Title/Abstract])) OR (bodyweight[Title/Abstract])) OR (body weight[Title/Abstract])	714131
#5	#1 OR #2 OR #3 OR #4	771193
#6	Randomized Controlled Trial[Publication Type]	570737
#7	((((((((((randomized controlled study[Title/Abstract]) OR (randomized controlled trial[Title/Abstract])) OR (randomized study[Title/Abstract])) OR (randomized trial[Title/Abstract])) OR (randomized placebo-controlled study[Title/Abstract])) OR (randomized placebo-controlled trial[Title/Abstract])) OR (randomized placebo controlled[Title/Abstract])) OR (randomized placebo-controlled[Title/Abstract])) OR (randomized double-blin*[Title/Abstract])) OR (randomized double blin*[Title/Abstract])) OR ((randomized[Title/Abstract] AND double-blin*[Title/Abstract])) OR ((randomized[Title/Abstract] AND placebo-controlled[Title/Abstract]))	272248
#8	#6 OR #7	652861
#9	"semaglutide" [Supplementary Concept]	402

#10	((semaglutide[Title/Abstract]) OR (wegovy[Title/Abstract])) OR (Ozempic[Title/Abstract])	787
#11	#9 OR #10	826
#10	#5 AND #8 AND #11	71
**Embase AND Medline**		
Step	Search	Hits
#1	'obesity'/exp	607584
#2	'body weight loss'/exp	212508
#3	obes* ab, ti OR 'body mass ind*' ab, ti OR adiposity ab, ti OR overweight ab, ti OR 'over weight' ab, ti OR 'overload syndrome' ab, ti OR 'over eat*' ab, ti OR overfeed* ab, ti OR 'over feed*' ab, ti OR overfed ab, ti OR 'over fed' ab, ti OR 'weight cycling' ab, ti OR 'skinfold thickness' ab, ti OR antiobesity ab, ti OR 'anti obesity' ab, ti OR obesitas ab, ti OR bodyweight ab, ti OR 'body weight' ab, ti	1007409
#4	'semaglutide'/exp	2294
#5	semaglutide ab, ti OR ozempic ab, ti OR wegovy ab, ti	1322
#6	'randomized controlled trial'/exp	714365
#7	'randomized controlled study' ab, ti OR 'randomized controlled trial' ab, ti OR 'randomized study' ab, ti OR 'randomized trial' ab, ti OR 'randomized placebo-controlled study' ab, ti OR 'randomized placebo-controlled trial' ab, ti OR 'randomized placebo controlled' ab, ti OR 'randomized placebo-controlled' ab, ti OR 'randomized double-blin*' ab, ti OR 'randomized double blin*' ab, ti OR (randomized ab, ti AND 'double blin*' ab, ti) OR (randomized ab, ti AND 'placebo controlled' ab, ti)	375396
#8	#1 OR #2 OR #3	1277879
#9	#4 OR #5	2392
#10	#6 OR #7	838112
#11	#8 AND #9 AND #10	203
**Web of Science**		
Step	Search	Hits
#1	(((((((((((((((((AB=(obesity)) OR AB=(body weight loss)) OR AB=(obes*)) OR AB=(body mass ind*)) OR AB=(adiposity)) OR AB=(overweight)) OR AB=(over weight)) OR AB=(overload syndrome)) OR AB=(over eat*)) OR AB=(overfeed*)) OR AB=(over feed*)) OR AB=(overfed)) OR AB=(over fed)) OR AB=(weight cycling)) OR AB=(skinfold thickness)) OR AB=(antiobesity)) OR AB=(anti obesity)) OR AB=(obesitas)) OR AB=(bodyweight)) OR AB=(body weight)	1228384
#2	((AB=(semaglutide)) OR AB=(ozempic)) OR AB=(wegovy)	705
#3	(((((((((AB=(randomized controlled trial)) OR AB=(randomized controlled study)) OR AB=(randomized controlled trial)) OR AB=(randomized study)) OR AB=(randomized trial)) OR AB=(randomized placebo-controlled study)) OR AB=(randomized placebo-controlled trial)) OR AB=(randomized placebo controlled)) OR AB=(randomized placebo-controlled)) OR AB=(randomized double-blin*)) OR AB=(randomized double blin*)	717003
#4	#1 AND #2 AND #3	113
**Cochrane**		
Step	Search	Hits
#1	(obes*) ti, ab, kw OR (body mass ind*) ti, ab, kw OR (adiposity) ti, ab, kw OR (overweight) ti, ab, kw OR (over weight) ti, ab, kw OR (overload syndrome*) ti, ab, kw OR (over eat*) ti, ab, kw OR (overfeed*) ti, ab, kw OR (overfed) ti, ab, kw OR (over fed) ti, ab, kw (weight cycling) ti, ab, kw OR (skinfold thickness) ti, ab, kw OR (antiobesity) ti, ab, kw OR (anti-obesity) ti, ab, kw OR (obesitas) ti, ab, kw OR(bodyweight) ti, ab, kw OR (body weight) ti, ab, kw (Word variations have been searched)	150045
#2	MeSH descriptor [Obesity] this term only	12928
#3	MeSH descriptor [Weight Loss] this term only	6801
#4	MeSH descriptor [Overweight] this term only	5863
#5	#1 OR #2 OR #3 OR #4	150433
#6	(semaglutide) ti, ab, kw OR (Ozempic) ti, ab, kw OR (wegovy) ti, ab, kw	687
#7	MeSH descriptor [Randomized Controlled Trials as Topic] this term only	12787
#8	(Randomized Controlled Trial) pt (Word variations have been searched)	551850
#9	(randomized controlled study) ti, ab, kw OR (randomized controlled trial) ti, ab, kw OR (randomized study) ti, ab, kw OR (randomized trial) ti, ab, kw OR (randomized placebo-controlled study) ti, ab, kw OR (randomized placebo-controlled trial) ti, ab, kw OR (randomized placebo controlled) ti, ab, kw OR (randomized placebo-controlled) ti, ab, kw OR (randomized double-blin*) ti, ab, kw OR (randomized doubleblin*) ti, ab, kw	1099160
#10	#7 OR #8 OR #9	1222945
#11	#5 AND #6 AND #10	272

Supplementary Table 3 Subgroup analysis of the effect of absolute value of body weight change(kg)

	No of trials	n	WMD	95% CI		I^2(%)	$P_{heterogeneity}$	Tau-squared	$P_{between}$
**All studies**	18	5989	-8.97	-10.73	-7.21	99.19	<0.001	13.71	
**Degree of obesity**									0.02
Class I obesity	7	1062	-7.29	-8.34	-6.24	85.82	<0.001	1.44	
Class II obesity	11	4927	-9.83	-11.71	-7.94	98.75	<0.001	9.26	
**Trial duration**									<0.001
Long-term	11	5365	-10.00	-11.99	-8.00	96.92	<0.001	10.72	
Short-term	5	539	-6.72	-7.72	-5.72	83.45	<0.001	0.91	
**Weekly cumulative dose**									<0.001
High dose group	9	3941	-10.92	-12.38	-9.46	96.21	<0.001	3.99	
Low dose group	9	2048	-6.81	-7.88	-5.73	91.77	<0.001	2.15	
**Administration Frequency**									0.97
Daily	8	1459	-8.99	-10.74	-7.25	97.25	<0.001	5.54	
Weekly	10	4530	-8.92	-11.87	-5.98	98.93	<0.001	21.67	
**Lifestyle intervention**									0.67
With	7	4377	-9.47	-13.10	-5.84	98.70	<0.001	23.12	
Without	11	1612	-8.61	-10.06	-7.16	96.66	<0.001	5.23	

Supplementary Table 4 Subgroup analysis of the effect of percentage of body weight loss(%) change

	No of trials	n	WMD	95% CI		I^2(%)	$P_{heterogeneity}$	Tau-squared	$P_{between}$
**All studies**	11	5365	-10.00	-11.99	-8.00	96.92	<0.001	10.72	
**Degree of obesity**									0.71
Class I obesity	2	502	-9.35	-12.88	-5.83	83.26	0.01	5.40	
Class II obesity	9	4863	-10.14	-12.42	-7.87	97.47	<0.001	11.47	
**Trial duration**									-
Long-term	11	5365	-10.00	-11.99	-8.00	96.92	<0.001	10.72	
**Weekly cumulative dose**									0.09
High dose group	7	3879	-11.26	-13.30	-9.22	94.92	<0.001	6.81	
Low dose group	4	1486	-7.77	-11.22	-4.32	96.36	<0.001	11.85	
**Administration Frequency**									0.64
Daily	3	716	-10.55	-11.68	-9.43	62.37	0.07	0.62	
Weekly	8	4649	-9.81	-12.73	-6.89	97.80	<0.001	16.89	
**Lifestyle intervention**									0.64
With	8	4649	-9.81	-12.73	-6.89	97.80	<0.001	16.89	
Without	3	716	-10.55	-11.68	-9.43	62.37	0.07	0.62	

Supplementary Table 5 Subgroup analysis of the effect of body mass index change

	No of trials	n	WMD	95% CI		I^2(%)	$P_{heterogeneity}$	Tau-squared	$P_{between}$
**All studies**	9	3024	-3.19	-4.02	-2.37	94.89	<0.001	1.43	
**Degree of obesity**									0.59
Class I obesity	3	516	-2.96	-3.80	-2.13	69.83	0.04	0.36	
Class II obesity	6	2508	-3.33	-4.41	-2.26	96.20	<0.001	1.66	
**Trial duration**									0.43
Long-term	8	2999	-3.25	-4.13	-2.37	95.52	<0.001	1.48	
Short-term	1	25	-2.60	-3.95	-1.25	-	-	0	
**Weekly cumulative dose**									0.17
High dose group	4	1567	-3.90	-5.32	-2.48	95.44	<0.001	1.95	
Low dose group	5	1457	-2.63	-3.75	-1.51	92.52	<0.001	1.45	
**Administration Frequency**									0.09
Daily	3	716	-3.85	-4.51	-3.19	43.32	0.17	0.15	
Weekly	6	2308	-2.86	-3.80	-1.92	95.38	<0.001	1.23	
**Lifestyle intervention**									0.25
With	5	2283	-2.91	-3.93	-1.88	96.28	<0.001	1.27	

Without	4	741	-3.63	-4.33	-2.94	53.25	0.09	0.27	

Supplementary Table 6 Subgroup analysis of the effect of waist circumference change

	No of trials	n	WMD	95% CI		I^2(%)	$P_{heterogeneity}$	Tau-squared	$P_{between}$
**All studies**	13	5400	-7.21	-8.87	-5.56	96.03	<0.001	8.19	
**Degree of obesity**									0.19
Class I obesity	4	877	-6.00	-7.41	-4.59	84.22	<0.001	1.68	
Class II obesity	9	4523	-7.84	-10.21	-5.46	95.97	<0.001	11.67	
**Trial duration**									0.05
Long-term	10	4949	-7.55	-9.56	-5.55	95.71	<0.001	9.56	
Short-term	3	451	-5.32	-6.40	-4.23	56.16	0.10	0.45	
**Weekly cumulative dose**									0.01
High dose group	6	3557	-8.79	-10.71	-6.87	92.07	<0.001	4.86	
Low dose group	7	1843	-5.50	-6.89	-4.11	84.32	<0.001	2.55	
**Administration Frequency**									0.42
Daily	5	1102	-6.44	-7.76	-5.11	75.68	<0.001	1.51	
Weekly	8	4298	-7.57	-10.02	-5.13	96.65	<0.001	11.15	
**Lifestyle intervention**									0.58
With	7	4273	-7.41	-9.99	-4.84	97.12	<0.001	11.33	
Without	6	1127	-6.59	-7.90	-5.28	72.67	<0.001	1.58	

Supplementary Table 7 Subgroup analysis of the effect of weight loss goal of 5%

	No of trials	n	WMD	95% CI		I^2(%)	$P_{heterogeneity}$	Tau-squared	$P_{between}$
**All studies**	13	6152	3.00	2.53	3.56	83.29	<0.001	0.07	
**Degree of obesity**									<0.001
Class I obesity	4	877	4.58	3.38	6.21	44.95	0.14	0.04	
Class II obesity	9	5275	2.60	2.22	3.05	80.33	<0.001	0.04	
**Trial duration**									<0.001
Long-term	11	5766	2.73	2.34	3.19	79.60	<0.001	0.05	
Short-term	2	386	6.18	4.27	8.96	0	0.48	0	
**Weekly cumulative dose**									0.12
High dose group	7	4444	2.64	2.19	3.19	81.51	<0.001	0.05	
Low dose group	6	1708	3.69	2.54	5.37	58.97	<0.001	0.18	
**Administration Frequency**									<0.001
Daily	5	1102	4.14	3.26	5.26	48.11	0.10	0.03	
Weekly	8	5050	2.50	2.13	2.95	79.06	<0.001	0.04	
**Lifestyle intervention**									<0.001
With	8	5050	2.50	2.13	2.95	79.06	<0.001	0.04	
Without	5	1102	4.14	3.26	5.26	48.11	0.1	0.03	

Supplementary Table 8 Subgroup analysis of the effect of weight loss goal of 10%

	No of trials	n	WMD	95% CI		I^2(%)	$P_{heterogeneity}$	Tau-squared	$P_{between}$
**All studies**	11	5766	5.04	3.99	6.36	70.76	<0.001	0.10	
**Degree of obesity**									0.02
Class I obesity	2	491	10.12	5.46	18.77	0	0.56	0	
Class II obesity	9	5275	4.67	3.70	5.88	70.74	<0.001	0.08	
**Trial duration**									-
Long-term	11	5766	5.04	3.99	6.36	70.76	<0.001	0.10	
**Weekly cumulative dose**									0.89
High dose group	7	4334	5.08	3.71	6.96	79.50	<0.001	0.13	

Low dose group	4	1432	4.92	3.50	6.93	39.14	0.18	0.05	
**Administration Frequency**									0.4
Daily	3	716	5.80	4.29	7.83	0	0.93	0	
Weekly	6	5050	4.85	3.62	6.50	77.68	<0.001	0.12	
**Lifestyle intervention**									0.4
With	6	5050	4.85	3.62	6.50	77.68	<0.001	0.12	
Without	3	716	5.80	4.29	7.83	0	0.93	0	

Supplementary Table 9 Subgroup analysis of the effect of weight loss goal of 15%

	No of trials	n	WMD	95% CI		I^2(%)	$P_{heterogeneity}$	Tau-squared	$P_{between}$
**All studies**	11	5766	6.95	5.39	8.96	42.28	0.07	0.07	
**Degree of obesity**									0.29
Class I obesity	2	491	10.65	4.73	23.96	0	0.54	0	
Class II obesity	9	5275	6.69	5.09	8.80	49.25	0.05	0.08	
**Trial duration**									-
Long-term	11	5766	6.95	5.39	8.96	42.28	0.07	0.07	
**Weekly cumulative dose**									0.2
High dose group	7	4334	7.66	5.44	10.79	58.19	0.03	0.11	
Low dose group	4	1432	5.48	3.73	8.06	0	0.73	0	
**Administration Frequency**									0.84
Daily	3	716	6.71	4.30	10.46	0	0.82	0	
Weekly	8	5050	7.10	5.08	9.92	58.63	0.02	0.12	
**Lifestyle intervention**									0.84
With	8	5050	7.10	5.08	9.92	58.63	0.02	0.12	
Without	3	716	6.71	4.30	10.46	0	0.82	0	

Supplementary Table 10 Subgroup analysis of the effect of HbA1c change

	No of trials	n	WMD	95% CI		I^2(%)	$P_{heterogeneity}$	Tau-squared	$P_{between}$
**All studies**	15	3947	-0.82	-1.03	-0.60	99.06	<0.001	0.18	
**Degree of obesity**									0.01
Class I obesity	5	934	-1.26	-1.70	-0.81	97.14	<0.001	0.25	
Class II obesity	10	3013	-0.60	-0.84	-0.36	99.18	<0.001	0.15	
**Trial duration**									0.23
Long-term	12	3536	-0.70	-0.91	-0.48	98.95	<0.001	0.14	
Short-term	3	411	-1.28	-2.21	-0.35	99.26	<0.001	0.67	
**Weekly cumulative dose**									0.39
High dose group	7	1967	-0.70	-1.00	-0.40	98.59	<0.001	0.16	
Low dose group	8	1980	-0.92	-1.32	-0.52	99.30	<0.001	0.33	
**Administration Frequency**									0.29
Daily	8	1452	-0.91	-1.20	-0.61	99.40	<0.001	0.18	
Weekly	7	2495	-0.70	-0.94	-0.46	93.99	<0.001	0.10	
**Lifestyle intervention**									0.75
With	6	2470	-0.78	-1.05	-0.50	94.52	<0.001	0.11	
Without	9	1477	-0.84	-1.12	-0.56	99.35	<0.001	0.18	

Supplementary Table 11 Subgroup analysis of the effect of FPG change

	No of trials	n	WMD	95% CI		I^2(%)	$P_{heterogeneity}$	Tau-squared	$P_{between}$
**All studies**	12	3580	-1.18	-1.67	-0.69	98.67	<0.001	0.73	
**Degree of obesity**									0.92
Class I obesity	4	721	-1.14	-2.04	-0.25	98.40	<0.001	0.82	

Class II obesity	8	2859	-1.20	-1.81	-0.59	98.87	<0.001	0.77	
**Trial duration**									0.91
Long-term	9	3150	-1.20	-1.75	-0.65	98.72	<0.001	0.70	
Short-term	3	430	-1.12	-2.40	0.16	98.93	<0.001	1.26	
**Weekly cumulative dose**									0.40
High dose group	5	1653	-0.95	-1.38	-0.51	96.26	<0.001	0.24	
Low dose group	7	1927	-1.34	-2.15	-0.53	99.09	<0.001	1.18	
**Administration Frequency**									0.93
Daily	6	1027	-1.15	-2.04	-0.27	99.29	<0.001	1.22	
Weekly	6	2553	-1.20	-1.64	-0.76	96.00	<0.001	0.29	
**Lifestyle intervention**									0.61
With	5	2242	-1.32	-1.84	-0.79	96.36	<0.001	0.34	
Without	7	1338	-1.08	-1.84	-0.32	99.16	<0.001	1.03	

Supplementary Table 12 Subgroup analysis of the effect of TC change

	No of trials	n	WMD	95% CI		I^2(%)	$P_{heterogeneity}$	Tau-squared	$P_{between}$
**All studies**	5	952	-0.30	-0.52	-0.08	0	0.85	-	
**Degree of obesity**									-
Class II obesity	5	952	-0.30	-0.52	-0.08	0	0.85	-	
**Trial duration**									0.56
Long-term	4	927	-0.27	-0.51	-0.03	0	0.79	-	
Short-term	1	25	-0.44	-0.96	0.08	0	-	-	
**Weekly cumulative dose**									0.81
High dose group	2	449	-0.33	-0.68	0.01	0	0.37	-	
Low dose group	3	503	-0.28	-0.56	0.01	0	0.77	-	
**Administration Frequency**									0.25
Daily	3	716	-0.20	-0.48	0.07	0	1	-	
Weekly	2	236	-0.48	-0.84	-0.11	0	0.85	-	
**Lifestyle intervention**									0.38
With	1	211	-0.51	-1.03	0.01	100	-	-	
Without	4	741	-0.25	-0.50	-0.01	0	0.89	-	

Supplementary Table 13 Subgroup analysis of the effect of TG change

	No of trials	n	WMD	95% CI		I^2(%)	$P_{heterogeneity}$	Tau-squared	$P_{between}$
**All studies**	5	952	-0.63	-1.02	-0.25	8.05	0.36	-	
**Degree of obesity**									-
Class II obesity	5	952	-0.63	-1.02	-0.25	8.05	0.36	-	
**Trial duration**									0.32
Long-term	4	927	-0.76	-1.22	-0.31	10.46	0.34	-	
Short-term	1	25	-0.34	-1.03	0.35	0	-	-	
**Weekly cumulative dose**									0.09
High dose group	2	449	-0.95	-1.48	-0.43	23.52	0.25	-	
Low dose group	3	503	-0.28	-0.84	0.28	0	0.96	-	
**Administration Frequency**									0.19
Daily	3	716	-0.18	-0.96	0.59	0	1	-	
Weekly	2	236	-0.78	-1.22	-0.34	61.71	0.11	-	
**Lifestyle intervention**									0.04
With	1	211	-1.08	-1.65	-0.51	100	-	-	
Without	4	741	-0.27	-0.79	0.25	0	0.99	-	

Supplementary Table 14 Subgroup analysis of the effect of SBP change

	No of trials	n	WMD	95% CI		I^2(%)	$P_{heterogeneity}$	Tau-squared	$P_{between}$
**All studies**	12	5373	-4.82	-5.87	-3.77	62.43	<0.001	1.79	
**Degree of obesity**									0.69
Class I obesity	4	877	-5.23	-8.09	-2.36	82.91	<0.001	6.84	

Class II obesity	8	4496	-4.61	-5.64	-3.57	38.46	0.12	0.75	
**Trial duration**									0.92
Long-term	10	4987	-4.75	-5.58	-3.91	22.67	0.23	0.38	
Short-term	2	386	-5.01	-10.33	0.32	94.28	<0.001	13.90	
**Weekly cumulative dose**									0.69
High dose group	6	3556	-5.02	-6.04	-3.99	27.93	0.23	0.47	
Low dose group	6	1817	-4.54	-6.64	-2.44	77.14	<0.001	5.11	
**Administration Frequency**									1
Daily	5	1102	-4.78	-7.09	-2.47	7.61	<0.001	5.20	
Weekly	7	4271	-4.79	-5.98	-3.59	47.41	0.08	1.06	
**Lifestyle intervention**									1
With	7	4271	-4.79	-5.98	-3.59	47.41	0.08	1.06	
Without	5	1102	-4.78	-7.09	-2.47	7.61	<0.001	5.20	

Supplementary Table 15 Subgroup analysis of the effect of DBP change

	No of trials	n	WMD	95% CI		I^2(%)	$P_{heterogeneity}$	Tau-squared	$P_{between}$
**All studies**	12	5373	-2.09	-2.83	-1.34	69.94	<0.001	0.92	
**Degree of obesity**									0.54
Class I obesity	4	877	-2.43	-3.59	-1.27	46.37	0.13	0.63	
Class II obesity	8	4496	-1.95	-2.98	-0.92	72.54	<0.001	1.36	
**Trial duration**									0.87
Long-term	10	4987	-2.06	-2.94	-1.18	65.83	<0.001	1.10	
Short-term	2	386	-2.25	-4.28	-0.22	80.05	0.03	1.72	
**Weekly cumulative dose**									0.35
High dose group	6	3556	-2.47	-3.49	-1.45	61.01	0.03	0.85	
Low dose group	6	1817	-1.71	-2.91	-0.51	67.15	0.01	1.45	
**Administration Frequency**									0.96
Daily	5	1102	-2.16	-3.06	-1.27	28.11	0.23	0.29	
Weekly	7	4271	-2.13	-3.31	-0.95	76.75	<0.001	1.66	
**Lifestyle intervention**									0.96
With	7	4271	-2.13	-3.31	-0.95	76.75	<0.001	1.66	
Without	5	1102	-2.16	-3.06	-1.27	28.11	0.23	0.29	

Supplementary Table 16 Subgroup analysis of the effect of CRP change

	No of trials	n	WMD	95% CI		I^2(%)	$P_{heterogeneity}$	Tau-squared	$P_{between}$
**All studies**	4	927	-1.37	-1.82	-0.92	0	0.88	-	
**Degree of obesity**									-
Class II obesity	4	927	-1.37	-1.82	-0.92	0	0.88	-	
**Trial duration**									-
Long-term	4	927	-1.37	-1.82	-0.92	0	0.88	-	
**Weekly cumulative dose**									0.49
High dose group	2	449	-1.41	-1.88	-0.95	0	0.87	-	
Low dose group	2	478	-0.78	-2.51	0.95	0	0.70	-	
**Administration Frequency**									0.52
Daily	3	716	-0.93	-2.33	0.46	0	0.89	-	

Weekly	1	211	-1.42	-1.90	-0.94	0	-	-	
**Lifestyle intervention**									0.52
With	1	211	-1.42	-1.90	-0.94	0	-	-	
Without	3	716	-0.93	-2.33	0.46	0	0.89	-	

Supplementary Table 17 Subgroup analysis of the effect of SF-36v2 change

	No of trials	n	WMD	95% CI		I^2(%)	$P_{heterogeneity}$	Tau-squared	$P_{between}$
**All studies**	6	4300	1.22	0.69	1.74	63.85	0.02	0.24	
**Degree of obesity**									0.27
Class I obesity	2	491	0.77	-0.11	1.64	12.70	0.28	0.05	
Class II obesity	4	3809	1.37	0.76	1.98	70.38	0.02	0.24	
**Trial duration**									-
Long-term	6	4300	1.22	0.69	1.74	63.85	0.02	0.24	
**Weekly cumulative dose**									0.48
High dose group	4	3351	1.33	0.71	1.94	71.23	0.02	0.25	
Low dose group	2	949	0.97	-0.25	1.98	44.20	0.18	0.29	
**Administration Frequency**									-
Weekly	6	4300	1.22	0.69	1.74	63.85	0.02	0.24	
**Lifestyle intervention**									-
With	6	4300	1.22	0.69	1.74	63.85	0.02	0.24	

Supplementary Table 18 Subgroup analysis of the effect of IWQOL-Lite-CT change

	No of trials	n	WMD	95% CI		I^2(%)	$P_{heterogeneity}$	Tau-squared	$P_{between}$
**All studies**	5	3756	4.81	1.28	8.34	89.13	<0.001	13.84	
**Degree of obesity**									0.20
Class I obesity	2	491	2.78	0.13	5.43	0	0.62	0	
Class II obesity	3	3265	6.06	1.86	10.26	90.21	<0.001	12.24	
**Trial duration**									-
Long-term	5	3756	4.81	1.28	8.34	89.13	<0.001	13.84	
**Weekly cumulative dose**									0.19
High dose group	3	2807	6.10	1.94	10.27	88.80	<0.001	11.76	
Low dose group	2	949	2.89	0.46	5.33	0	0.59	0	
**Administration Frequency**									-
Weekly	5	3756	4.81	1.28	8.34	89.13	<0.001	13.84	
**Lifestyle intervention**									-
With	5	3756	4.81	1.28	8.34	89.13	<0.001	13.84	

Supplementary Table 19 Subgroup analysis of hypoglycemia under different situations

	No of trials	n	RR	95% CI		I^2(%)	$P_{heterogeneity}$	Tau-squared	$P_{between}$
**All studies**	11	5667	2.12	1.50	2.98	0	0.69	-	
**Degree of obesity**									0.38
Class I obesity	3	559	0.78	0.08	7.47	0	0.97	-	
Class II obesity	8	5108	2.17	1.53	3.07	0	0.48	-	
**Trial duration**									0.68
Long-term	10	5609	2.13	1.51	3.01	0	0.61	-	
Short-term	1	58	0.94	0.02	45.62	100.00	-	-	
**Weekly cumulative dose**									0.45
High dose group	6	4126	1.85	1.14	3.01	0	0.44	-	
Low dose group	5	1541	2.42	1.49	3.93	0	0.74	-	
**Administration Frequency**									0.03
Daily	3	716	3.53	1.99	6.27	0	0.99	-	

Weekly	8	4951	1.59	1.03	2.44	0	0.92	-	
**Lifestyle intervention**									0.04
With	7	4893	1.60	1.04	2.46	0	0.86	-	
Without	4	774	3.43	1.94	6.06	0	0.93	-	

Supplementary Table 20 Subgroup analysis of total adverse reactions under different situations

	No of trials	n	RR	95% CI		I^2(%)	$P_{heterogeneity}$	Tau-squared	$P_{between}$
**All studies**	17	6756	1.08	1.04	1.12	69.23	<0.001	0	
**Degree of obesity**									0.08
Class I obesity	6	1025	1.03	0.97	1.10	0	0.57	0	
Class II obesity	11	5731	1.10	1.05	1.15	78.94	<0.001	0	
**Trial duration**									0.03
Long-term	14	6298	1.10	1.05	1.14	72.79	<0.001	0	
Short-term	3	458	0.97	0.87	1.07	0	0.50	0	
**Weekly cumulative dose**									0.47
High dose group	10	4759	1.07	1.02	1.13	77.32	<0.001	0	
Low dose group	7	1997	1.10	1.05	1.15	16.01	0.31	0	
**Administration Frequency**									0.01
Daily	8	1487	1.14	1.10	1.19	0	0.43	0	
Weekly	9	5269	1.05	1.01	1.10	74.01	<0.001	0	
**Lifestyle intervention**									0.17
With	8	5197	1.06	1.02	1.11	71.58	<0.001	0	
Without	9	1559	1.11	1.05	1.18	43.57	0.08	0	

Supplementary Table 21 Subgroup analysis of adverse events leading to discontinuation under different situations

	No of trials	n	RR	95% CI		I^2(%)	$P_{heterogeneity}$	Tau-squared	$P_{between}$
**All studies**	18	6814	1.64	1.30	2.06	3.89	0.41	-	
**Degree of obesity**									0.08
Class I obesity	7	1083	1.02	0.57	1.83	0	0.66	-	
Class II obesity	11	5731	1.79	1.39	2.31	5.28	0.39	-	
**Trial duration**									0.02
Long-term	14	5298	1.82	1.42	2.33	0	0.61	-	
Short-term	4	516	0.83	0.44	1.57	0	0.64	-	
**Weekly cumulative dose**									0.17
High dose group	10	4729	1.85	1.38	2.47	16.30	0.29	-	
Low dose group	8	2085	1.32	0.90	1.94	0	0.65	-	
**Administration Frequency**									0.53
Daily	8	1487	1.46	0.96	2.22	29.29	0.19	-	
Weekly	10	5327	1.72	1.30	2.27	0	0.60	-	
**Lifestyle intervention**									0.51
With	8	5197	1.73	1.30	2.29	0	0.55	-	
Without	10	1617	1.46	0.97	2.20	20.46	0.25	-	

Supplementary Table 22 Subgroup analysis of vomiting under different situations

	No of trials	n	RR	95% CI		I^2(%)	$P_{heterogeneity}$	Tau-squared	$P_{between}$
**All studies**	17	6756	3.79	2.77	5.19	57.01	<0.001	0.20	
**Degree of obesity**									0.28
Class I obesity	6	1025	2.56	1.02	6.43	61.53	0.02	0.74	
Class II obesity	11	5731	4.43	3.36	5.55	30.47	0.16	0.05	
**Trial duration**									0.02
Long-term	14	6298	4.30	3.45	5.37	16.73	0.27	0.03	
Short-term	3	458	1.26	0.48	3.32	52.51	0.12	0.37	

**Weekly cumulative dose**									0.24
High dose group	10	4729	4.39	3.28	5.88	31.13	0.16	0.06	
Low dose group	7	2027	2.81	1.42	5.56	71.57	<0.001	0.58	
**Administration Frequency**									0.31
Daily	8	1487	2.99	1.52	5.88	66.27	<0.001	0.59	
Weekly	9	5269	4.39	3.30	5.82	33.76	0.15	0.06	
**Lifestyle intervention**									0.39
With	8	5197	4.36	3.24	5.86	39.9	0.11	0.06	
Without	9	1559	3.18	1.67	6.08	63.47	0.01	0.57	

Supplementary Table 23 Subgroup analysis of serious adverse reactions under different situations

	No of trials	n	RR	95% CI		I^2(%)	$P_{heterogeneity}$	Tau-squared	$P_{between}$
**All studies**	18	6814	1.16	0.97	1.38	8.38	0.35	-	
**Degree of obesity**									0.42
Class I obesity	7	1083	0.94	0.55	1.60	0	1	-	
Class II obesity	11	5731	1.18	0.98	1.43	42.37	0.07	-	
**Trial duration**									0.79
Long-term	14	6298	1.16	0.97	1.39	29.65	0.14	-	
Short-term	4	516	1.00	0.35	2.85	0	1	-	
**Weekly cumulative dose**									0.08
High dose group	10	4729	1.28	1.04	1.57	19.64	0.26	-	
Low dose group	8	2085	0.91	0.65	1.26	0	0.74	-	
**Administration Frequency**									0.95
Daily	8	1487	1.14	0.77	1.70	0	0.63	-	
Weekly	10	5327	1.16	0.95	1.41	32.43	0.15	-	
**Lifestyle intervention**									0.93
With	8	5197	1.16	0.95	1.41	47.35	0.07	-	
Without	10	1617	1.14	0.77	1.68	0	0.81	-	

Supplementary Table 24 Subgroup analysis of nausea under different situations

	No of trials	n	RR	95% CI		I^2(%)	$P_{heterogeneity}$	Tau-squared	$P_{between}$
**All studies**	17	6756	2.82	2.56	3.11	0	0.68	-	
**Degree of obesity**									0.05
Class I obesity	6	1023	4.08	2.77	6.02	0	0.92	-	
Class II obesity	11	5733	2.75	2.49	3.04	0	0.65	-	
**Trial duration**									0.15
Long-term	14	6298	2.78	2.52	3.07	0	0.73	-	
Short-term	3	458	4.04	2.45	6.66	0	0.53	-	
**Weekly cumulative dose**									0.29
High dose group	10	4732	2.74	2.45	3.06	0	0.75	-	
Low dose group	7	2024	3.10	2.54	3.78	0	0.43	-	
**Administration Frequency**									0.78
Daily	8	1487	2.90	2.36	3.55	0	0.64	-	
Weekly	9	5269	2.80	2.51	3.13	0	0.47	-	
**Lifestyle intervention**									0.78
With	8	5197	2.80	2.50	3.13	8.70	0.36	-	
Without	9	1559	2.89	2.38	3.52	0	0.74	-	

Supplementary Table 25 Subgroup analysis of constipation under different situations

	No of trials	n	RR	95% CI		I^2(%)	$P_{heterogeneity}$	Tau-squared	$P_{between}$
**All studies**	16	6684	2.66	2.11	3.36	55.60	<0.001	0.10	
**Degree of obesity**									0.04
Class I obesity	5	953	4.63	2.65	8.09	0	0.58	0	
Class II obesity	11	5731	2.42	1.91	3.07	59.33	0.01	0.08	
**Trial duration**									0.95
Long-term	2	6298	2.68	2.09	3.43	61.30	<0.001	0.11	
Short-term	14	386	2.77	1.14	6.72	0	0.87	0	
**Weekly cumulative dose**									0.46
High dose group	9	4657	2.51	1.87	3.37	66.57	<0.001	0.11	
Low dose group	7	2027	2.99	2.09	4.27	19.41	0.28	0.04	
**Administration Frequency**									0.38
Daily	8	1487	3.05	2.11	4.41	20.73	0.27	0.06	
Weekly	8	5197	2.48	1.85	3.31	68.19	<0.001	0.10	
**Lifestyle intervention**									0.38
With	8	5197	2.48	1.85	3.31	68.19	<0.001	0.10	
Without	8	1487	3.05	2.11	4.41	20.73	0.27	0.06	

Supplementary Table 26 Subgroup analysis of loss of appetite under different situations

	No of trials	n	RR	95% CI		I^2(%)	$P_{heterogeneity}$	Tau-squared	$P_{between}$
**All studies**	9	1688	2.25	1.46	3.46	43.18	0.08	0.18	
**Degree of obesity**									0.64
Class I obesity	2	138	2.64	1.21	5.74	38.91	0.20	0.14	
Class II obesity	7	1550	2.10	1.19	3.69	51.28	0.06	0.30	
**Trial duration**									0.73
Long-term	8	1616	2.30	1.36	3.89	49.71	0.05	0.28	
Short-term	1	72	2.00	1.10	3.65	-	-	0	
**Weekly cumulative dose**									0.35
High dose group	6	1052	2.55	1.77	3.67	0	0.50	0	
Low dose group	3	636	1.39	0.40	4.75	73.49	0.02	0.87	
**Administration Frequency**									0.77
Daily	6	1101	2.08	1.03	4.21	62	0.02	0.48	
Weekly	3	587	2.37	1.49	3.76	0	0.68	0	
**Lifestyle intervention**									0.42
With	2	515	3.02	1.46	6.22	0	0.82	0	
Without	7	1173	2.07	1.20	3.56	54.46	0.04	0.28	

Supplementary Table 27 Subgroup analysis of diarrhea under different situations

	No of trials	n	RR	95% CI		I^2(%)	$P_{heterogeneity}$	Tau-squared	$P_{between}$
**All studies**	17	6756	1.90	1.65	2.19	33	0.09	0.03	
**Degree of obesity**									0.33
Class I obesity	6	1025	2.24	1.55	3.23	20.82	0.28	0.04	
Class II obesity	11	5731	1.84	1.58	2.14	39.15	0.09	0.02	
**Trial duration**									0.54
Long-term	14	6298	1.87	1.61	2.18	37.07	0.08	0.03	
Short-term	3	458	2.22	1.31	3.76	26.68	0.26	0.06	
**Weekly cumulative dose**									0.2
High dose group	10	4729	1.78	1.47	2.15	47.24	0.05	0.04	
Low dose group	7	2027	2.13	1.74	2.62	0	0.57	0	
**Administration Frequency**									0.2
Daily	8	1487	2.17	1.71	2.74	11.21	0.34	0.01	

Weekly	9	5269	1.79	1.51	2.13	41.51	0.09	0.03	
**Lifestyle intervention**									0.1
With	8	5197	1.75	1.49	2.06	37.30	0.13	0.02	
Without	9	1559	2.23	1.76	2.82	13.37	0.32	0.02	

Supplementary Table 28 Subgroup analysis of cardiovascular disorders under different situations

	No of trials	n	RR	95% CI		I^2(%)	$P_{heterogeneity}$	Tau-squared	$P_{between}$
**All studies**	7	4893	0.82	0.66	1.02	0	0.80	-	
**Degree of obesity**									0.72
Class I obesity	2	501	1.30	0.11	15.30	0	0.87	-	
Class II obesity	5	4392	0.82	0.65	1.02	0	0.58	-	
**Trial duration**									-
Long-term	7	4893	0.82	0.66	1.02	0	0.80	-	
**Weekly cumulative dose**									0.49
High dose group	5	3888	0.81	0.64	1.01	0	0.64	-	
Low dose group	2	1005	1.21	0.39	3.74	0	0.92	-	
**Administration Frequency**									-
Weekly	7	4893	0.82	0.66	1.02	0	0.80	-	
**Lifestyle intervention**									-
With	7	4893	0.82	0.66	1.02	0	0.80	-	

Supplementary Table 29 Subgroup analysis of psychic disorders under different situations

	No of trials	n	RR	95% CI		I^2(%)	$P_{heterogeneity}$	Tau-squared	$P_{between}$
**All studies**	9	5212	1.06	0.77	1.46	44.28	0.07	0.09	
**Degree of obesity**									0.12
Class I obesity	2	501	3.49	0.77	15.83	0	0.85	0	
Class II obesity	7	4711	1.01	0.74	1.38	47.55	0.08	0.08	
**Trial duration**									-
Long-term	9	5212	1.06	0.77	1.46	44.28	0.07	0.09	
**Weekly cumulative dose**									0.57
High dose group	6	4049	1.01	0.70	1.44	48.83	0.08	0.08	
Low dose group	3	1163	1.28	0.60	2.73	32.69	0.23	0.16	
**Administration Frequency**									0.43
Daily	2	319	0.82	0.41	1.66	0	0.55	0	
Weekly	7	4893	1.13	0.77	1.67	56.58	0.03	0.12	
**Lifestyle intervention**									-
With	7	4893	1.13	0.77	1.67	56.58	0.03	0.12	
Without	2	319	0.82	0.41	1.66	0	0.55	0	

Supplementary Table 30 Subgroup analysis of allergic reactions under different situations

	No of trials	n	RR	95% CI		I^2(%)	$P_{heterogeneity}$	Tau-squared	$P_{between}$
**All studies**	10	5609	0.97	0.80	1.18	0	0.73	-	
**Degree of obesity**									0.63
Class I obesity	2	501	1.11	0.63	1.95	0	0.73	-	
Class II obesity	8	5108	0.96	0.78	1.18	0	0.57	-	
**Trial duration**									-
Long-term	10	5609	0.97	0.80	1.18	0	0.73	-	
**Weekly cumulative dose**									0.76
High dose group	6	4126	0.96	0.77	1.19	0	0.66	-	

Low dose group	4	1483	1.03	0.68	1.55	0	0.44	-	
**Administration Frequency**									0.55
Daily	3	716	0.82	0.46	1.46	0	0.43	-	
Weekly	7	4893	0.99	0.81	1.22	0	0.67	-	
**Lifestyle intervention**									0.55
With	7	4893	0.99	0.81	1.22	0	0.67	-	
Without	3	716	0.82	0.46	1.46	0	0.43	-	

Supplementary Table 31 Subgroup analysis of injection-site reactions under different situations

	No of trials	n	RR	95% CI		I^2(%)	$P_{heterogeneity}$	Tau-squared	$P_{between}$
**All studies**	10	5609	0.81	0.63	1.04	0	0.99	-	
**Degree of obesity**									0.31
Class I obesity	2	501	2.67	0.26	27.64	0	0.54	-	
Class II obesity	8	5108	0.80	0.62	1.03	0	0.65	-	
**Trial duration**									-
Long-term	10	5609	0.81	0.63	1.04	0	0.99	-	
**Weekly cumulative dose**									0.54
High dose group	6	4126	0.84	0.63	1.11	15.96	0.31	-	
Low dose group	4	1483	0.69	0.39	1.22	0	0.98	-	
**Administration Frequency**									0.86
Daily	3	716	0.84	0.48	1.47	0	0.78	-	
Weekly	7	4893	0.80	0.60	1.06	0	0.42	-	
**Lifestyle intervention**									0.86
With	7	4893	0.80	0.60	1.06	0	0.42	-	
Without	3	716	0.84	0.48	1.47	0	0.78	-	

Supplementary Table 32 GRADE assessment for the Certainty of Evidence for semaglutide compared to placebo for quantitative measures of weight related indicators and non-weight effect outcomes.

semaglutide for overweight or obesity

Patient or population patients with overweight or obesity
Settings DOE
Intervention semaglutide

**Outcomes**	**Illustrative comparative risks* (95% CI)**<br>Assumed risk<br>**Placebo**	 Corresponding risk<br>**semaglutide**	**Relative effect (95% CI)**	**No of Participants (studies)**	**Quality of the evidence (GRADE)**
**Body weight % reduction**<br>Electronic height and weight scales.<br>Follow-up 52 to 104 weeks	The mean body weight % reduction in the control groups was **-5.7 to -1.9 %**	The mean body weight % reduction in the intervention groups was **10 lower** (11.99 to 8 lower)		4889<br>(7 studies)	⊕⊕⊕⊝<br>**moderate**
**Body weight change**<br>Electronic height and weight scales.<br>Follow-up 12 to 104 weeks	The mean body weight in the control groups was<br>**-3.5 to 1.9 kg**	The mean body weight in the intervention groups was<br>**8.97 lower** (10.73 to 7.21 lower)		5036<br>(12 studies)	⊕⊕⊕⊝<br>**moderate**
**Waist circumference change**<br>Tape.<br>Follow-up 16 to 104 weeks	The mean waist circumference change in the control groups was<br>**-4.5 to 2.7 cm**	The mean waist circumference change in the intervention groups was<br>**7.21 lower** (8.87 to 5.56 lower)		4524<br>(8 studies)	⊕⊕⊕⊕<br>**high**
**Body Mass Index**<br>Electronic height and weight scales.<br>Follow-up 12 to 104 weeks	The mean body mass index in the control groups was<br>**-1.3 to 0.7 kg/m²**	The mean body mass index in the intervention groups was **3.19 lower** (4.02 to 2.37 lower)		2276<br>(5 studies)	⊕⊕⊕⊝<br>**moderate**
**Diastolic Blood Pressure**<br>Automatic electronic blood pressure monitor.<br>Follow-up 52 to 104 weeks	The mean diastolic blood pressure in the control groups was<br>**-2.2 to 0.7 mmHg**	The mean diastolic blood pressure in the intervention groups was **2.09 lower** (2.83 to 1.34 lower)		4496<br>(7 studies)	⊕⊕⊕⊕<br>**high**
**Systolic Blood Pressure**<br>Automatic electronic blood pressure monitor.<br>Follow-up 52 to 104 weeks	The mean systolic blood pressure in the control groups was<br>**-5.31 to 3.20 mmHg**	The mean systolic blood pressure in the intervention groups was **4.82 lower** (5.87 to 3.77 lower)		4496<br>(7 studies)	⊕⊕⊕⊕<br>**high**

**Total cholesterol**<br>Automatic biochemical analyzer.<br>Follow-up 16 to 68 months	The mean total cholesterol in the control groups was<br>**-0.33 to -0.10 mmol/L**	The mean total cholesterol in the intervention groups was **0.3 lower**<br>(0.52 to 0.08 lower)		680<br>(3 studies)	⊕⊕⊕⊕<br>**high**	
**Triglycerides change**<br>Automatic biochemical analyzer.<br>Follow-up 16to68 weeks	The mean triglycerides change in the control groups was<br>**-0.19 to 0.42 mmol/L**	The mean triglycerides change in the intervention groups was **0.63 lower**<br>(1.02 to 0.25 lower)		680<br>(3 studies)	⊕⊕⊕⊝<br>**moderate**	
**Fasting plasma glucose**<br>Automatic biochemical analyzer.<br>Follow-up 16 to104 weeks	The mean fasting plasma glucose in the control groups was<br>**-0.38 to 0.18 mmol/L**	The mean fasting plasma glucose in the intervention groups was **1.18 lower** (1.67 to 0.69 lower)		2707<br>(7 studies)	⊕⊕⊕⊝<br>**moderate**	
**Glycated hemoglobin change**<br>Automatic biochemical analyzer.<br>Follow-up 16 to104 weeks	The mean glycated hemoglobin change in the control groups was<br>**-0.40 to 0.08 %, mmol/mol**	The mean glycated hemoglobin change in the intervention groups was **0.82 lower** (1.03 to 0.6 lower)		2998<br>(9 studies)	⊕⊕⊕⊝<br>**moderate**	
**C-reactive protein**<br>Automatic biochemical analyzer.<br>Follow-up 52 to 68 weeks	The mean c-reactive protein in the control groups was<br>**-0.85 to -0.82 mg/L**	The mean c-reactive protein in the intervention groups was **1.37 lower**<br>(1.82 to 0.92 lower)		655<br>(2 studies)	⊕⊕⊕⊕<br>**high**	
**Impact of Weight on Quality ofLife-Lite Clinical Trials Version**<br>questionnaire.<br>Follow-up 68 to 68 weeks	The mean impact of weight on quality of life-lite clinical trials version in the control groups was<br>**0.84 to 5.30 score**	The mean impact of weight on quality of life-lite clinical trials version in the intervention groups was **4.81 higher**<br>(1.28 to 8.34 higher)		3282<br>(3 studies)	⊕⊕⊕⊝<br>**moderate**	
**Short Form 36v2 Health Survey, Acute Version**<br>questionnaire.<br>Follow-up 68 to 68 weeks	The mean short form 36v2 health survey, acute version in the control groups was<br>**-0.33 to 1.60 score**	The mean short form 36v2 health survey, acute version in the intervention groups was **1.22 higher**<br>(0.69 to 1.74 higher)		3826<br>(4 studies)	⊕⊕⊕⊕<br>**high**	

*The basis for the **assumed risk** (e.g. the median control group risk across studies) is provided in footnotes. The **corresponding risk** (and its 95% confidence interval) is based on the assumed risk in the comparison group and the **relative effect** of the intervention (and its 95% CI).
CI Confidence interval; **RR** Risk ratio;

GRADE Working Group grades of evidence
High quality Further research is very unlikely to change our confidence in the estimate of effect.
Moderate quality Further research is likely to have an important impact on our confidence in the estimate of effect and may change the estimate.

Supplementary Table 33 GRADE assessment for the Certainty of Evidence for semaglutide compared to placebo for binary variables of percentage of weight loss and incidence of adverse reactions.

semaglutide for overweight or obesity

Patient or population patients with overweight or obesity
Settings DOE
Intervention semaglutide

Outcomes	Illustrative comparative risks* (95% CI)		Relative effect (95% CI)	No of Participants (studies)	Quality of the evidence (GRADE)	Comments
	Assumed risk	Corresponding risk				
	**Placebo**	**semaglutide**				
**Body weight reduction 5% from baseline**<br>Electronic height and weight scales<br>Follow-up 26 to 104 weeks	**278 per 1000**	**835 per 1000**<br>(704 to 991)	**RR 3**<br>(2.53 to 3.56)	6152<br>(8 studies)	⊕⊕⊕⊝<br>**moderate**	
**Body weight reduction 10% from baseline**<br>Electronic height and weight scales<br>Follow-up 52 to 104 weeks	**114 per 1000**	**576 per 1000**<br>(456 to 726)	**RR 5.04**<br>(3.99 to 6.36)	5766<br>(7 studies)	⊕⊕⊕⊝<br>**moderate**	
**Body weight reduction 15% from baseline**<br>Electronic height and weight scales<br>Follow-up 52 to 104 weeks	**51 per 1000**	**355 per 1000**<br>(276 to 458)	**RR 6.95**<br>(5.39 to 8.96)	5757<br>(7 studies)	⊕⊕⊕⊕<br>**high**	
**Serious adverse events**<br>questionnaire<br>Follow-up 12 to 104 weeks	**73 per 1000**	**85 per 1000**<br>(71 to 101)	**RR 1.16**<br>(0.97 to 1.38)	6689<br>(12 studies)	⊕⊕⊕⊕<br>**high**	
**Adverse events**<br>questionnaire<br>Follow-up 52 to 104 weeks	**815 per 1000**	**880 per 1000**<br>(848 to 913)	**RR 1.08**<br>(1.04 to 1.12)	6756<br>(11 studies)	⊕⊕⊕⊕<br>**high**	
**Adverse events leading to discontinuation of drug or placebo**<br>questionnaire<br>Follow-up 12 to 104 weeks	**39 per 1000**	**65 per 1000**<br>(51 to 81)	**RR 1.64**<br>(1.3 to 2.06)	6814<br>(12 studies)	⊕⊕⊕⊕<br>**high**	
**Hypoglycemia**<br>questionnaire<br>Follow-up 12 to 68 weeks	**18 per 1000**	**39 per 1000**<br>(28 to 56)	**RR 2.13**<br>(1.53 to 2.98)	5667<br>(7 studies)	⊕⊕⊕⊕<br>**high**	
**Constipation**	**93 per 1000**	**248 per 1000**	**RR 2.66**	6372	⊕⊕⊕⊝	

questionnaire<br>Follow-up 16 to 104 weeks		(197 to 313)	(2.11 to 3.36)	(10 studies)	**moderate**
**Nausea**<br>questionnaire<br>Follow-up 20 to 104 weeks	**140 per 1000**	**395 per 1000**<br>(358 to 436)	**RR 2.82**<br>(2.56 to 3.11)	6644<br>(11 studies)	⊕⊕⊕⊕<br>**high**
**Diarrhea**<br>questionnaire<br>Follow-up 20 to 104 weeks	**142 per 1000**	**269 per 1000**<br>(234 to 311)	**RR 1.9**<br>(1.65 to 2.19)	6753<br>(11 studies)	⊕⊕⊕⊕<br>**high**
**Vomiting**<br>questionnaire<br>Follow-up 20 to 104 weeks	**51 per 1000**	**195 per 1000**<br>(142 to 267)	**RR 3.79**<br>(2.77 to 5.19)	6756<br>(11 studies)	⊕⊕⊕⊕<br>**high**
**Decreased appetite**<br>questionnaire<br>Follow-up 20 to 104 weeks	**62 per 1000**	**139 per 1000**<br>(90 to 214)	**RR 2.25**<br>(1.46 to 3.46)	1688<br>(6 studies)	⊕⊕⊕⊕<br>**high**
**Cardiovascular disorders**<br>questionnaire<br>Follow-up 68 to 68 weeks	**59 per 1000**	**49 per 1000**<br>(39 to 61)	**RR 0.82**<br>(0.66 to 1.02)	4893<br>(5 studies)	⊕⊕⊕⊝<br>**moderate**
**Allergic reaction**<br>questionnaire<br>Follow-up 52 to 68 weeks	**71 per 1000**	**69 per 1000**<br>(57 to 84)	**RR 0.97**<br>(0.8 to 1.18)	5609<br>(6 studies)	⊕⊕⊕⊝<br>**moderate**
**Psychiatric disorder**<br>questionnaire<br>Follow-up 68 to 72	**78 per 1000**	**82 per 1000**<br>(60 to 113)	**RR 1.06**<br>(0.77 to 1.46)	5212<br>(6 studies)	⊕⊕⊕⊝<br>**moderate**
**Injection-site reactions**<br>questionnaire<br>Follow-up 52 to 68 weeks	**47 per 1000**	**38 per 1000**<br>(30 to 49)	**RR 0.81**<br>(0.63 to 1.04)	5609<br>(6 studies)	⊕⊕⊕⊝<br>**moderate**

*The basis for the **assumed risk** (e.g. the median control group risk across studies) is provided in footnotes. The **corresponding risk** (and its 95% confidence interval) is based on the assumed risk in the comparison group and the **relative effect** of the intervention (and its 95% CI).

CI Confidence interval; **RR** Risk ratio;

GRADE Working Group grades of evidence

High quality Further research is very unlikely to change our confidence in the estimate of effect.

Moderate quality Further research is likely to have an important impact on our confidence in the estimate of effect and may change the estimate.

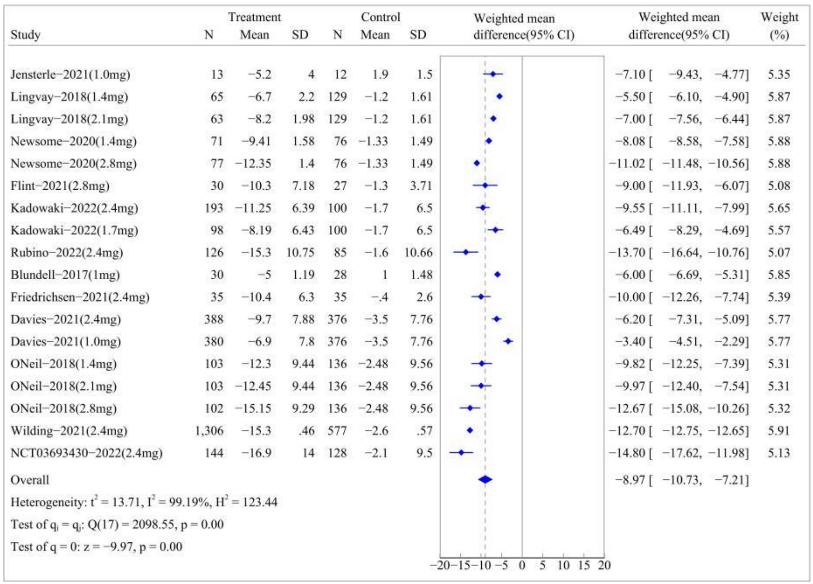

Supplementary Figure 1 Weighted mean difference of change in absolute value of weight change between the semaglutide and placebo control.

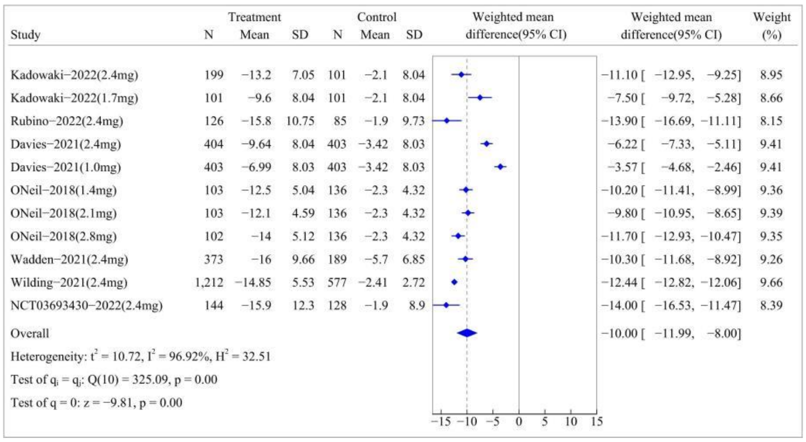

Supplementary Figure 2 Weighted mean difference of change in percentage of weight change between the semaglutide and placebo control.

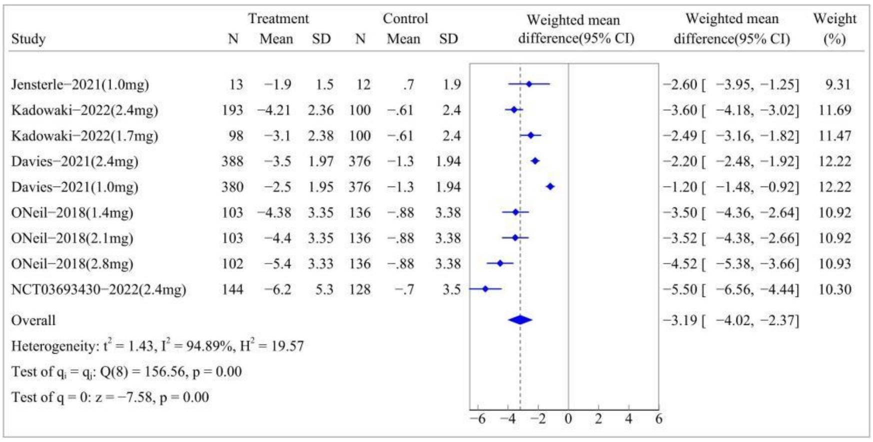

Supplementary Figure 3 Weighted mean difference of change in BMI between the semaglutide and placebo control.

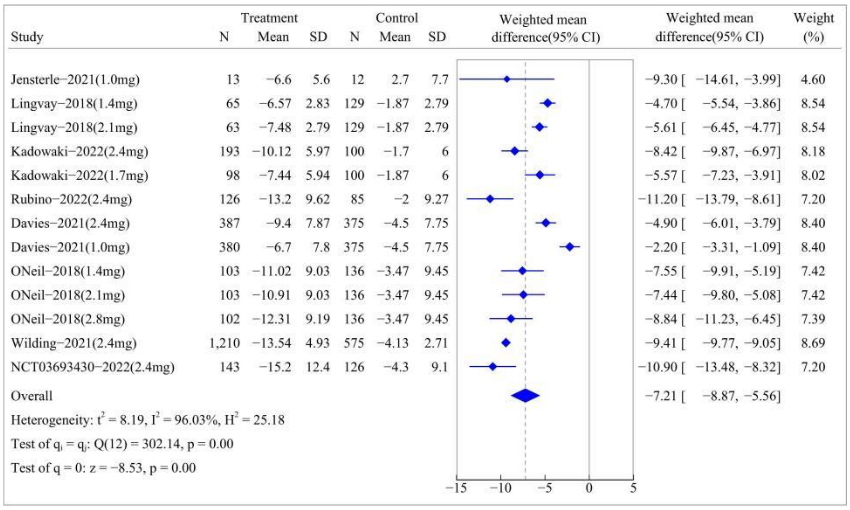

Supplementary Figure 4 Weighted mean difference in change in waist circumference (cm) between the semaglutide and placebo control

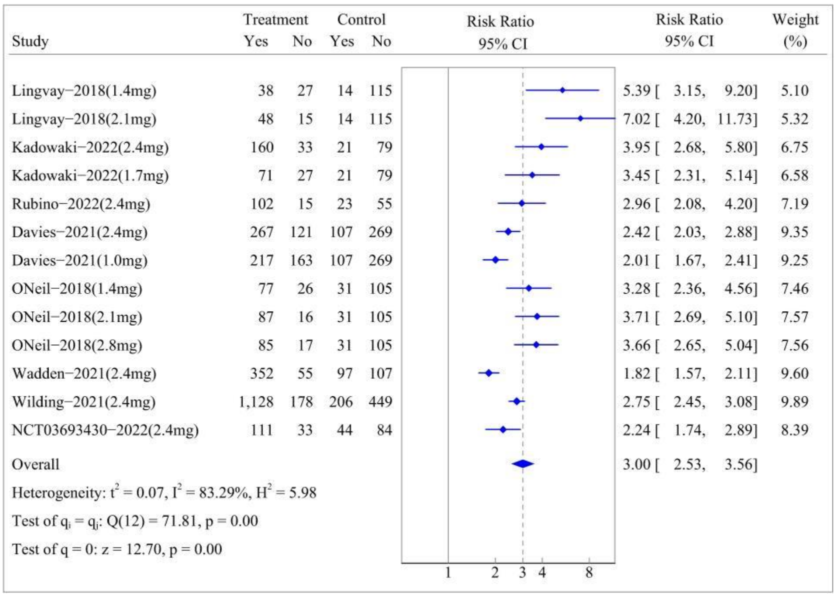

Supplementary Figure 5 Risk ratio in weight reduction of at least 5% between the semaglutide and placebo control

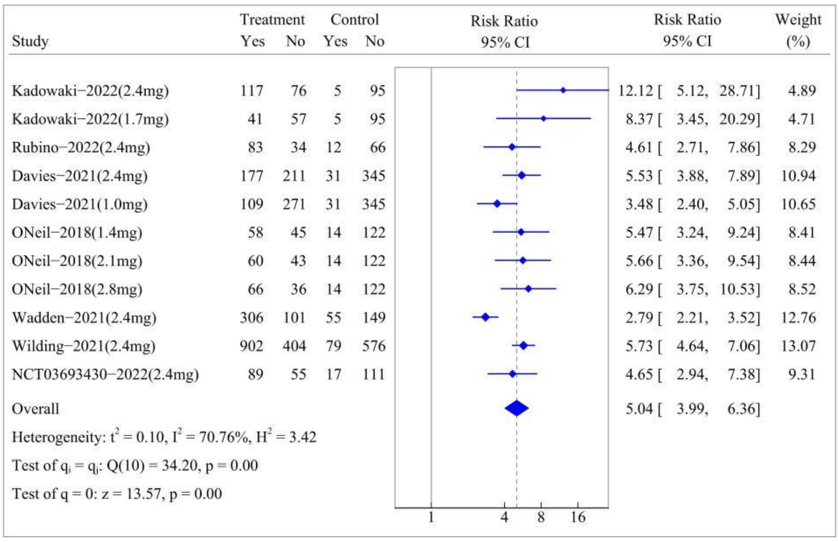

Supplementary Figure 6 Risk ratio in weight reduction of at least 10% between the semaglutide and placebo control

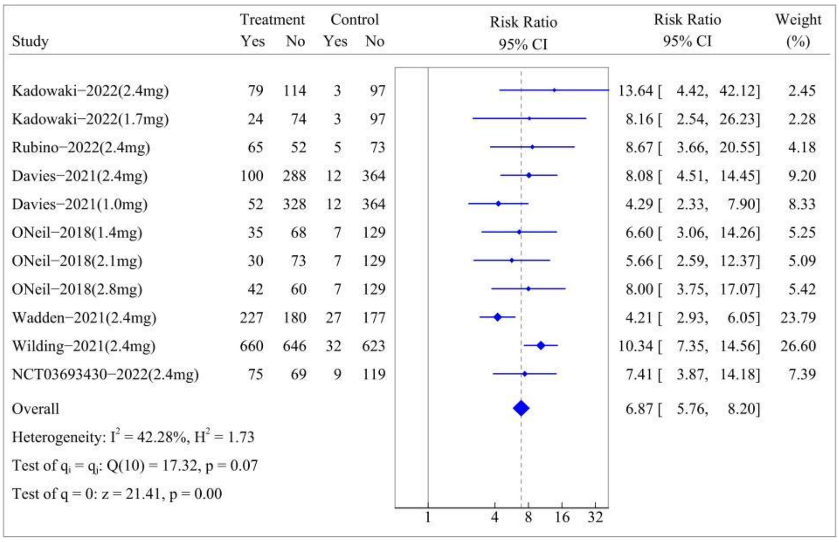

Supplementary Figure 7 Risk ratio in weight reduction of at least 15% between the semaglutide and placebo control

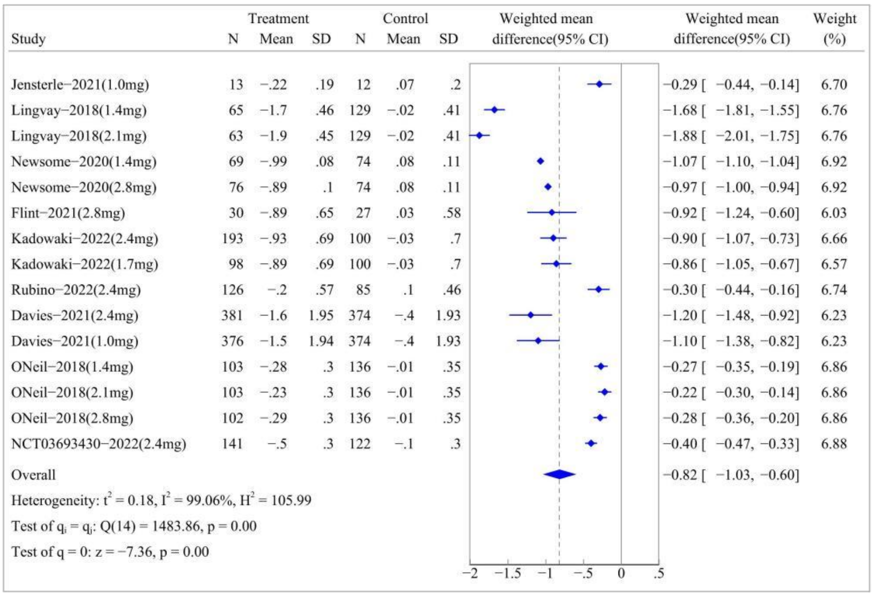

Supplementary Figure 8 Weighted mean difference in change in HbA1c between the semaglutide and placebo control

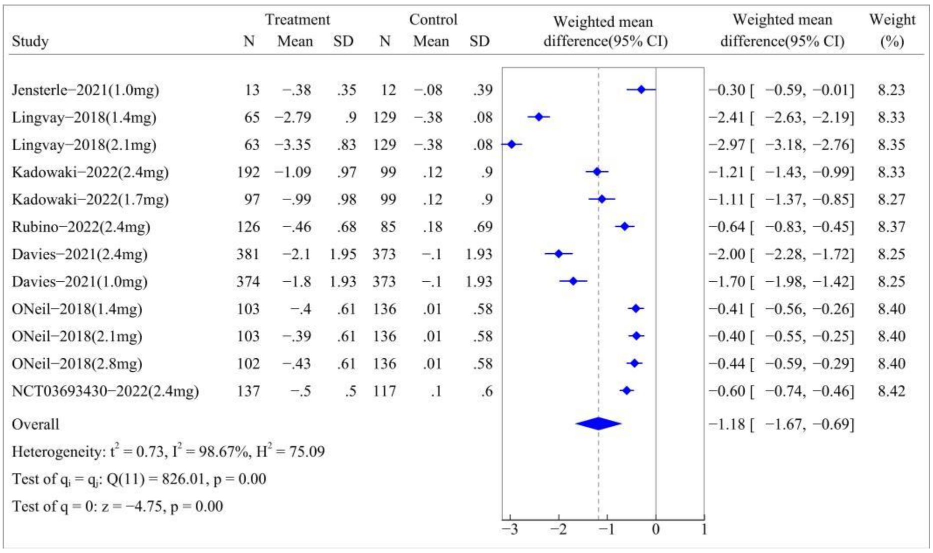

Supplementary Figure 9 Weighted mean difference in change in FPG between the semaglutide and placebo control

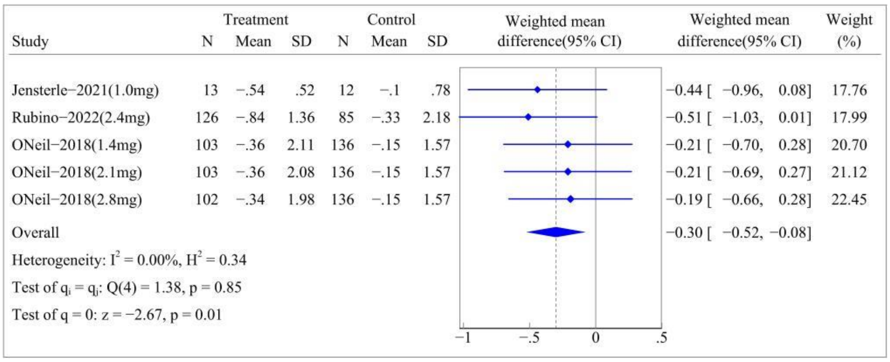

Supplementary Figure 10 Weighted mean difference in change in TC between the semaglutide and placebo control

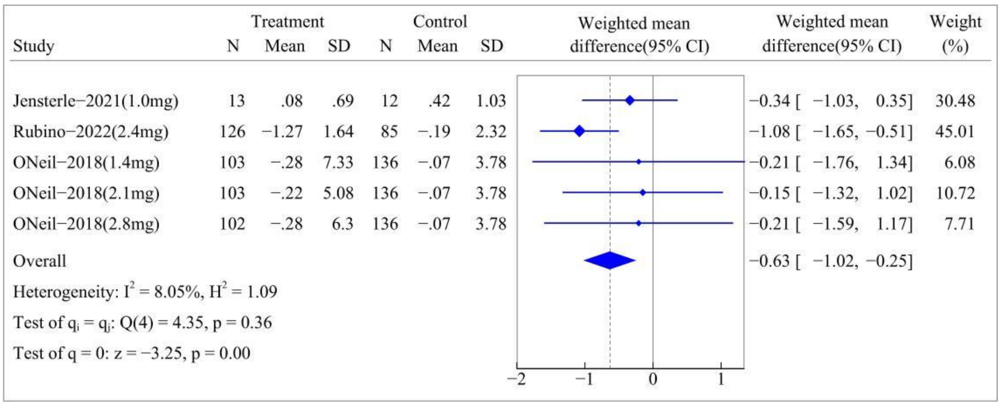

Supplementary Figure 11 Weighted mean difference in change in TG between the semaglutide and placebo control

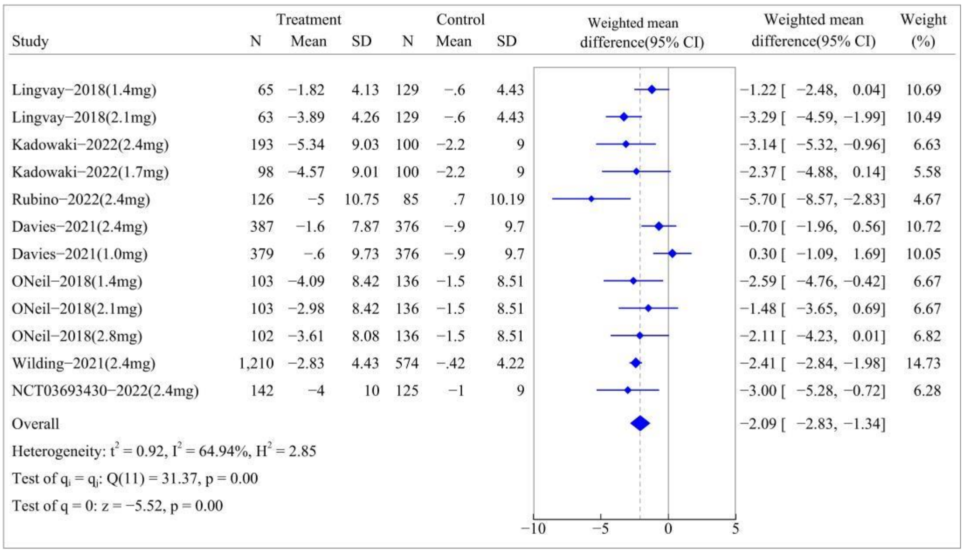

Supplementary Figure 12 Weighted mean difference in change in DBP between the semaglutide and placebo control

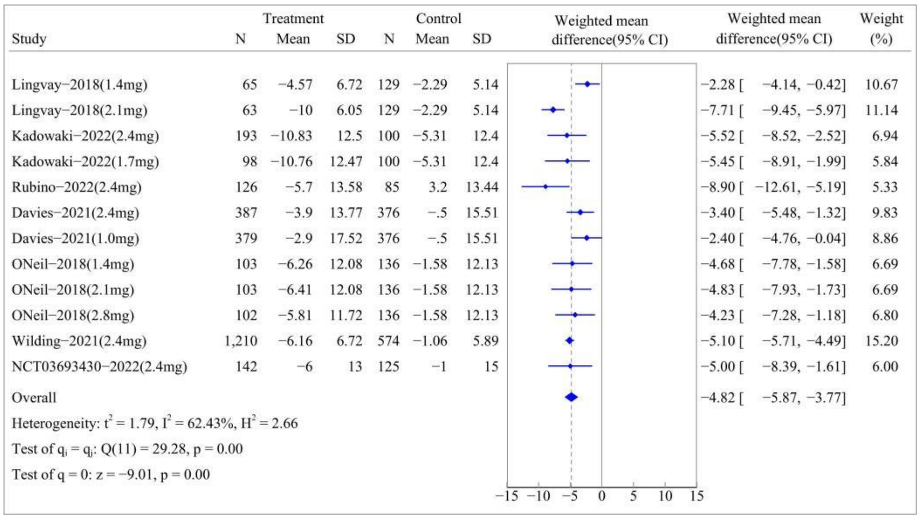

Supplementary Figure 13 Weighted mean difference in change in SBP between the semaglutide and placebo control

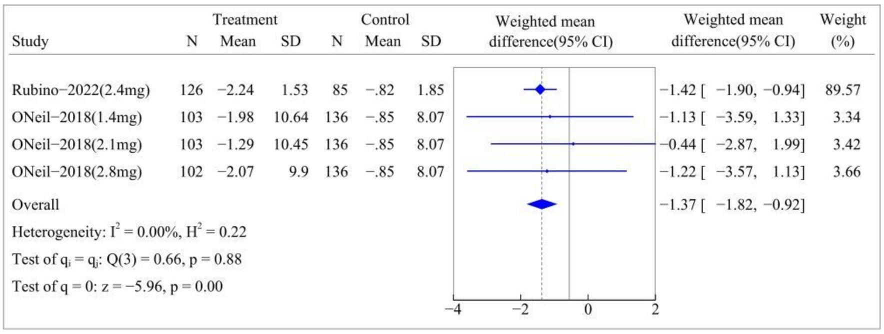

Supplementary Figure 14 Weighted mean difference in change in CRP between the Semaglutide and placebo control.

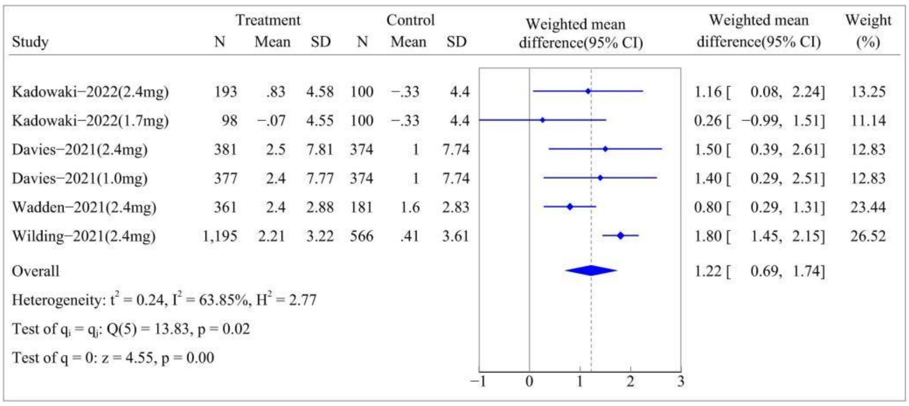

Supplementary Figure 15 Weighted mean difference in change in SF-36v2 between the semaglutideand placebo control

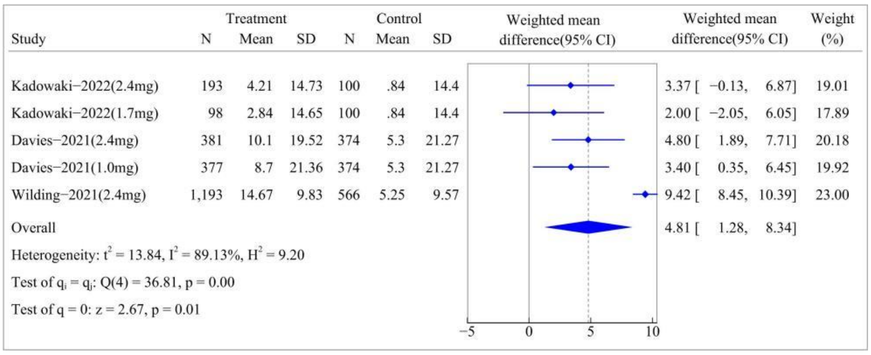

Supplementary Figure 16 Weighted mean difference in change in IWQOL-Lite-CT between the semaglutide and placebo control

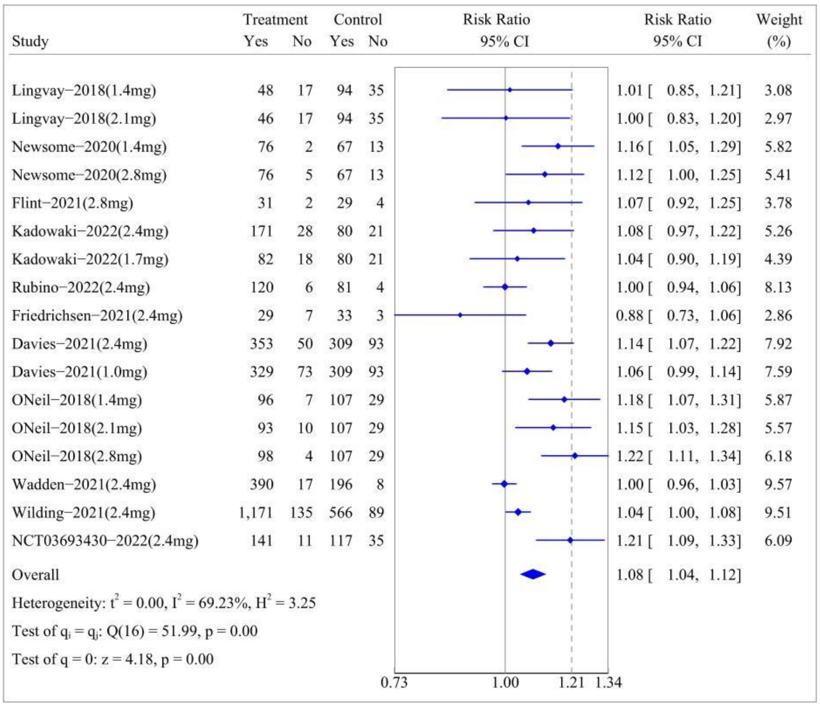

Supplementary Figure 17 Risk ratio in total adverse events between the semaglutide and placebo control

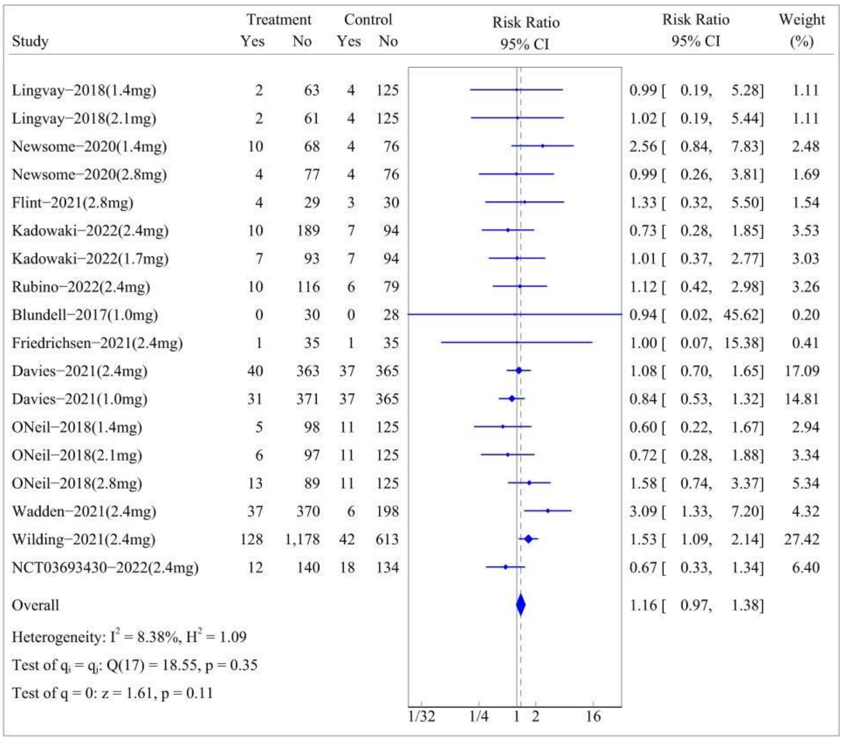

Supplementary Figure 18 Risk ratio in severe adverse events between the semaglutide and placebo control

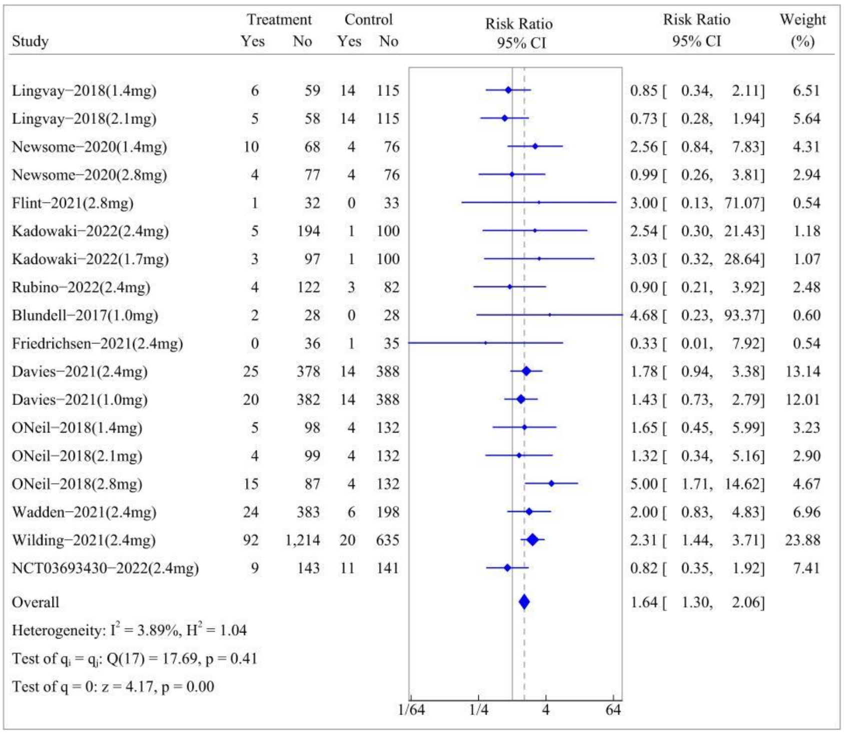

Supplementary Figure 19 Risk ratio in adverse events leading to discontinuation

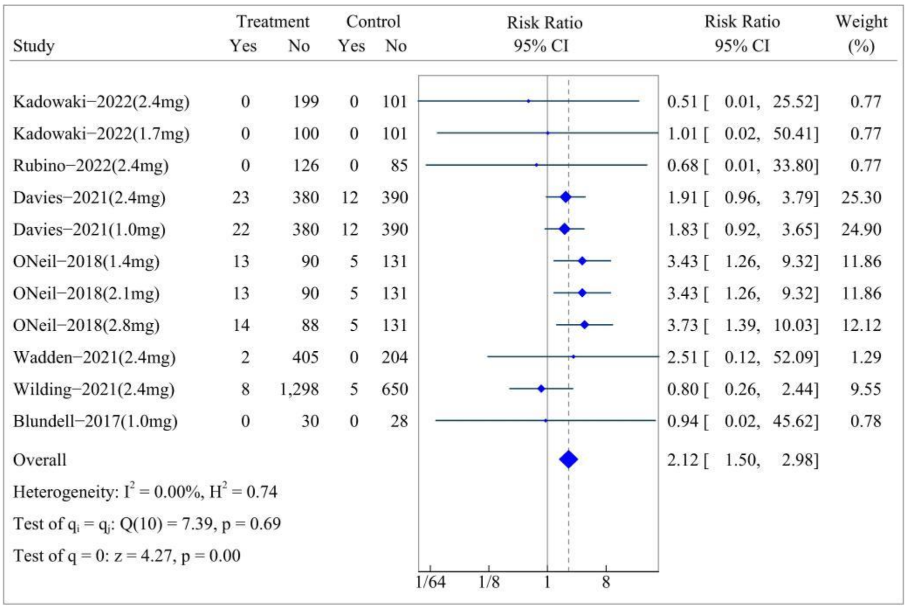

Supplementary Figure 20 Risk ratio in hypoglycemia between the semaglutide and placebo control

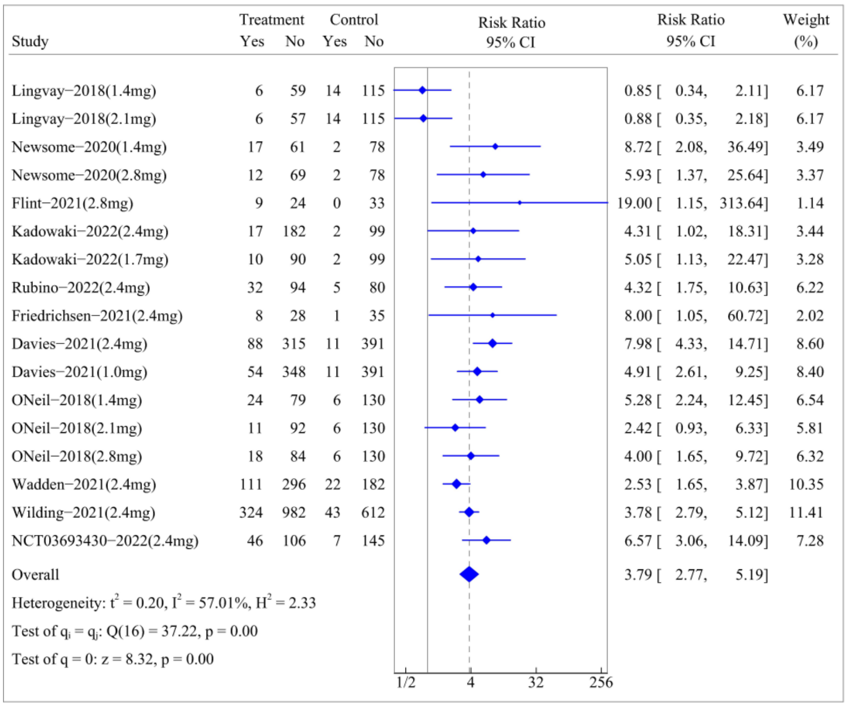

Supplementary Figure 21 Risk ratio in vomiting between the semaglutide and placebo control

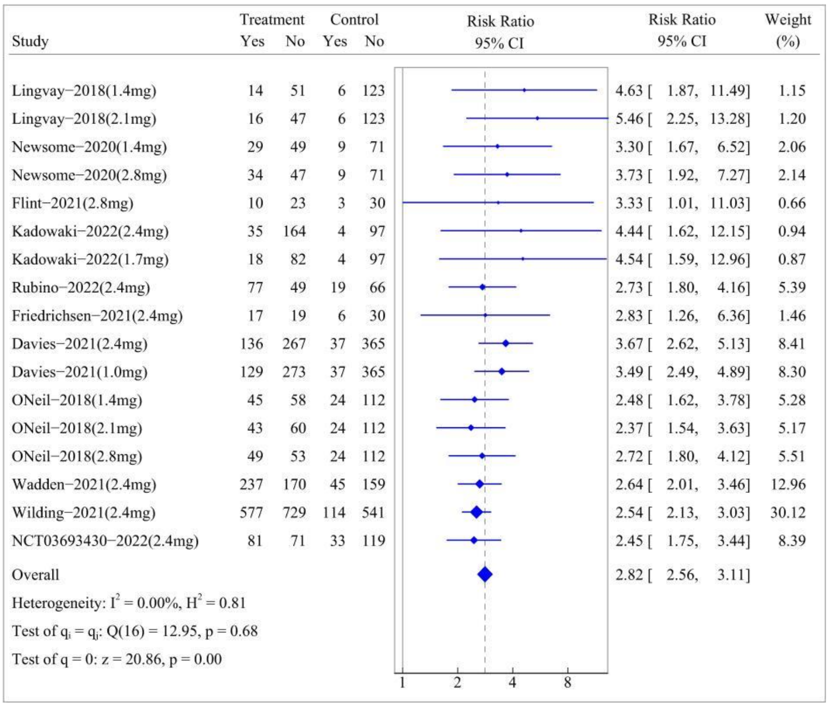

Supplementary Figure 22 Risk ratio in nausea between the semaglutide and placebo control

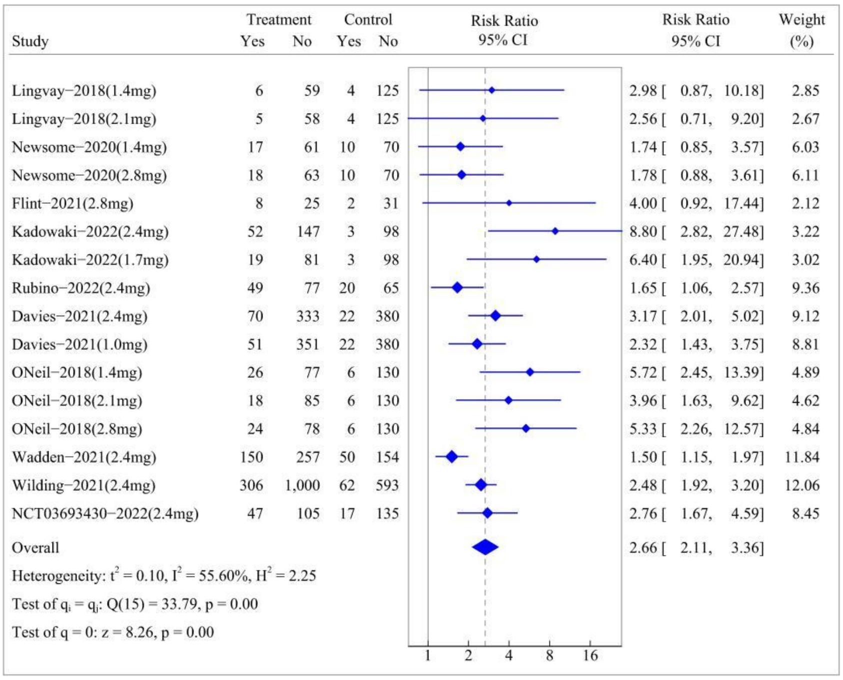

Supplementary Figure 23 Risk ratio in constipation between the semaglutide and placebo control

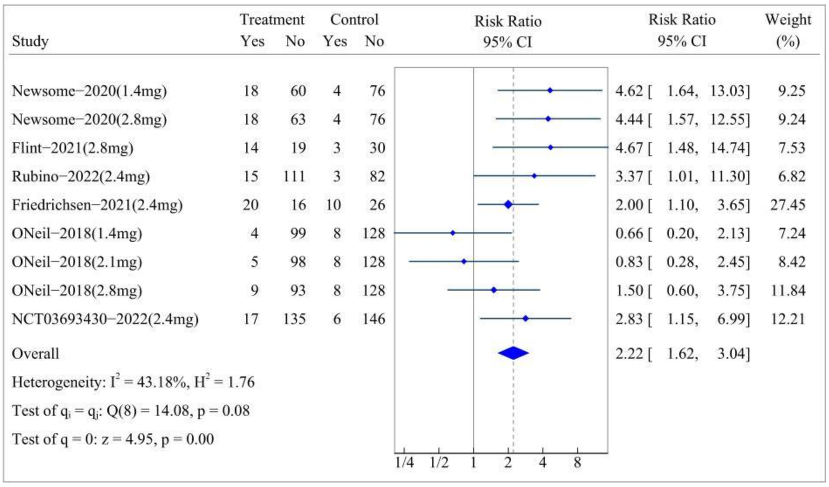

Supplementary Figure 24 Risk ratio in loss of appetite between the semaglutide and placebo control

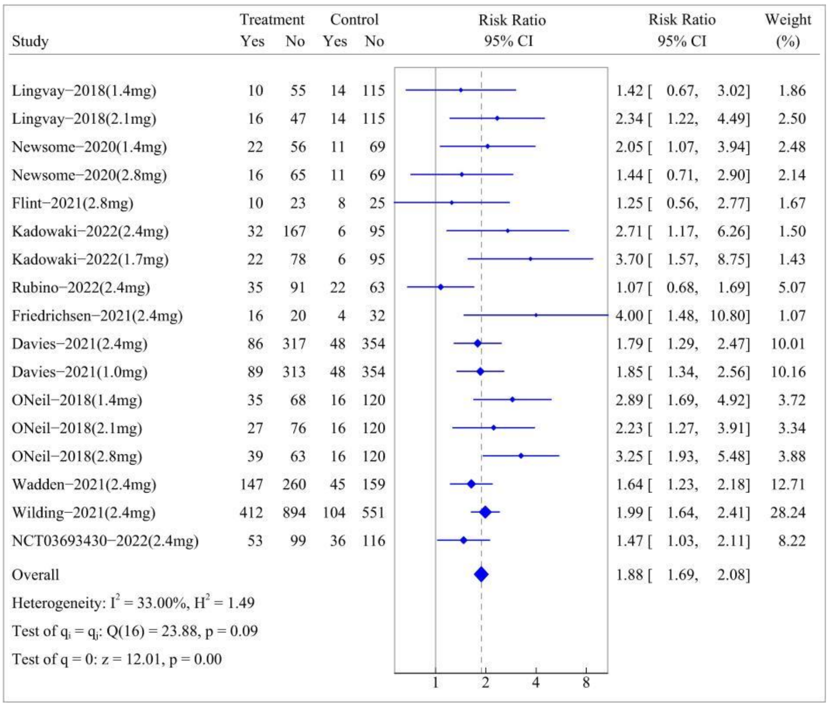

Supplementary Figure 25 Risk ratio in diarrhea between the semaglutide and placebo control

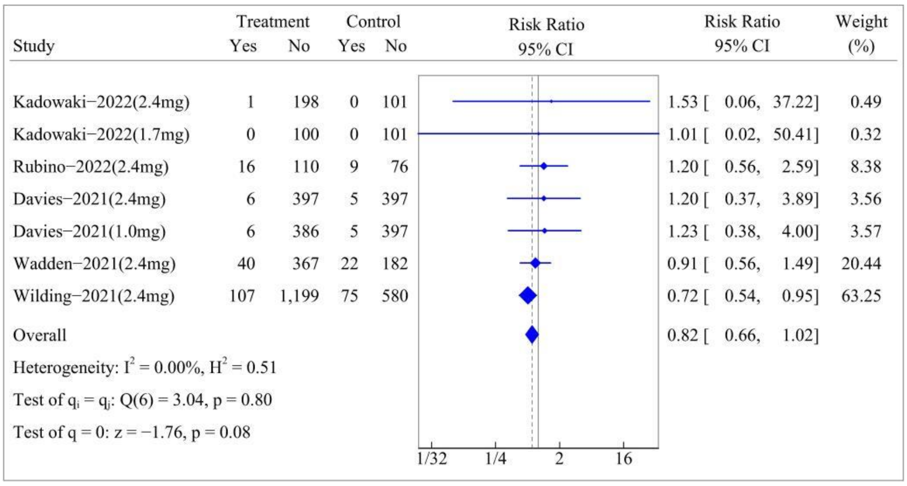

Supplementary Figure 26 Risk ratio in cardiovascular disorders between the semaglutide and placebo control

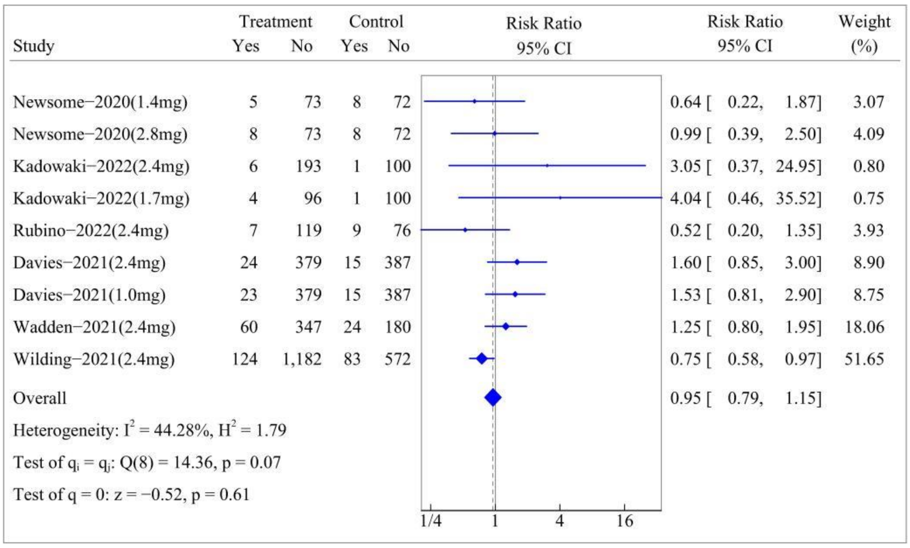

Supplementary Figure 27 Risk ratio in psychiatric disorders between the semaglutide and placebo control

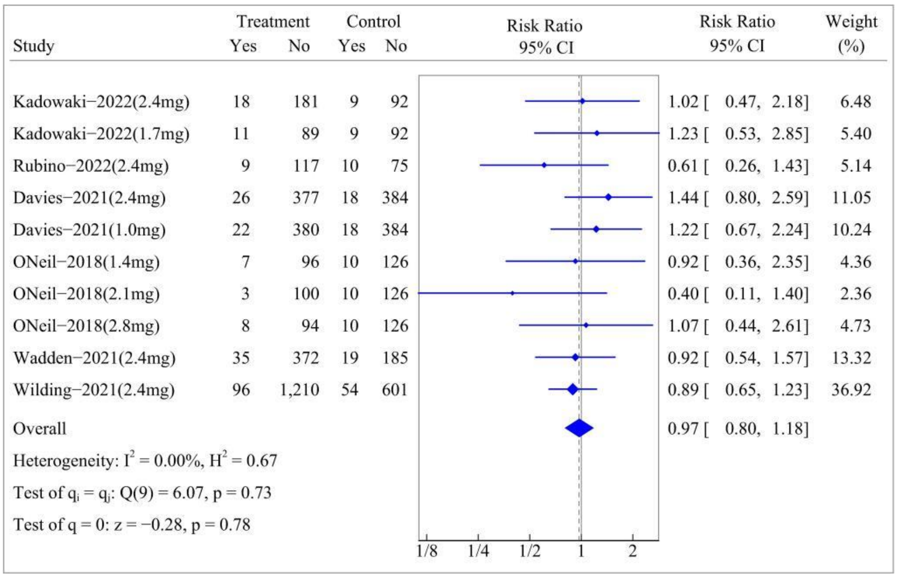

Supplementary Figure 28 Risk ratio in allergic reactions between the semaglutide and placebo

control

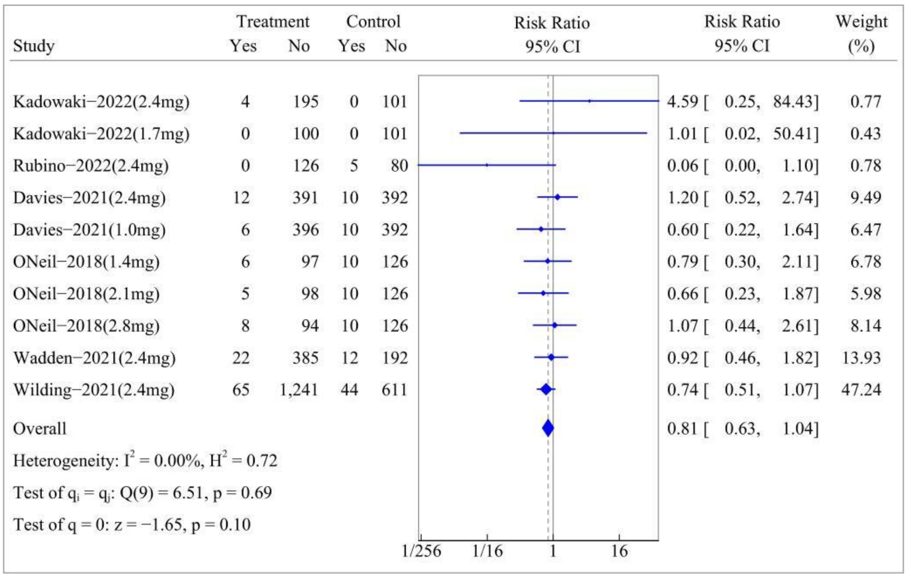

Supplementary Figure 29 Risk ratio in injection-site reactions between the semaglutide and placebo control

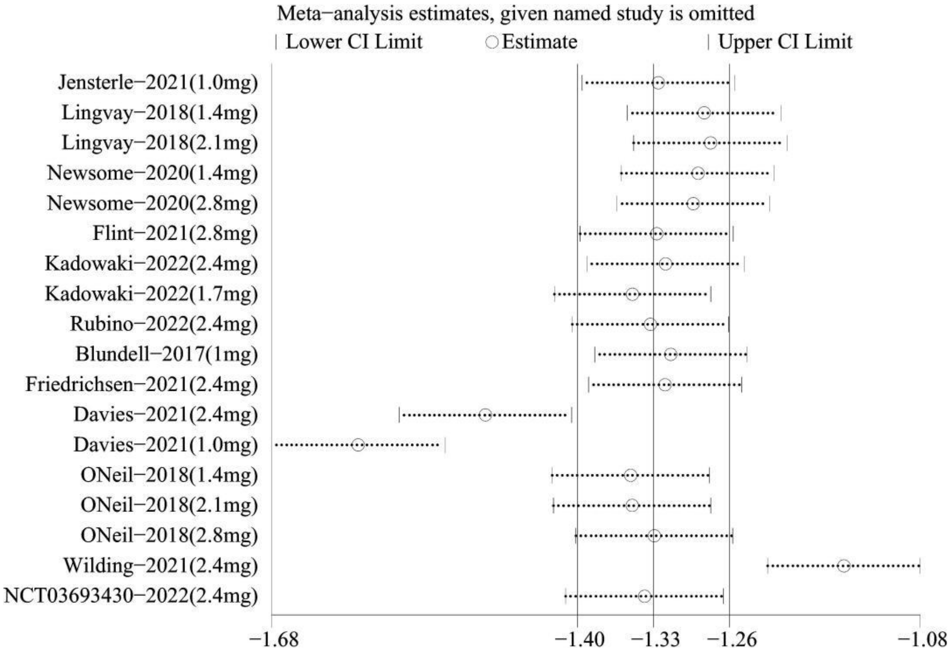

Supplementary Figure 30 Sensitivity analysis of the absolute change in body weight

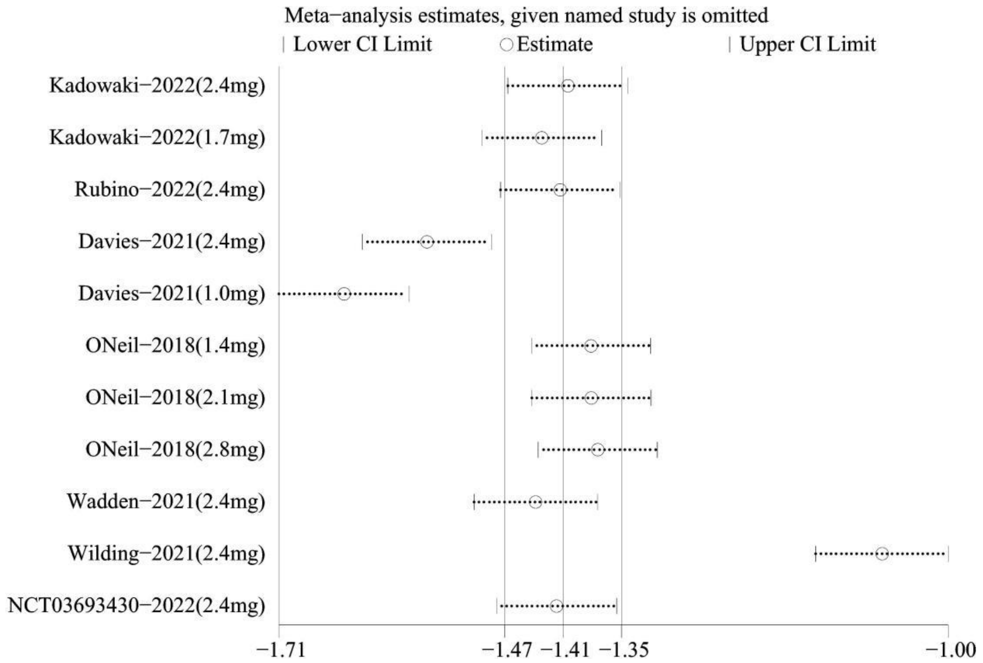

Supplementary Figure 31 Sensitivity analysis of the percentage change in body weight

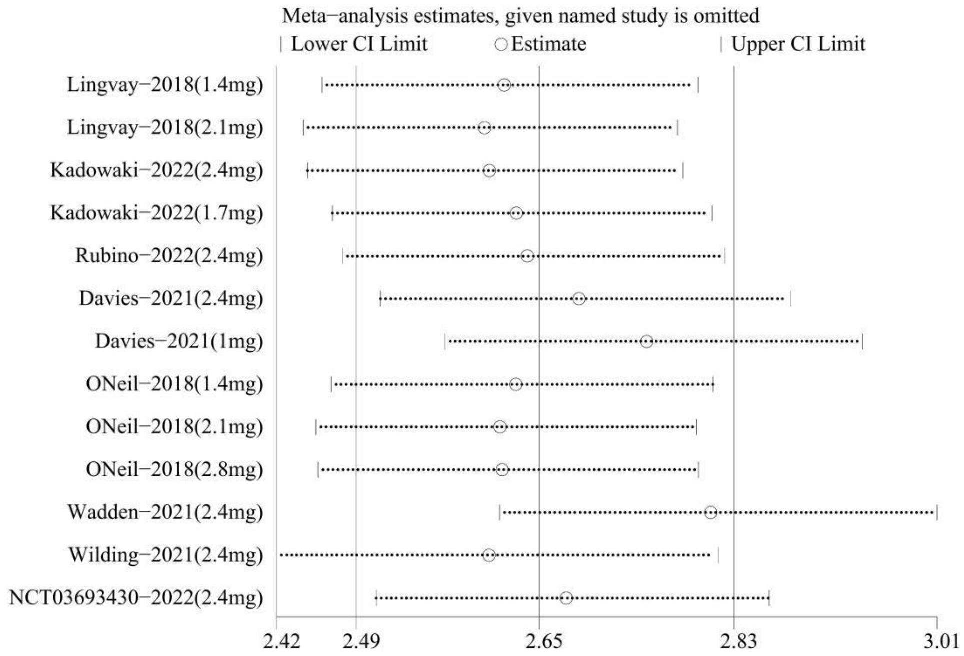

Supplementary Figure 32 Sensitivity analysis of weight reduction of at least 5%

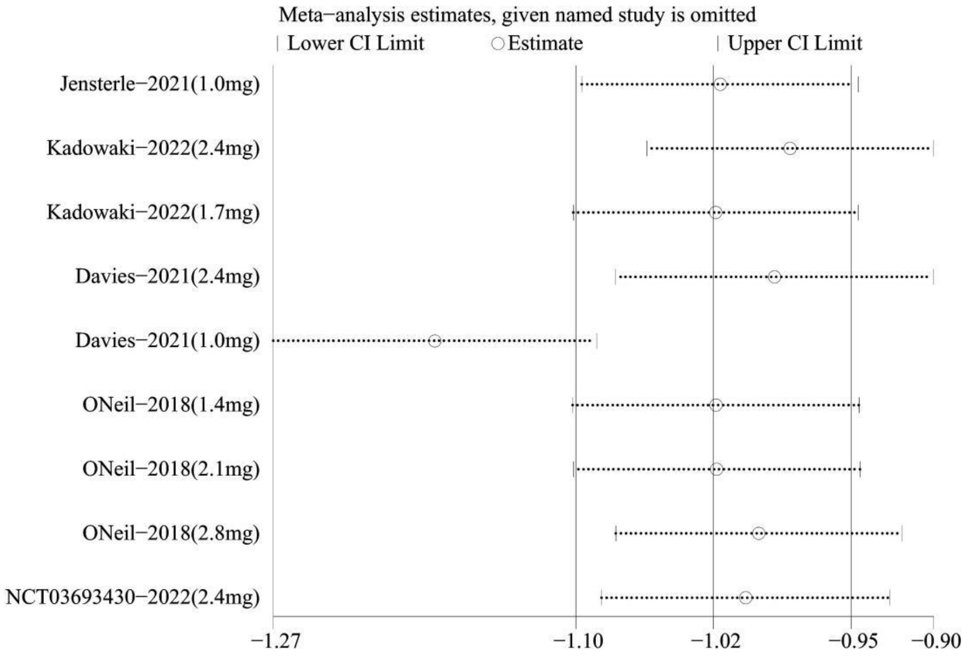

Supplementary Figure 33 Sensitivity analysis of change in BMI

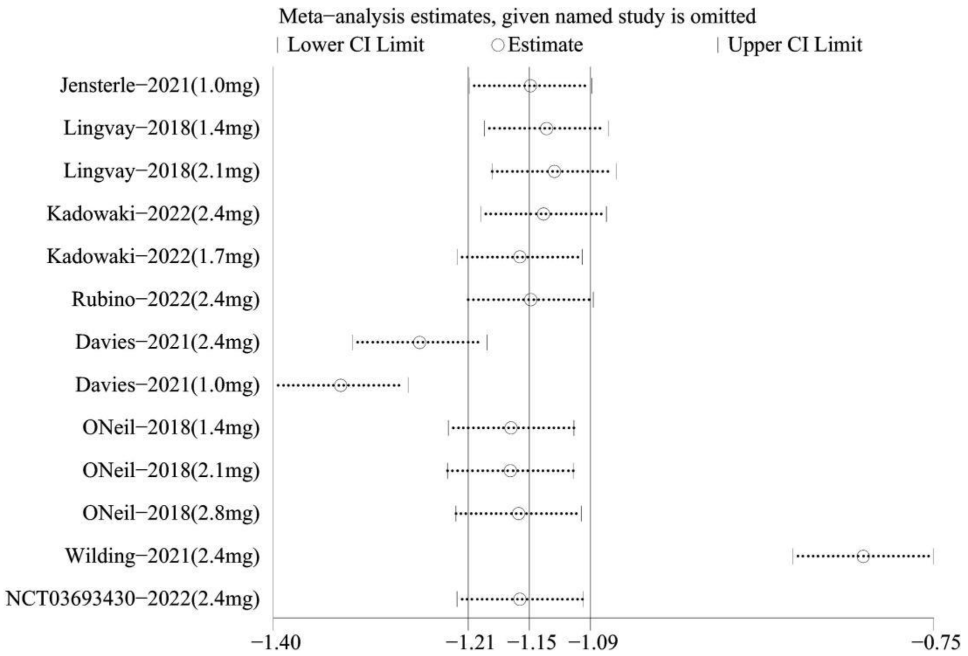

Supplementary Figure 34 Sensitivity analysis of change in waist circumference

a

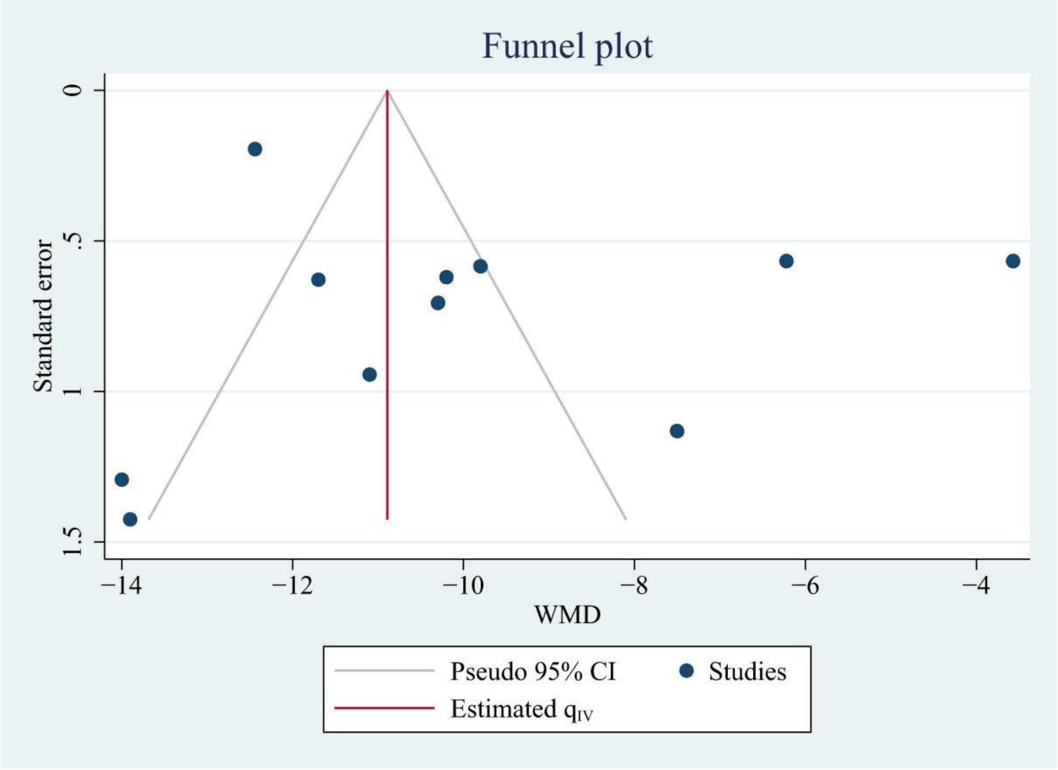

Funnel plot
Standard error
0
.5
1
1.5
−14
−12
−10
−8
−6
−4
WMD
Pseudo 95% CI
Studies
Estimated q_{IV}

b

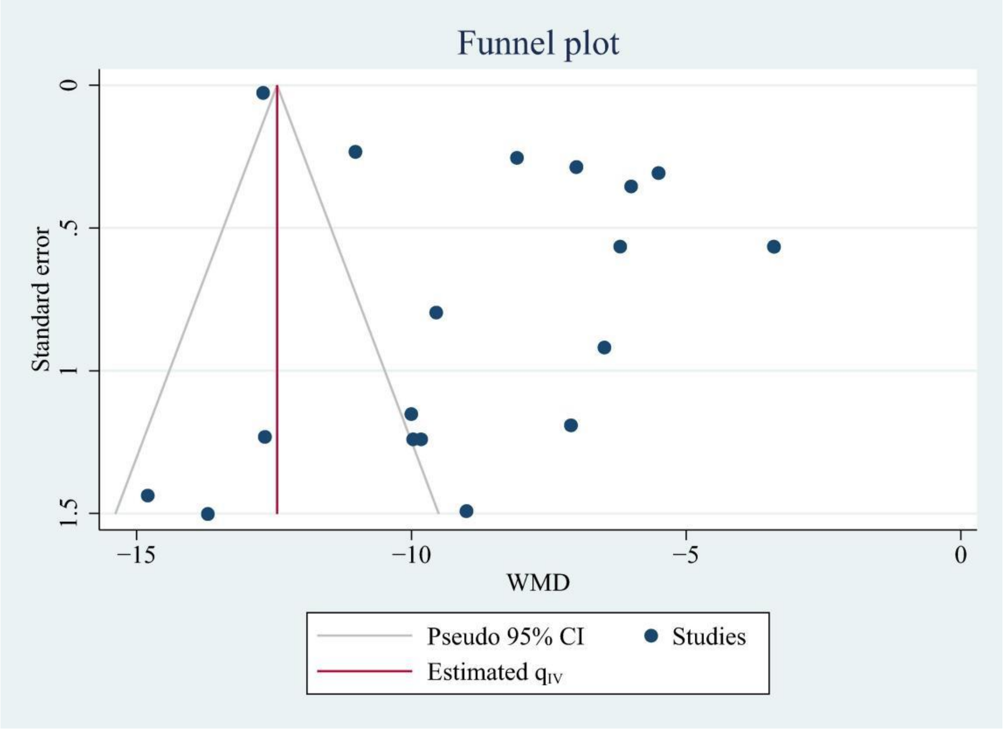

Funnel plot
Standard error
0
.5
1
1.5
−15
−10
−5
0
WMD
Pseudo 95% CI
Studies
Estimated q_{IV}

c

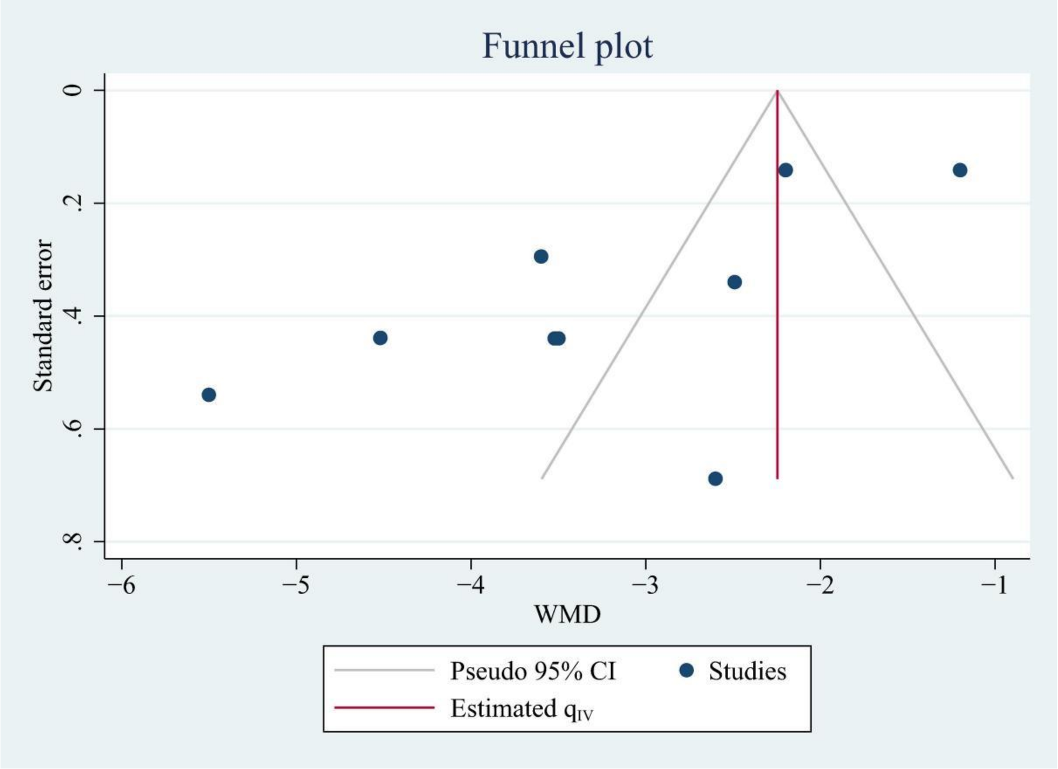

d

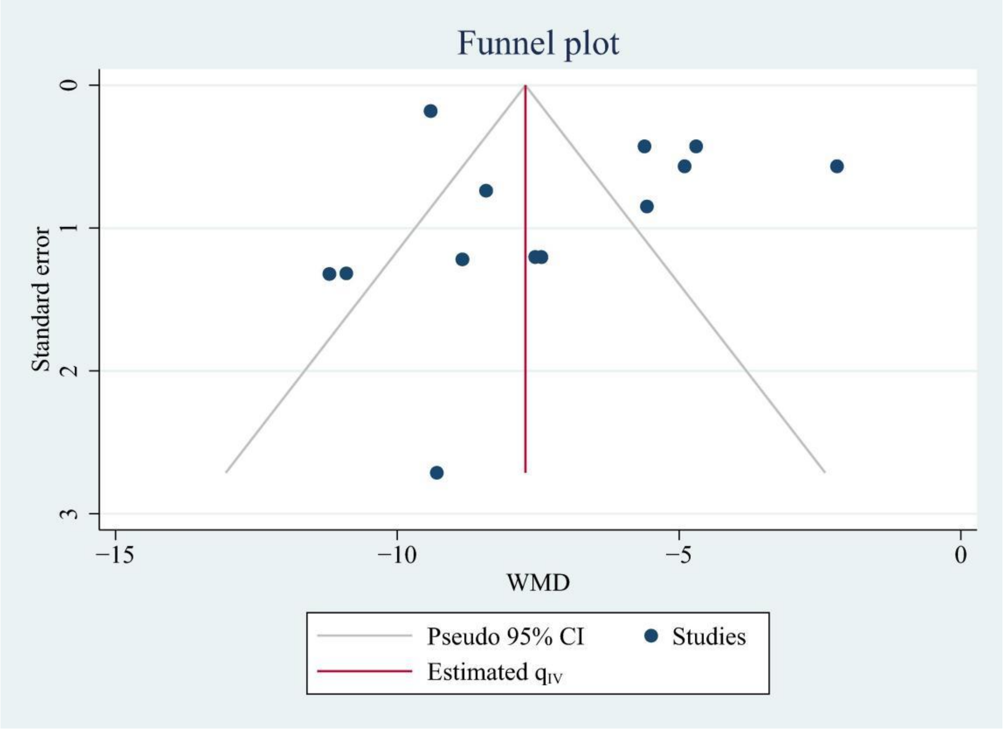

Supplementary Figure 35 Publication bias funnel plots for weight related index. a. Publication bias funnel plot for percentage change in body weight; b.Publication bias funnel plots for absolute change in body weight; c.Publication bias funnel plot for BMI; d.Publication bias funnel plot for waist circumference.

a

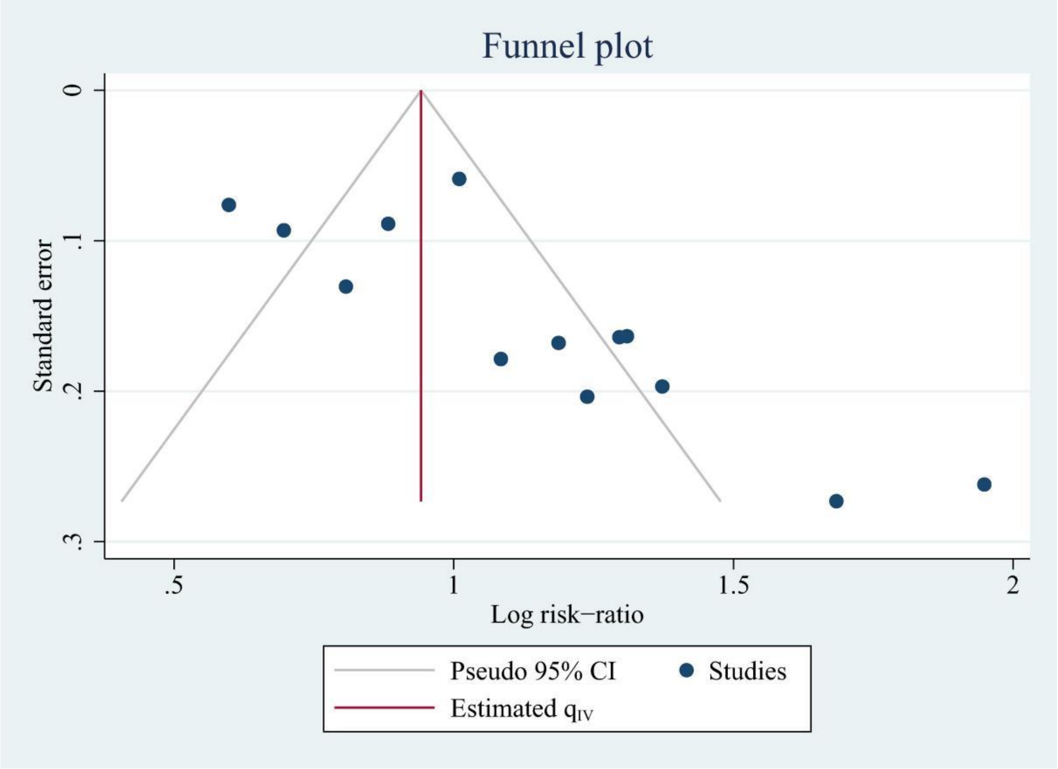
Funnel plot
Standard error
0
.1
.2
.3
.5
1
1.5
2
Log risk−ratio
Pseudo 95% CI
Studies
Estimated q_{IV}

b

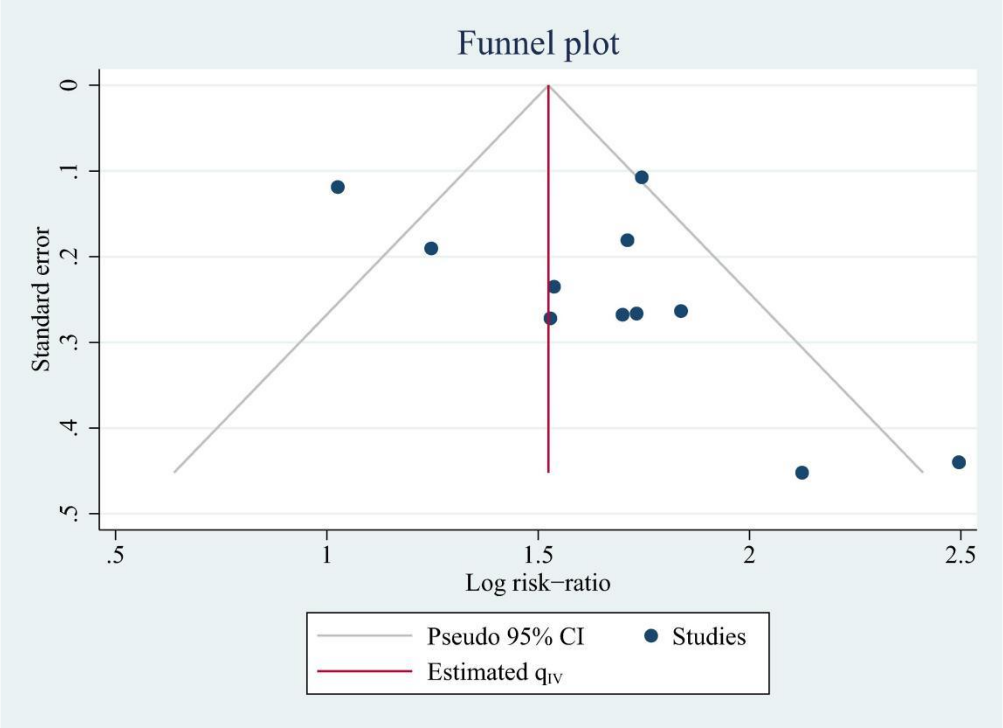
Funnel plot
Standard error
0
.1
.2
.3
.4
.5
.5
1
1.5
2
2.5
Log risk−ratio
Pseudo 95% CI
Studies
Estimated q_{IV}

c

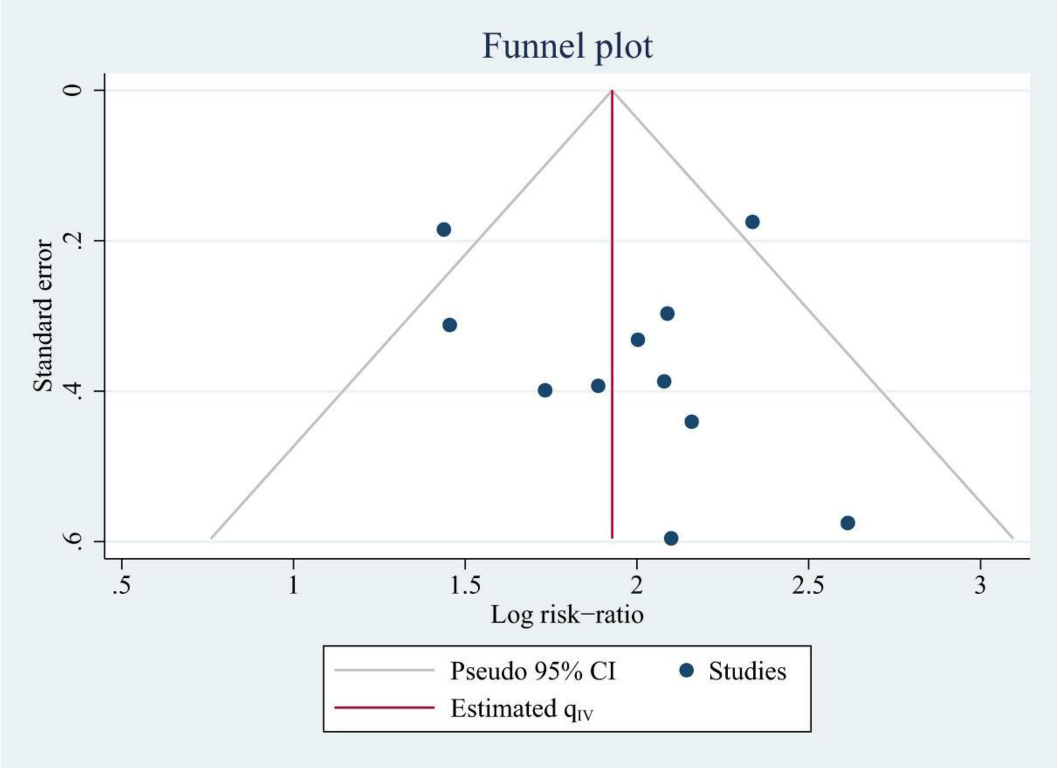

Supplementary Figure 36 Publication bias funnel plots for achievement of categorical weight loss targets. a. Publication bias funnel plot for weight loss goal of 5% ; b. Publication bias funnel plot for weight loss goal of 10%; c. Publication bias funnel plot for weight loss goal of 15%.

a

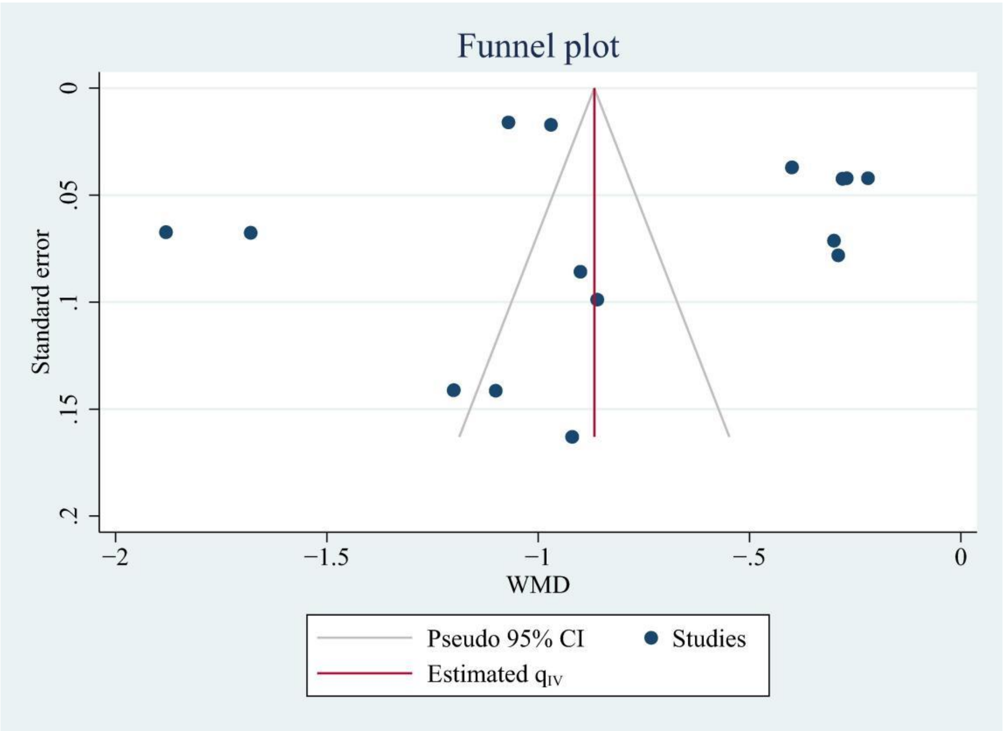

b

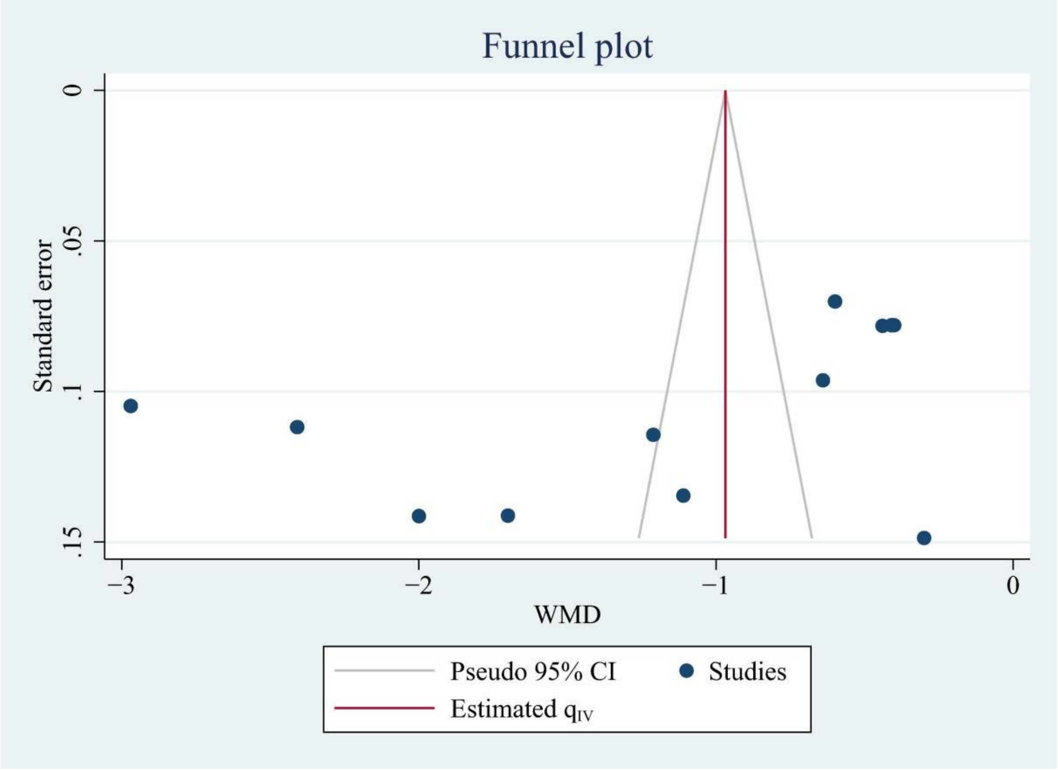

Supplementary Figure 37 Publication bias funnel plots for blood glucose. a. Publication bias funnel plots for HbA1c; b. Publication bias funnel plot for FPG.

a

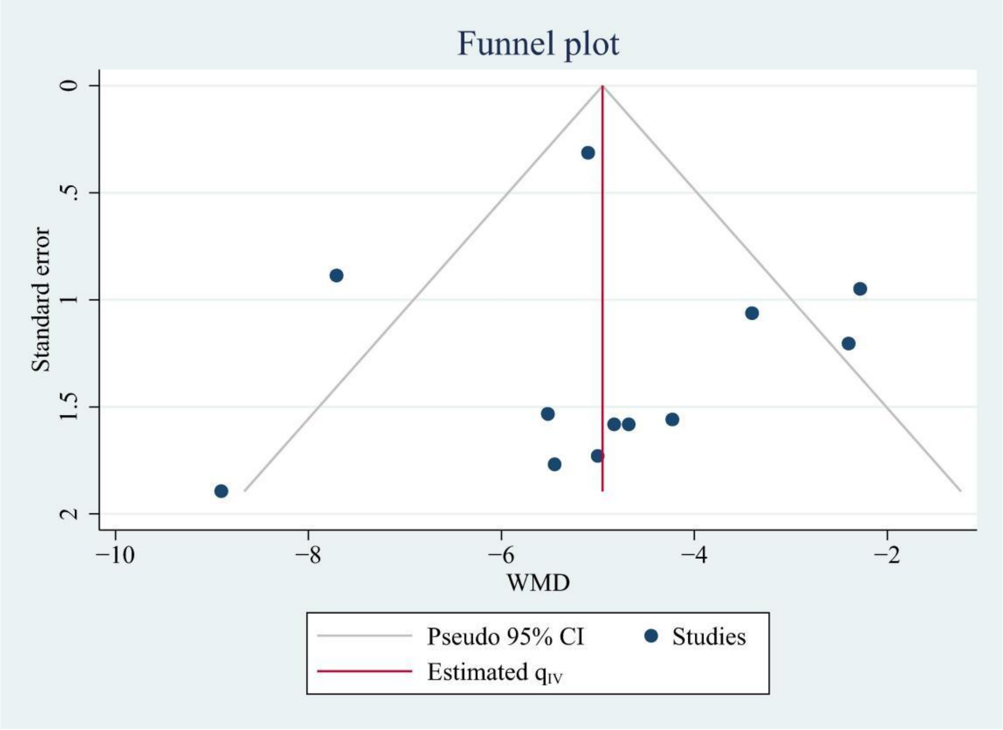

b

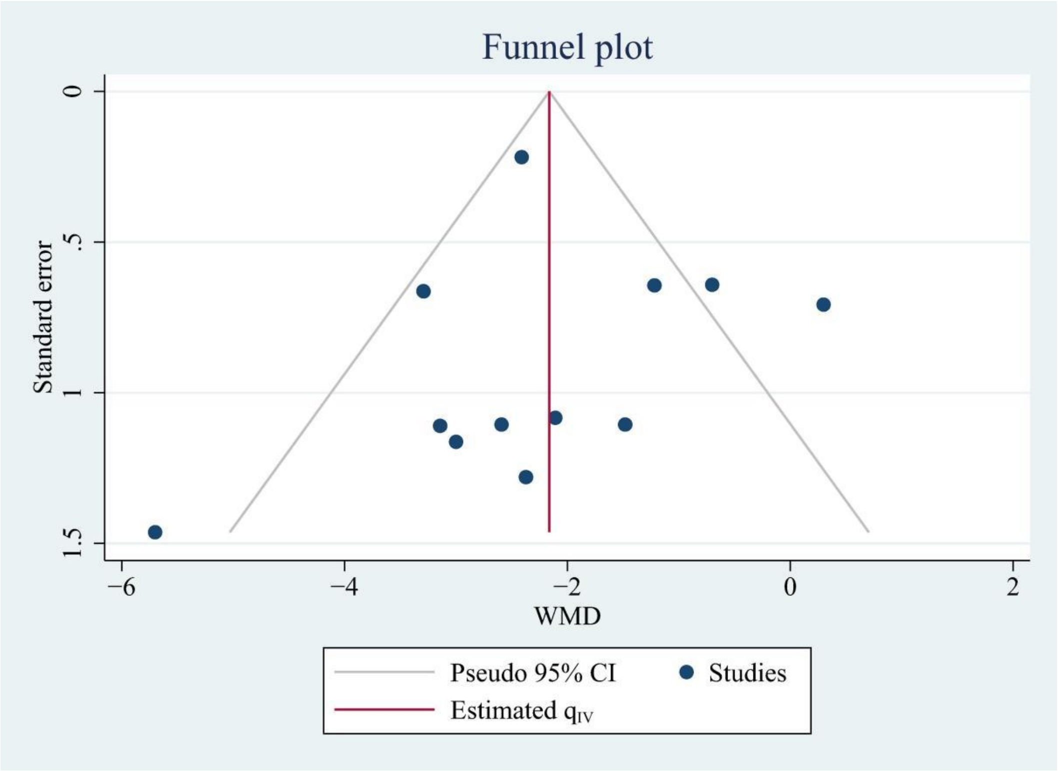

Supplementary Figure 38 Publication bias funnel plots for blood pressure. a. Publication bias funnel plot for SBP ; b. Publication bias funnel plot for DBP.

a

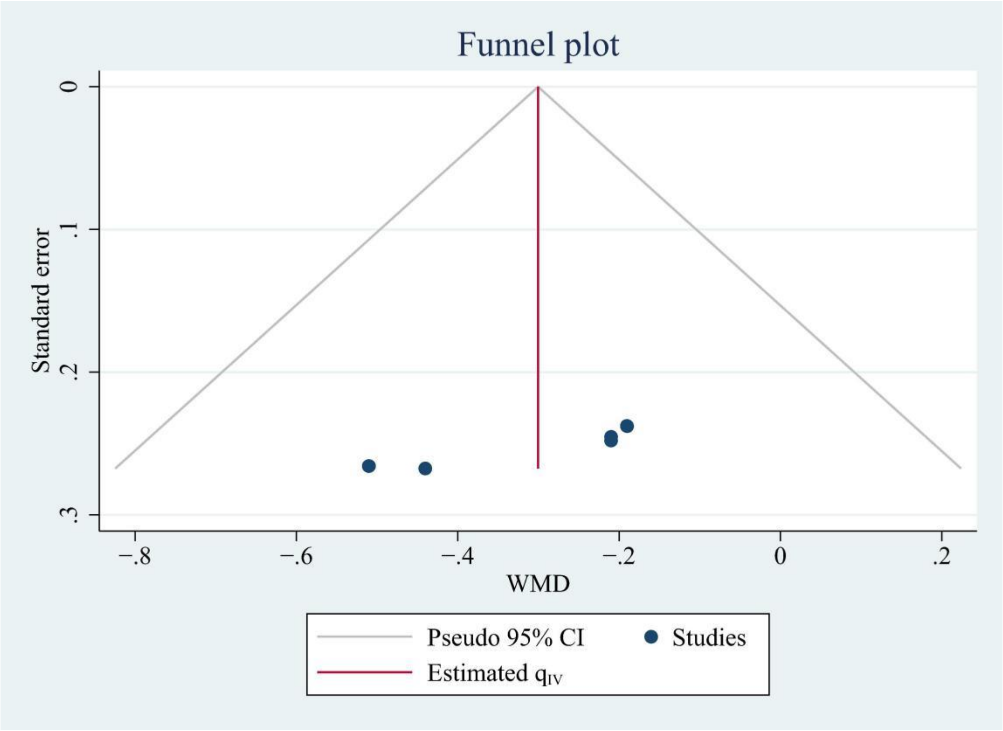

b

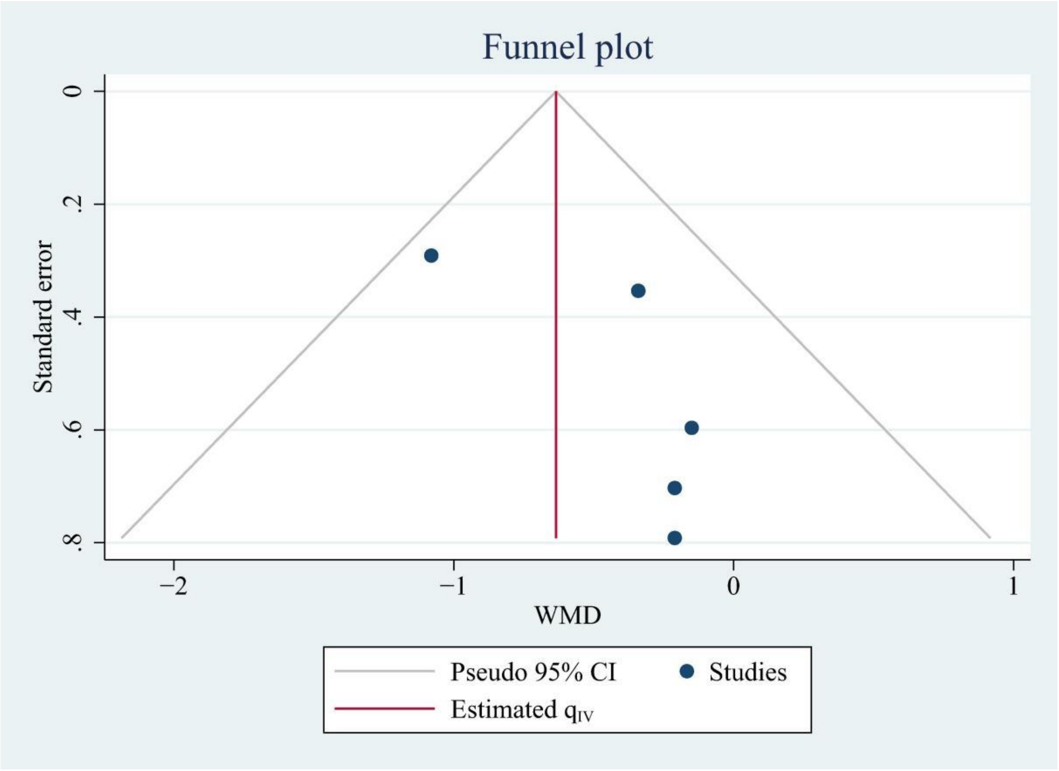

Supplementary Figure 39 Publication bias funnel plots for bllipids. a. Publication bias funnel plot for TC; b. Publication bias funnel plot for TG.

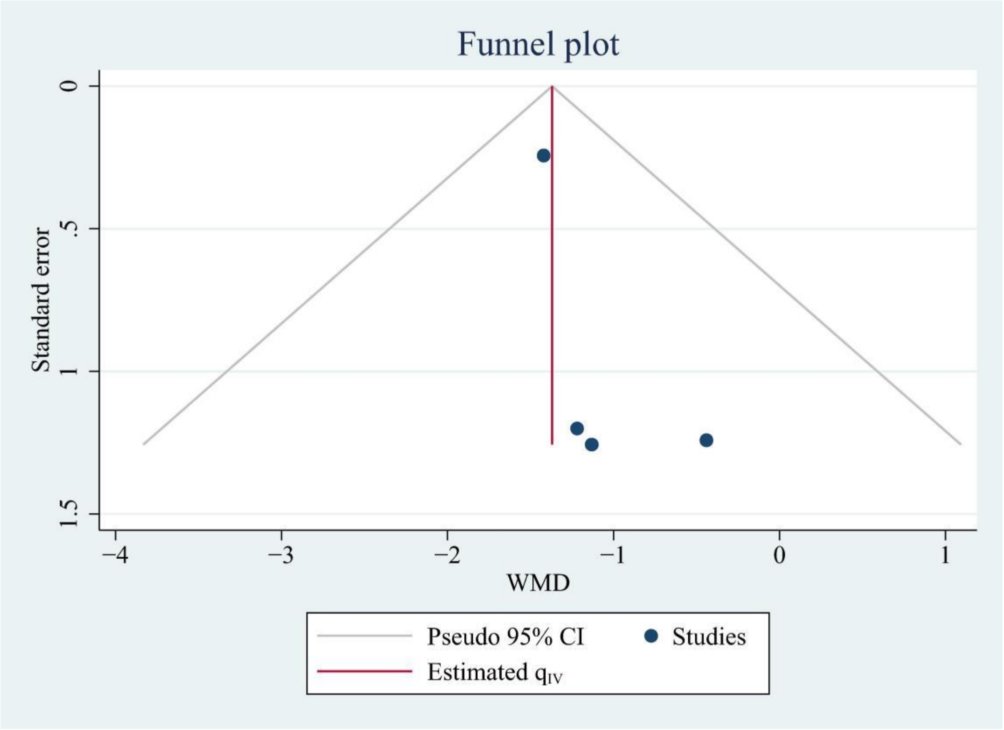

Supplementary Figure 40 Publication bias funnel plots for CRP.

a

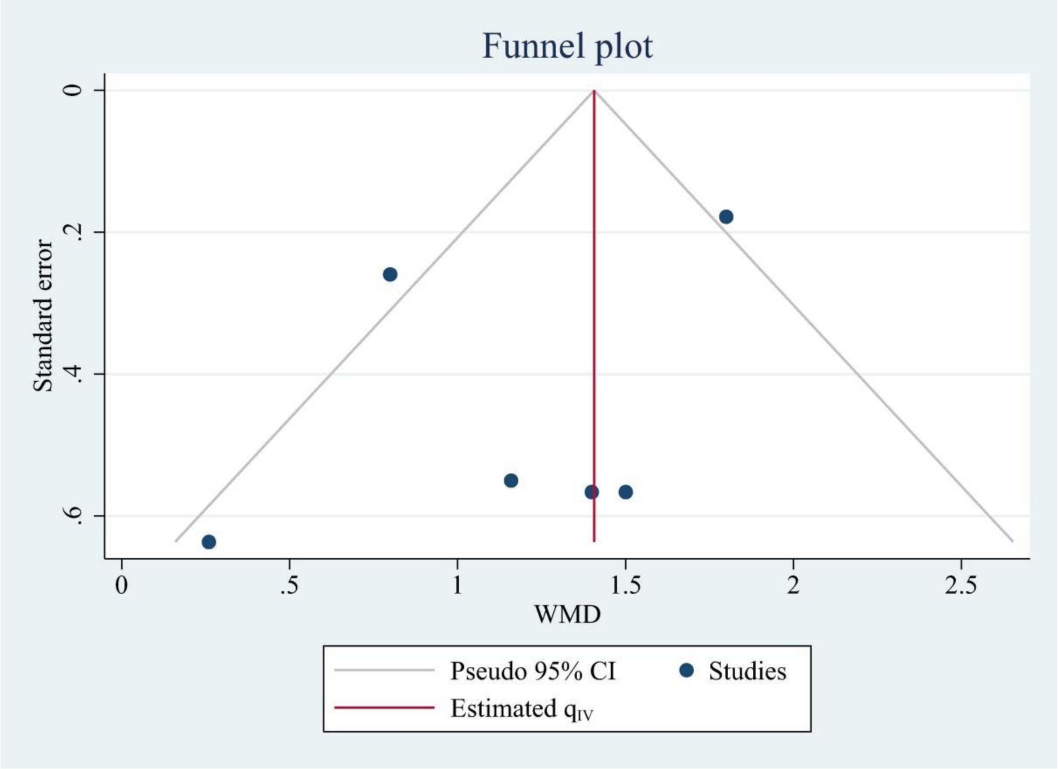

b

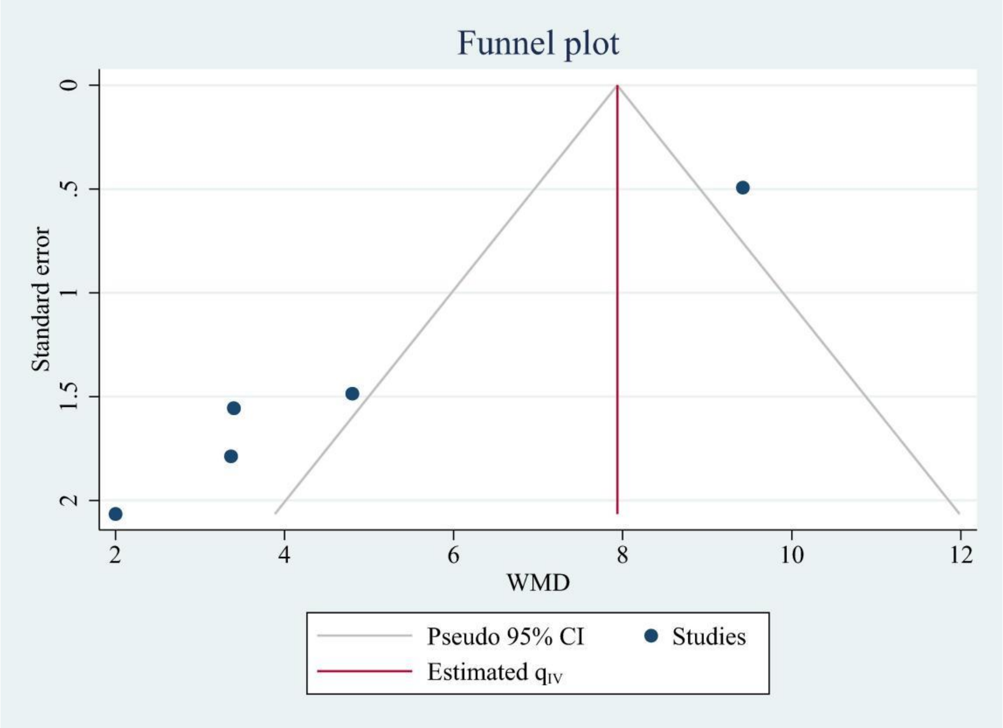

Supplementary Figure 41 Publication bias funnel plots for patient-reported outcomes of quality of life. a. Publication bias funnel plot for SF-36v2; b. Publication bias funnel plot for IWQOL-Lite-CT.
