# Supplementary material for: Efficacy and safety of subcutaneous semaglutide in adults with overweight or obese: a subgroup meta-analysis of randomized controlled trials
Source: Front Endocrinol (Lausanne). 2023 Jun 26;14:1132004. doi: 10.3389/fendo.2023.1132004 (PMC10338217; doi:10.3389/fendo.2023.1132004)
Supplement: Supplementary file 1 [file DataSheet_1.zip › Data Sheet 1/supplementary/cover letter.doc]

《**Frontiers in endocrinplogy》**

26 December; 2022

Dear editor,

We are submitting a manuscript entitled “Efficacy and safety of subcutaneous semaglutide in overweight or obese adults: A subgroup meta-analysis of randomized controlled trials” for your consideration for publication as a Research in frontiers in endocrinplogy. This study was implemented according to a priori-written protocol registered in PROSPERO (CRD42022337099, registered 13 June 2022) and the reporting was followed by the Preferred Reporting Items for Systematic Reviews and Meta-analysis (PRISMA).

Several Trials have demonstrated semaglutide treatment is an effective and safe intervention for obese or overweight people. But it is unclear what is the superior therapeutic effect of semaglutide under diverse application circumstances. So we analyzed the efficacy and safety of subcutaneous semaglutide under the various situations, including different dosages, administration frequencies, trial durations, baseline obesity classifications, and whether accompanied with lifestyle interventions.

Our results found that subcutaneous semaglutide treatment had great beneficial effect on body weight control and considerable safety for overweight or obese adults. Besides the weight loss, additional improvements on cardiovascular and metabolic profiles, such as blood glucose, blood pressure, and serum lipids were also observed. Based on subgroup analysis, we found that semaglutide treatment could achieve superior effect when coupled with lifestyle interventions, and target dose of 2.0 mg or more subcutaneously once weekly.

We believe that this meta-analysis is appropriate for publication by frontiers in endocrinplogy. It would be of great interest to a broad range of readers including clinicians, patients, policy makers and researchers on related fields. For clinicians, they will be provided more helpful information to choose the appropriate treatment scheme for patients. Policy makers can take advantage of our results as a reference for good pharmacological decision making.

We confirm that this manuscript has not been published elsewhere and is not under consideration by another journal. All authors have approved the manuscript and agree with submission to frontiers in endocrinplogy. This study is supported by grants from The National Key Technology R&D Program of China (2017YFC0113901), The Key R&D Program of Department of Science and Technology of Sichuan Province (2022YFS0600 )；The Science and Technology Bureau of Chengdu City （2021-YF05-00498-SN）.The authors have no conflicts of interest to declare.

Thanks for your time and consideration. We look forward to hearing from you at your earliest convenience.

Sincerely,

Ping Shuai

Department of Health Management & Physical Examination, Sichuan Provincial People's Hospital, University of Electronic Science and Technology of China, Chengdu, China

W. Sec 2, 1st Ring Rd, Chengdu, China

shuaiping@med.uestc.edu.cn

0086-457-28-87394692
